# Fabrications and Applications of Stimulus-Responsive Polymer Films and Patterns on Surfaces: A Review

**DOI:** 10.3390/ma7020805

**Published:** 2014-01-28

**Authors:** Jem-Kun Chen, Chi-Jung Chang

**Affiliations:** 1 Department of Materials Science and Engineering, National Taiwan University of Science and Technology, 43, Section 4, Keelung Road, Taipei 106, Taiwan; E-Mail: jkchen@mail.ntust.edu.tw; 2 Department of Chemical Engineering, Feng Chia University, 100 Wenhwa Road, Seatwen, Taichung 40724, Taiwan

**Keywords:** thermoresponsive, pH-responsive, photo-responsive, polymer, magnetically-responsive

## Abstract

In the past two decades, we have witnessed significant progress in developing high performance stimuli-responsive polymeric materials. This review focuses on recent developments in the preparation and application of patterned stimuli-responsive polymers, including thermoresponsive layers, pH/ionic-responsive hydrogels, photo-responsive film, magnetically-responsive composites, electroactive composites, and solvent-responsive composites. Many important new applications for stimuli-responsive polymers lie in the field of nano- and micro-fabrication, where stimuli-responsive polymers are being established as important manipulation tools. Some techniques have been developed to selectively position organic molecules and then to obtain well-defined patterned substrates at the micrometer or submicrometer scale. Methods for patterning of stimuli-responsive hydrogels, including photolithography, electron beam lithography, scanning probe writing, and printing techniques (microcontact printing, ink-jet printing) were surveyed. We also surveyed the applications of nanostructured stimuli-responsive hydrogels, such as biotechnology (biological interfaces and purification of biomacromoles), switchable wettability, sensors (optical sensors, biosensors, chemical sensors), and actuators.

## 1. Introduction

Mother Nature shows us abundant examples of stimuli-responsive (or smart) materials. The leaves of Mimosa pudica collapse suddenly when touched, and those of the Venus flytrap snap shut on doomed insect prey; the leaflets of Codariocalyx motorius rotate, and sunflowers turn toward the sun; and chameleons change color according to their environment. At their most fundamental level, many of the most important substances in living systems are macromolecules with structures and behaviors that vary according to the conditions in the surrounding environment. Mimicking the functions of such organisms, scientists have made great efforts to synthesize stimuli-responsive polymers that have significance to science and promising applications. Incorporating multiple copies of functional groups that are readily amenable to a change in character (e.g., charge, polarity, and solvency) along a polymer backbone causes relatively minor changes in chemical structure to be synergistically amplified to bring about dramatic transformations in macroscopic material properties.

Polymers such as proteins, polysaccharides, and nucleic acids are present as basic components in living organic systems. Synthetic polymers, which are designed to mimic these biopolymers, have been developed into a variety of functional forms to meet industrial and scientific applications. These synthetic polymers can be classified into different categories based on their chemical properties. Certain special types of polymers have emerged as very useful class of polymers having their own special chemical properties and applications in various areas. These “stimuli-responsive” polymers (SRPs) have been variously called stimuli-sensitive [1], intelligent [2], smart [3,4], or environmentally-sensitive polymers [5]. SRPs can rapidly change shape with respect to configuration or dimension under the influence of stimuli such as temperature [6], pH value [7,8], light [9], magnetic field [10], electricity [11], and solvent/water [12]. These polymers can also have different compositions and architecture, including not only homopolymers [13] but also statistical/block copolymers [14], graft copolymers, and molecular brushes. They can be also grafted on/from surfaces [15] or be used as chemically or physically cross-linked gels [16]. SRPs are usually capable of stimuli-induced conformational changes, reversible solubility control [17], and reversible self-assembly into polymeric micelles or vesicles. Given these unique properties, stimuli-responsive polymers are being developed for use in such fields as drug delivery, cell adhesion, sensors, actuator systems, releasing of encapsulated materials and trafficking of molecules through polymeric membranes [18–23].

The “response” of a polymer can be defined in various ways. SRPs in solution are typically classified as those that change their individual chain dimensions/size, secondary structure, solubility, or the degree of intermolecular association. In most cases, the physical or chemical event that causes these responses is limited to the formation or destruction of secondary forces (hydrogen bonding, hydrophobic effects, electrostatic interactions, *etc.*), simple reactions (e.g., acid-base reactions) of moieties pendant to the polymer backbone, and/or osmotic pressure differentials that result from such phenomena. In other systems, the definition of a response can be expanded to include more dramatic alterations in the polymeric structure. In the past decade, many breakthroughs have been made in developing SPRs with novel stimulus-active mechanisms. This article reviews the mechanisms and fabrication strategies of stimulus active polymers that are sensitive to heat, light, electrical field, magnetic field, and solvent/water. The wide applications of patterned SRPs are also summarized.

## 2. Stimuli-Responsive Materials

In the context of SRPs, molecular ordering of the switching components via self-assembly is an excellent strategy. Switching primarily requires organization of individual molecules into a cooperative function, which leads to an amplification of the switching effect. Typically, each molecule contains a functional group that is responsive to stimuli, two components that create differing property-states, and a group that anchors the molecule to the surface. SRPs based on monolayers are designed by taking advantage of reversible (i) attachment-detachment of monolayer molecules [24]; (ii) conformational changes [25]; or (iii) alteration of the functional groups [26]. Below are given representative examples illustrating the various approaches to providing molecular films with stimuli-responsiveness.

### 2.1. Thermoresponsive Layers

Due to the relative ease of control, temperature is the most widely used external stimulus in synthetic and bio-inspired, stimulus-responsive systems. Many temperature-responsive polymers exhibit a critical solution temperature at which the polymer changes phase. If the polymer undergoes a phase transition from a soluble state to an insoluble state above the critical temperature, it is characterized as having a lower critical solution temperature (LCST); if the polymer transitions from an insoluble state to a soluble state with increasing temperature, it has an upper critical solution temperature (UCST) [27]. Polymers of this type undergo a thermally induced, reversible phase transition. They are soluble in a solvent (water) at low temperatures but become insoluble as the temperature rises above the LCST [28].

**Figure 1.**
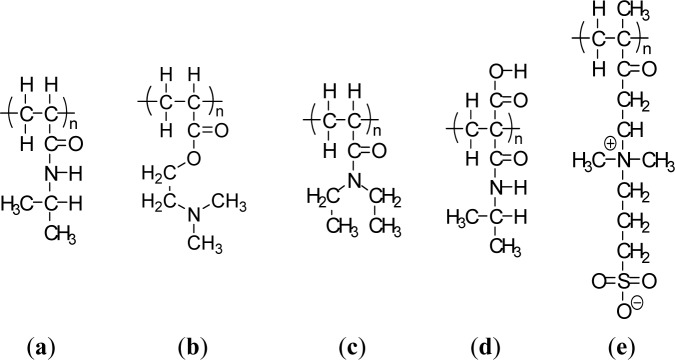
(**a**) Poly(Nisopropylacrylamide) (PNIPAAm); (**b**) poly(N,N-diethylaminoethyl methacrylate) (PDEAEMA); (**c**) poly(N,N′-diethylacrylamide) (PDEAAm); (**d**) poly(2-carboxyisopropylacrylamide) (PCIPAAm); (**e**) poly[2-(methacryloyloxy)ethyl]-dimethyl(3-sulfopropyl) ammonium hydroxide (PMEDSAH). Reprinted (adapted) with permission from [5,31–33,35].

Thermally-responsive polymers can be classified into different groups depending on the mechanism and chemistry of the groups. These are (a) poly(N-alkyl substituted acrylamides), e.g., poly(N-isopropylacrylamide) with an LCST of 32 °C [29]; and (b) poly (N-vinylalkylamides), e.g., poly(N-vinylcaprolactam), with an LCST of about 32–35 °C according to the molecular mass of the polymer [30]. Figure 1 shows respective N-substituted polyamides according to the substitution groups. A well known temperature-responsive polymer is poly(*N*-isopropylacrylamide) (PNIPAAM) (Figure 1a) with a LCST of *ca.* 32 °C, which has been widely studied for its ability to switch surface wettability [31]. This effect is explained by changes in the competition between intermolecular and intramolecular hydrogen bonding below and above the LCST. Below the LCST, the predominantly intermolecular hydrogen bonding between the PNIPAAM chains and water molecules contributes to the hydrophilicity of PNIPAAM brush films. Above the LCST, intramolecular hydrogen bonding between C=O and N–H groups in the PNIPAAM chains results in a compact and hydrophobically collapsed conformation of PNIPAAM chains, rendering the brush surface hydrophobic as well. The fundamental behavior of PNIPAAm has been extensively studied not only to understand the mechanism itself but also to develop specific technological applications. The N-isopropylacrylamide (NIPAAm) segment has been designed at the molecular level to control the LCST and the response kinetics. Poly[2-(dimethylamino)-ethyl methacrylate] (PDMAEMA) (Figure 1b) was reported to show a temperature sensitivity similar to PNIPAAm [32]. PDMAEMA is a uniquely responsive polymer, for it responds to temperature and also to pH in aqueous solution. It can also be permanently quaternized and converted to zwitterionic structures (via reaction with propanesultone), forming materials with UCST properties, demonstrating concentration-dependent thermal transformation. Another popular temperature responsive polymer is Poly(N,N′-diethylacrylamide) (PDEAAm) (Figure 1c), which has a LCST in the range of 25–35 °C [5]. Poly(2-carboxyisopropylacrylamide) (PCIPAAm) (Figure 1d) is composed of a vinyl group, an isopropylacrylamide group, and a carboxyl group, which can give two benefits: the analogous temperature responsive behavior as PNIPAAm and the additional functionality in its pendant groups [33]. PNIPAAm-co-PCIPAAm has been reported to have sensitivity and an LCST similar to those of PNIPAAm [33,34]. PNIPAAm-co-PCIPAAm is distinct from PNIPAAm-co-poly(acrylic acid) because the former has continuous isopropylacrylamide pendant groups in its chain. The continuous pendant groups do not change the temperature responsive behavior of PNIPAAm in spite of the additional carboxyl pendant groups.

Recently, an interesting UCST polymer brush, poly[2-(methacryloyloxy)ethyl]-dimethyl(3-sulfopropyl) ammonium hydroxide (PMEDSAH), was synthesized and characterized by Azzaroni *et al*. [36]. Zwitterionic PMEDSAH brushes exhibit a complex temperature behavior that depends on PMEDSAH molecular weight and results in various inter- and intra-chain associated states (Figure 1e) [35]. Another interesting class of temperature-responsive polymers that has recently emerged involves elastin like polymers (ELPs) [37]. The specific LCSTs of all these different polymeric systems show potential applications in bioengineering and biotechnology. A series of copolymers of N-acryloyl-N′-alkylpiperazine (methyl and ethyl) with polymethacrylamide(PMAAm) was investigated for their temperature and pH sensitivity [38]. Even though the homopolymers based on methylpiperazine and ethylpiperazine did not exhibit the LCST due to their weak hydrophobicity, incorporating the methacrylamide group induced an LCST for these copolymers by increasing hydrophobicity in their structures [38]. Other temperature responsive synthetic polymers showing the LCST that have been reported include poly(N-(L)-(1-hydroxymethyl) propylmethacrylamide) [p(L-HMPMAAm)] [39], poly(N-acryloyl-N′-alkylpiperazine)[40], poly(N-vinylisobutylamide) [41], poly(vinyl methyl ether) [42], poly(N-vinylcaprolactam) [43], and poly(dimethylaminoethyl methacrylate) [32]. However, these polymers have been less extensively investigated than poly(N-substituted acrylamide) (mostly PNIPAAm).

The common feature of thermoresponsive hydrogels is that hydrophobic (e.g., methyl, ethyl, propyl) and hydrophilic (e.g., amide, carboxyl) groups coexist in one macromolecular network. Although wettability depends on surface chemical functionality, surface roughness can significantly enhance the wettability response of polymer brush modified substrates [44]. For example, Sun *et al.* [44] grafted thermally responsive PNIPAAM brushes on both a flat and a rough silicon substrate via surface initiated atom transfer radical polymerization (SI-ATRP). However, reversible, thermoresponsive switching between superhydrophilic (~°) and superhydrophobic (~50°) states was realized only on microscopically rough surfaces. Similarly, Fu *et al*. [45] realized a dynamic superhydrophobic to superhydrophilic switch by synthesizing a PNIPAAM brush on a nanoporous anodic aluminum oxide surface. Luzinov *et al*. [46] reported a set of responsive surface properties allowing for capillary-driven microfluidic motion, combinatorial-like multiplexing response, reversible aggregation and dis-assembly of nanoparticles, fabrication of ultrahydrophobic coatings, and switchable mass transport across interfaces.

**Figure 2.**
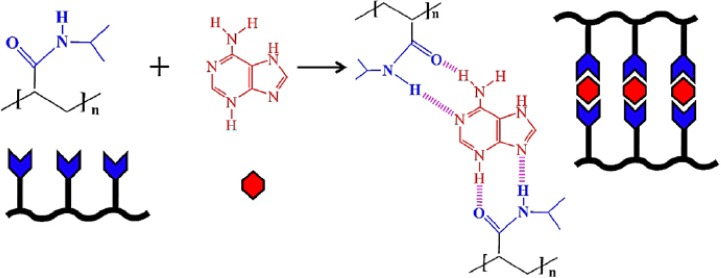
Representation of the supramolecular structure formed from the complexation of PNIPAAm and adenine through a nucleobase-like hydrogen bonding (NLHB) interactions. Reprinted (adapted) with permission from [51].

The LCST of a temperature-responsive polymer is influenced by hydrophobic or hydrophilic moieties in its molecular chains. In general, to increase the LCST of a temperature responsive polymer (e.g., PNIPAAm), it is randomly copolymerized with a small ratio of hydrophilic monomers [47]. In contrast, a small ratio of hydrophobic constituent was reported to decrease the LCST of NIPAAm as well as to increase its temperature sensitivity [48]. More-hydrophilic monomers such as acrylamide would make the LCST increase and even disappear, and more-hydrophobic monomers such as N-butyl acrylamide would cause the LCST to decrease [49]. Therefore, the LCST could be adjusted by the incorporation of hydrophobic or hydrophilic moieties. The adjustment of LCST to approximately body temperature is essential especially in the case of drug delivery applications. The influence of the LCST of NIPAAm by complexing with hydrophilic components has been investigated by varying the mole fractions between NIPAAm and complexed components [50,51]. When components such as tannic acid or adenine are complexed with NIPAAm, the LCST of NIPAAm shows a discontinuous alternation or even disappears at the pKa of the ionizable groups (Figure 2). This changeable LCST could be utilized in a targeted drug delivery system.

### 2.2. pH/Ionic-Responsive Hydrogels

The most commonly-used pH-responsive functional groups are carboxyl and pyridine groups. A carboxyl group (or carboxy) is a functional group consisting of a carbonyl and a hydroxyl, having the chemical formula –C(=O)OH, which is usually written as –COOH or –CO_2_H. At low pH, carboxyl groups are protonated and hydrophobic interactions dominate, leading to volume shrinkage of the polymer that contains the carboxyl groups. At high pH, carboxyl groups dissociate into carboxylate ions, resulting in a high charge density in the polymer, causing it to swell. In contrast to the alkali-swellable carboxyl group, pyridine is an acid-swellable group. Under acidic environmental conditions, the pyridine groups are protonated, giving rise to internal charge repulsions between neighboring protonated pyridine groups. Charge repulsion leads to an expansion in the overall dimensions of the polymer containing the groups. At higher pH values, the groups become less ionized, the charge repulsion is reduced, and the polymer–polymer interactions increase, leading to a reduction of the overall hydrodynamic diameter of the polymer [52,53].

**Figure 3.**
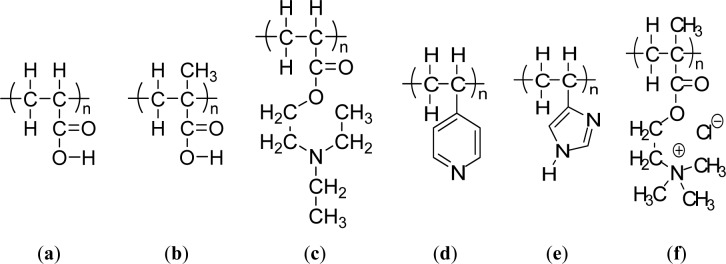
(**a**) Poly(acrylic acid) (PAAc); (**b**) poly(methacrylic acid) (PMAAc); (**c**) poly(N,N′-diethyl aminoethyl methacrylate) (PDEAEMA); (**d**) Poly(4-vinylpyridine) (P4VP); (**e**) Poly(vinyl imidazole) (PVI); (**f**) poly[2-(methacryloyloxy)- ethyltrimethylammonium chloride] (PMETAC).

Weak polyacids (or polybases), which undergo an ionization/deionization transition from pH 4~, are utilized as pH-responsive polymers. Polyacids bearing the carboxylic group with pKa’s of around 5–6 are the most representative weak polyacids. Among them, poly(acrylic acid) (PAAc) (Figure 3a) [54] and poly(methacrylic acid) (PMAAc) (Figure 3b)[55] have been most frequently reported as pH responsive polyacids. Their carboxylic pendant groups accept protons at low pH, while releasing them at high pH. Therefore, they are transformed into polyelectrolytes at high pH with electrostatic repulsion forces between the molecular chains. This gives a momentum, along with the hydrophobic interaction, to govern the precipitation/solubilization of molecular chains, deswelling/swelling of hydrogels, or hydrophobic/hydrophilic characteristics of surfaces. PMAAc shows a phase transition that is abrupt in comparison to the relatively continuous phase transition of PAAc. PMAAc has a compact conformation before a critical charge density is attained because the methyl groups in PMAAc induce the stronger hydrophobic interaction as the aggregation force. Introducing a more hydrophobic moiety can offer a more compact conformation in the uncharged state and a more dramatically discontinuous phase. Following this, poly(2-ethyl acrylic acid) (PEAAc) and poly(2-propyl acrylic acid) (PPAAc) contain more hydrophobic properties, which provide a more compact conformational structure at low pH [56,57]. Poly(N,N′-dimethyl aminoethyl methacrylate) (PDMAEMA) (Figure 1b) and poly(N,N′-diethyl aminoethyl methacrylate) (PDEAEMA) (Figure 3c) are examples of pH responsive polybases. The amine groups are located in their side chains. The amine groups gain protons under acidic condition and release them under basic condition. In PDEAEMA, the longer hydrophobic groups are at the end of the amine group, which causes stronger hydrophobic interactions at high pH, also leading to “hypercoiled” conformations. PDEAEMA homopolymer undergoes an abrupt precipitation above pH 7.5 due to the deprotonation of amino groups, followed by hydrophobic molecular interactions [58]. Another widely-used polymer-containing pyridine is poly(vinyl pyridine), which is based on basic monomers, such as 4-vinylpyridine (4VP) or 2-vinylpyridine (2VP). The pKa of poly(vinyl pyridine) in solution is approximately 3.5–4.5, depending on the measurement method and its form [59–61]. Poly(4 or 2-vinylpyridine) (P4VP or P2VP) show pH-sensitivity (Figure 3d). These polymers undergo a phase transition under pH 5 owing to deprotonation of pyridine groups [62]. Poly(vinyl imidazole) (PVI) is another pH responsive polybase bearing the imidazole group (Figure 3e) [63]. Quaternized poly(propylene imine) dendrimers have been investigated as pH-responsive controlled-release systems [64]. Other pH-sensitive functional groups, such as imidazole, dibuthylamine, and tertiary amine methacrylates have also been investigated [65]. These groups are also cationic groups and are acid-swellable.

The way to achieve the water solubility of poly-(sulfobetaine) is to add a simple salt. The site-binding ability of the cation and the anion allows polymer chains preferentially to complex the low molecular weight electrolyte and reduce the attractive inter-chain interaction, leading to chain expansion in aqueous solution. For cationic polyelectrolyte brushes, however, adding a salt was found to screen the electrostatic interaction within the polyelectrolyte, resulting in conformation changes from stretched to collapsed form. Indeed, Mueller *et al.* [66] found that the quaternized PMAEMA (PMETAI) brushes collapse in solution at a high concentration of monovalent salt. Another example is poly[2-(methacryloyloxy)- ethyltrimethylammonium chloride] (PMETAC) (Figure 3f) brushes that contain Cl^−^ counterions [67]. Replacing the Cl^−^ anions with SCN^−^, PO_4_^3−^ and ClO_4_^−^ anions promotes a drastic change in the wetting properties of the substrate. The interaction between the quaternary ammonium groups in the brushes and the surrounding counterions thus plays a major role in determining surface wettability.

### 2.3. Photo-Responsive Film

**Figure 4.**
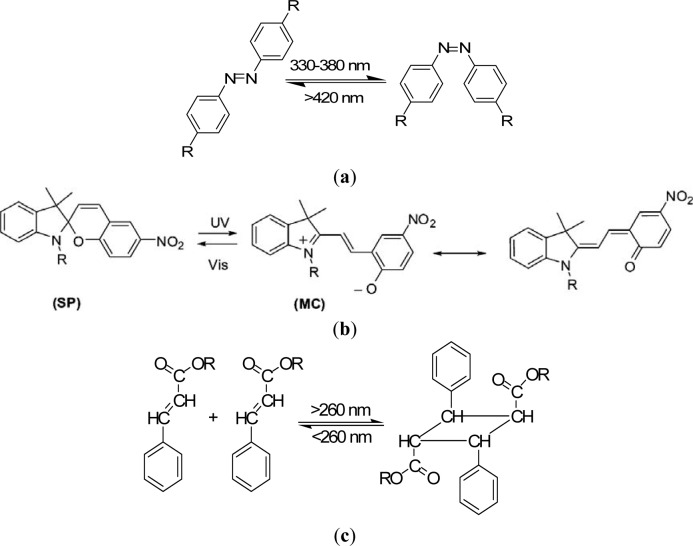
Schematic illustrations of reversible photoisomerization. (**a**) photoisomerization of azobenzene groups; (**b**) isomeric molecular structure of a spiropyran irradiated with light, spiropyran (left), merocyanine (center), and quinoidal canonical form (right); (**c**) photodimerization of the cinnamic acid group. Reprinted (adapted) with permission from [74,78,82].

The design principles for stimuli-sensitive polymers are elucidated exemplarily for photosensitive polymers. Rhodopsin, a sensory molecule in visual perception, is an example of a photosensitive polymer from nature. The use of light as an external trigger is particularly interesting because it entails several advantages, such as scalable miniaturization, limited chemical contamination, and ease of operation [68]. Photodeformable polymers are mostly based on the following mechanisms: (a) photoisomerizable molecules such as azobenzenes [69]; (b) photoreactive molecules such as cinnamates [70]; (c) addition-fragmentation chain transfer reaction using allyl sulfides [71]; and (d) reversible photoinduced ionic dissociation such as trienphylmethane leuco. Complicated movements such as oscillating, twisting, swimming [72], rotation, and inchworm walk have been obtained on photoactive polymer films and laminated films [73]. As shown in Figure 4a, upon exposure to light of an appropriate wavelength, azobenzenes show reversible *trans-cis* isomerization. The trans-cis isomerization leads to a change in the angle between the two aromatic rings, with the distance between the 4- and 40-carbons falling from 9.0 Å (*trans*) to 5.5 Å (*cis*) [74]. Azobenzene units exhibit a photo-switching effect by undergoing a *trans*- to *cis*-isomerization which corresponds to different dipole moments. Furthermore, these molecular changes in the dipole moment translate into alteration of surface wettability [75]. When azobenzene groups are linked to macromolecules, the interconversion between the two photoisomers can induce macroscopic changes in the polymeric material. The photosensitive groups allow for photo-controllable self-assembly and self-organization of block copolymers as well as low molecular weight gelator molecules in solution, switch assemblies at surfaces, and photo-induced swelling and shrinkage of gels, e.g., functionalized poly(N-isopropylacrylamide).

Azobenzene derivatives have been incorporated into peptides to alter their structural arrangements via isomerization [76]. Like azobenzenes, spiropyrans are photoresponsive molecules that can reversibly switch between a closed nonpolar form and an open polar form upon photochemical cleavage of the C–O in their ring in the presence of UV light [77]. Locklin *et al.* [78] describe a method for the formation of photochromic poly(spiropyran methacrylate-co-methyl methacrylate) (PSPMA) brushes grafted from oxide surfaces. The light-induced conformational changes result in reversible side-chain cleavage of the spiro C–O bond, allowing for the switching between a colorless closed spiropyran and a colored open merocyanine form. The relatively nonpolar spiropyran can be switched to a polar, zwitterionic merocyanine isomer using light of an appropriate wavelength (Figure 4b). These polymer brushes are ideal because of both the large change in dipole moment between the two isomeric states and the excellent stability of the chromophore to cycling between UV and visible light. After irradiation with 365 nm light, the surface energy increases with a concomitant decrease in the water contact angle, and upon subsequent irradiation with visible light, the surface recovers its initial hydrophobicity.

Therefore, coating a surface with, and/or incorporating spiropyran molecules into, a substrate allows for controllable wettability [79]. Rosario *et al.* [80] demonstrated this concept by coating glass capillaries with photoresponsive spiropyran molecules and observing a rise in the water level as a result of UV light. More recently, Athanassiou *et al.* [81] combined the wettability control of spiropyrans with nanopatterning. In this work, light-controlled volumetric changes (as a result of irradiation cycles of UV and green laser pulses) were assessed on nanopatterned poly(ethyl methacrylate)-co-poly(methyl acrylate), P(EMA)-co-P(MA) doped with spiropyran. Once patterned via soft lithography and exposed to UV irradiation, the doped P(EMA)-co-P(MA) exhibited hydrophilic surface properties, as indicated by a reduction of the contact angle. Illumination of the sample with green laser pulses returned the surface to a hydrophobic state [80]. This change in wettability was attributed to dimensional changes in the nanopattern as a result of light irradiation.

Photochemically reactive molecules are also able to form photoreversible covalent cross-links in polymers. The reversible photodimerization of the cinnamic acid group is shown in Figure 4c [82]. The film is first stretched and irritated by UV light longer than 260 nm. The photoinduced cycloaddition reaction of the cinnamic acid groups increases the elastic modulus of the polymer, which induces the fixity of the polymer film. Then the film is released and recovers partially as a result of instant elasticity. Finally, irradiation of the deformed sample with UV light shorter than 260 nm causes the photocleaving of cinnamic acid groups and decreases the elastic modulus, leading to the shape recovery of the polymer. The design and synthesis of novel photosensitive molecules is a challenging area for future research. As the existing molecules require UV or visible light, the development of molecules sensitive to the NIR range would be desirable, especially for biomedical applications, where deep light penetration without harming tissue is required.

**Figure 5.**
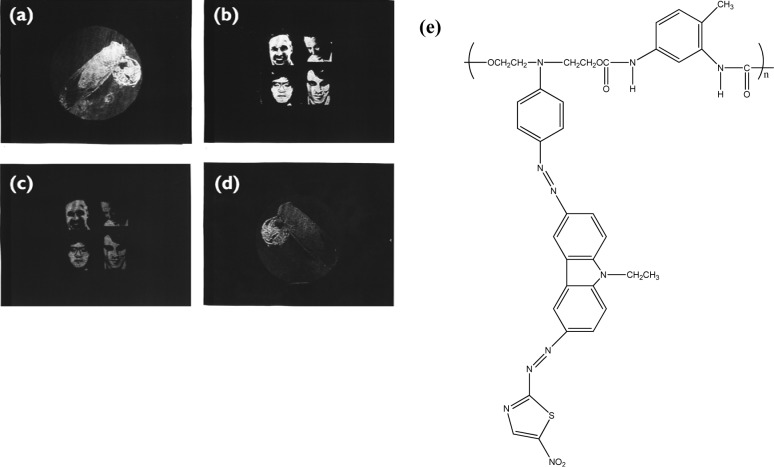
(**a**) Reconstructed image of a cicada (a familiar insect in Taiwan) standing on the cross section of a branch written in the PANPAC/TDI film; (**b**) overwritten image of four portraits; (**c**) dark decay after 1 h; (**d**) same image as in part a written in the PANTAC/TDI film; (**e**) chemical structure of the polymer. Reprinted (adapted) with permission from [84]. Copyright (1999) American Chemical Society.

Holographic data storage is a promising storage technology that can provide both a high storage density and a fast readout rate. Photorefractive polymeric materials have attracted much attention in recent years. Photoconducting nonlinear optical polymeric films that exhibit photorefractive properties can be used for holographic image storage. For the polymer composites system, wherein all functional entities are doped into the polymer PR composites, the practical application of photorefractive devices is limited because of phase separation and diffusion-induced stability problems. These problems can be avoided in fully-functionalized photorefractive polymers because all the functional entities for the photorefractive effect are covalently bonded to the polymers, affording stable photorefractive properties [83]. To enhance the photorefractive performance of the polymers, dual functional carbazole-based chromophores are synthesized by attaching the electron-donating (N,N-diethanol aminophenyl) and electron-accepting groups (*p*-nitrophenyl or 5-nitrothiazole) with a diazo bridge on the 3- and 6-positions of the N-ethylcarbazole [84]. The chromophore exhibited a large first hyperpolarizability in a hyper-Rayleigh scattering experiment due to the extended chain length. PANPAC/TDI is a good hologram recording media. The images can be stored, erased, and updated repeatedly. The contrast and the brightness of the recorded holograms are greatly enhanced (Figure 5).

For the 3-amino-9-ethyl carbazole/Dispersed Orange 3(DO3)/diglycidyl 1,2 cyclohexanedicarboxylate main chain copolymers, the grating growth rate can be accelerated by incorporating the sensitizer or increasing the charge transfer component concentration [85]. The dark decay of the PR properties at elevated temperature can also be evaluated by the thermally-stimulated discharge current spectroscopy technique. The recorded pattern exhibits good fringe contrast, with a resolution of 20 μm in the recorded hologram [86]. The formation rate of the refractive index grating (PR grating) can be accelerated by applying an electric field or changing the comonomer structure or monomer composition. Faster PR response can be achieved by incorporating aliphatic diepoxy with a longer chain length or by increasing the concentration of the charge transport moieties, while the more nonlinear optical (NLO) segment (DO3) in the copolymer results in higher diffraction efficiency. When the charge transport dopant was incorporated into the nonlinear optical polymer PMDA-DR19 polymer film, it became photoconductive and photorefractive [87]. Films containing more nonlinear optical chromophores exhibited higher diffraction efficiency. Response speed was further elevated when an additional charge generation material was doped in. In addition to composition modification, the laser light source was also an important factor in changing the PR response speed. The photorefractive response speed was tremendously improved when films were written and read by two different laser beams, either red/green lasers or green/red lasers. A thermal stimulated current (TSC) spectrometer can be used to study the trap characteristics of photorefractive polymeric materials [88]. In addition to the normal positive peaks due to segmental disorientation induced polarization, negative peaks were also observed, probably due to the release of trapped field-induced positive carriers during polarization. The negative peak distinguished the trap characteristics from segmental disorientation behavior. TSC is a useful tool for studying the trap characteristics of photorefractive polymers containing organic conducting material.

### 2.4. Magnetically-Responsive Composites

Magnetically-active polymers respond to changes in magnetic fields. Also called magnetoelastic or magnetostrictive polymeric composites, they are composites of elastomers or gels filled with small magnetic particles. Magnetically-active polymeric composites (MAPCs) with tailor-made anisotropic particles can be prepared under external fields. Also called ferrogels, MAPCs are swollen networks filled with super-paramagnetic particles. The typical fillers used to achieve the MAPCs include metal particles, iron(III) oxide particles [89], ferromagnetic particles [90], NdFeB particles [91,92] and nickel powders [93]. Because these materials were originally proposed for biomedical applications, the SMP matrixes are mostly biodegradable and biocompatible polymers such as poly(D,L-lactide), cross-linking poly(ε-caprolactone), poly(*p*-dioxanone)- poly(ε-caprolactone) copolymer, cross-linking oligo (ε-caprolactone) dimethacrylate/butyl acrylate, and grafting polymer poly(ε-caprolactone) diisocyanatoethyl methacrylate (PCLDIMA) and poly(ethylene glycol) mono-methylether-monomethacrylate (PEGMA). Based on the polymer matrix used, MAPCs are categorized into magnetically-active elastomers and magnetically-active polymeric gels.

Magnetically-active elastomers are composites of high elastic polymeric elastomers filled with small nano- or micron-sized magnetic particles. When a magnetic field is applied to the composite, all forces acting on the magnetic particles are transmitted to the elastomer, changing the shape of the elastomer [94]. If the field is non-uniform, the magnetic particles experience a magnetophoretic force. Consequently, the particles are attracted to regions of stronger field intensity, which induce the macroscopic shape distortion of the magnetically-active elastomer [95]. To obtain high magnetic sensitivity, the elastomer needs to have a low elastic modulus and high initial susceptibility as well as high saturation magnetization. The filler particles can be from ferri- and ferromagnetic materials. In addition, grafted polymers, long used to stabilize nanoparticle suspensions, utilize the sterically repulsive interactions of long polymer chains. The use of stimulus-responsive polymer brushes could bestow additional functionality to these nanoparticle systems. For example, Benkoski *et al.* [96] reported the magnetic self-assembly of dispersed ferromagnetic PS-coated cobalt nanoparticles (PS-CoNPs) into 1D microsized polymer chains at the interface of water and an organic solvent. Although the use of PS-coated nanoparticles was originally predicated on imparting colloidal stabilization, the selective solubility of the PS coating provides an equally beneficial localization to the interface of immiscible fluids.

**Figure 6.**
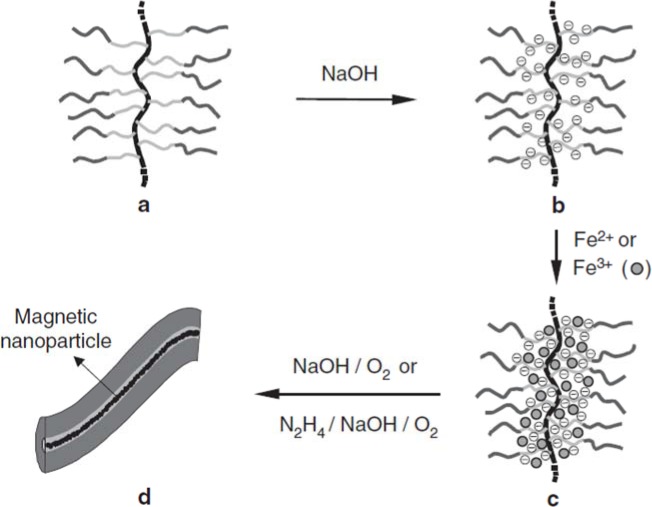
Schematic illustration for the synthesis of a wire-like assembly of magnetic nanoparticles inside a cylindrical polymer brush. (**a**) polymer brush with PAA core and PnBA shell; (**b**) neutralized polymer brush with poly(sodium acrylate) core (Na^+^ ions are not shown); (**c**) polychelate of the brush with Fe^2+^ or Fe^3+^ ions; and (**d**) hybrid nanocylinder of the brush and magnetic nanoparticles. Reprinted (adapted) with permission from [10].

Magnetically-active polymeric gel can also be regarded as a chemically cross-linked polymer swollen by a ferrofluid. Ferrofluid is a colloidal dispersion of monodomain magnetic particles with a typical size of *ca.* 10 nm. In magnetically-active polymeric gel, the finely distributed ferromagnetic particles are attached to the flexible network by adhesive forces, resulting in direct coupling between the magnetic and mechanical properties of the magnetically-active polymeric gel [97]. Moreover, gels sensitive to magnetic fields were obtained by incorporating colloidal magnetic particles into poly(N-isopropylacrylamide-co-N,N-dimethylacrylamide) [98]. The gel beads formed straight chainlike structures in uniform magnetic fields, while they aggregated in nonhomogeneous fields. The rapid and controllable shape changes of these gels would be expected to mimic muscular contraction. In addition, hydrogels with block copolymer side chains can act as templates for the preparation of nanostructured hybrid materials by coordination with metal ions. Various metals and metal oxides, semiconducting nanoparticles have been prepared in this way. Similarly, as shown in Figure 6, cylindrical brushes with diblock copolymer side chains composed of PAA-b-PBA have been used as single molecular templates for the preparation of magnetic nanoparticles, wherein PAA core blocks coordinate with Fe^2+^ or Fe^3+^ ions and the PBA shell to provide stability to the nanoparticles [10]. The temperature-dependent magnetic properties of the hybrid nanocylinders have been investigated. In the temperature range of 25–295 K, the fabricated nanoparticles are superparamagnetic, for no hysteresis is observed. The ferrimagnetic nature of the fabricated magnetic nanoparticles, however, was detected at very low temperatures such as 2K, where the hysteresis loop was symmetric.

Magnetically-active composites can be used for bearings and vibration absorbance, drug targeting, automotive bushings, magnetic tapes, magnetic gums, soft actuators, micromanipulators, artificial muscles, and suspension devices.

### 2.5. Electroactive Composites (EACs)

One of the major accomplishments of EACs in the past decade is electroactivity using a low voltage. A certain level of electrical conductivity can be reached if the EACs are filled with enough electrically-conductive ingredients. The conductive fillers can also improve the thermal conductivity of EACs, which contributes to a fast response. EACs fall into two major categories based on their primary activation mechanisms: electronic electroactive polymers (driven by electric field and coulomb forces) and ionic electroactive polymers (driven by the movement of ions) [99]. The conductive fillers that have been used include carbon nanotubes (CNTs), polypyrrole, carbon black (CB), Ni powders, Ni nanorods, short carbon fibers, and CNF mats. Such electricity-triggered EACs are especially useful for applications where direct heating is not possible, such as in self-deployable aerospace structures, implanted biomedical devices, actuators, and sensors.

**Figure 7.**
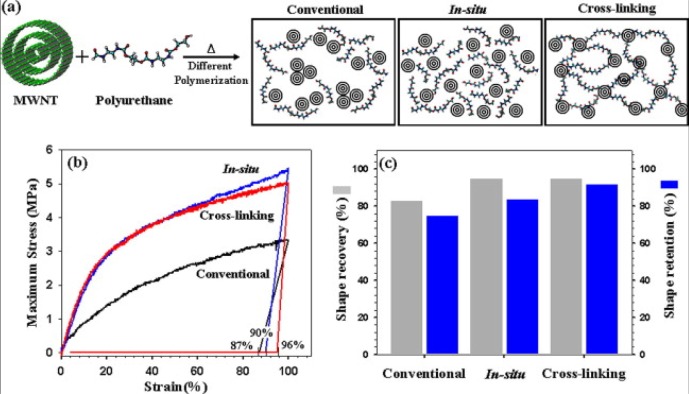
(**a**) A schematic representation of the controlled dispersed structures of the tubes within the polymer matrix; (**b**) thermomechanical properties curves (200% elongation at 50 °C); and (**c**) shape recovery and shape retention (quenching/unloading and subsequently applying an electric voltage of 40 V) for the polyurethane composites prepared by conventional blending, *in situ*, and cross-linking polymerization. Reproduced from Reference [104] with permission.

A large amount of CB fillers, which are not as effective at improving the electrical conductivity as high aspect ratio fillers, can be filled into EACs up to 40 wt%. Le *et al.* [100] studied an electroactive EAC composed of CB-filled highly branched ethylene-1-octene copolymer. It was found that increasing the mixing time could increase the electroactivity of the EAC because it could make the distribution of the CBs more homogenous. Short carbon fibers and CBs have a good synergic effect on improving the electrical conductivity of the composite, with short carbon fibers acting as a conductive bridge to connect the CBs. Leng *et al.* [101] demonstrated that the CBs and short carbon fibers helped each other, significantly improving the electrical conductivity. Cho *et al.* [102] first reported electroactive EACs composed of polymers filled with CNTs through joule heating. The CNTs were first treated with acid, and surface modification of the CNTs decreased the electrical conductivity of the composites because the acid treatment increased defects in the lattice structure of the C–C bonds. Heat treatment of CNT/polymer composites can remarkably improve the conductivity of composites, as reported by Fei *et al.* [103]. They prepared CNTs/poly(methylmethacrylateco-butyl acrylate) by ultrasound assisted in-situ polymerization. The composites were subjected to a simple heating and cooling process. The mechanism of the increased electrical conductivity was tentatively described as follows: In the original composite, there may be some internal residual stresses or strains in the interface between the polymer and CNTs as a result of thermal expansion mismatch and curing shrinkage. After the post thermal treatment, the interface contact area between the polymer matrix and CNTs may increase, and the thickness of the interfacial polymer may decrease. The two effects reduce the tunneling resistance and thus significantly enhance the electrical conductivity. Covalent linkages between CNTs and the polymers can effectively prevent the re-aggregation of the CNTs within the polymer matrix. To achieve the cross-linking between CNTs and EACs, Jung *et al.* [104] modified CNTs using acid so that the CNTs could act as a cross-linking agent during the in-situ polymerization step of the polymer. The –OH groups of the acid-treated CNTs and the –NCO groups of the icosyanate-terminated prepolymer form cross-linking structures, as shown in Figure 7. The prepared EAC showed outstanding shape memory properties and mechanical properties.

Zhang *et al.* [105] fabricated an all-organic electroactive composite using a ferroelectric electroactive polymer P(VDF-TrFE) as the matrix and copper phthalocyanine (CuPc) as the filler. The high dielectric constant of CuPc is due to the easy displacement of the electrons under an electric field within the entire molecule. Researchers [106] also combined CuPc and conducting polyanline within polyurethane matrix to prepare all-organic electroactive materials. The high-dielectric-constant CuPc particulates enhanced the dielectric constant of the polyurethane matrix, and this combination of the two-component dielectric matrix in turn served as the high-dielectric-constant host for the polyanline. Huang *et al.* [106,107] fabricated an all-polymer high-dielectric-constant percolative material by combining polyaniline particles within a fluoroterpolymer matrix. The polyaniline was coated with an insulating polymer having high compatibility with the matrix in order to improve the breakdown field.

### 2.6. Solvent-Responsive Composites

**Figure 8.**
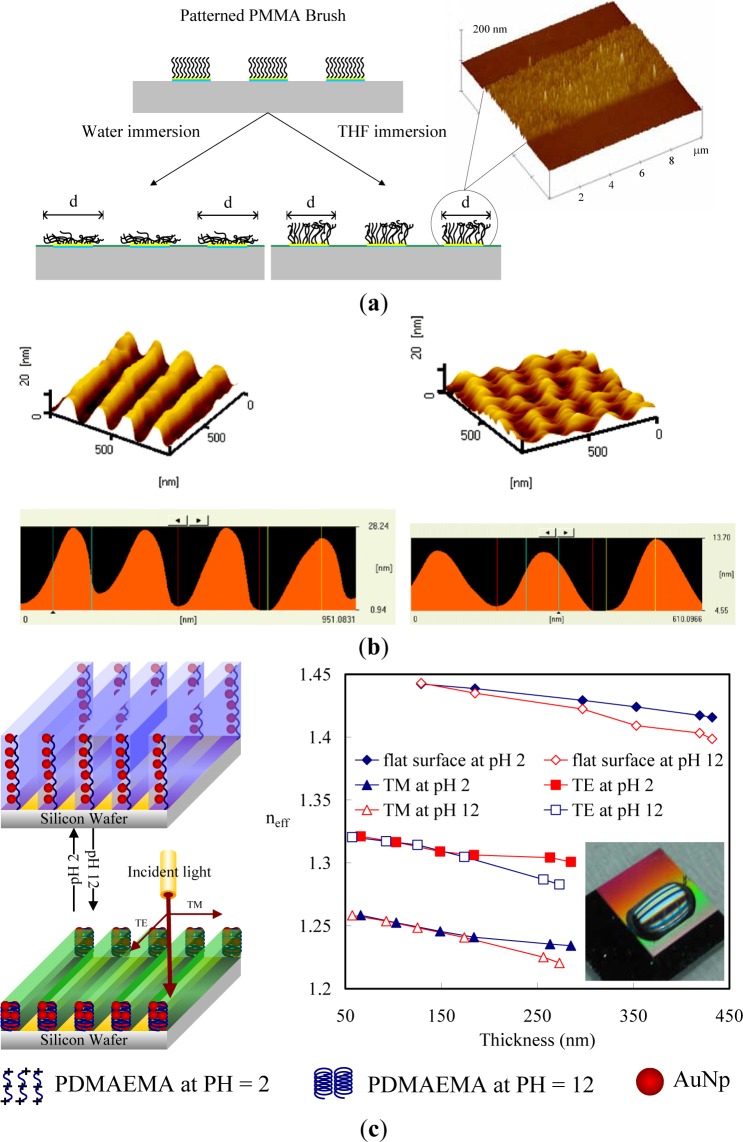
(**a**) Schematic illustrations of reversible poly(methyl methacrylate) (PMMA) brush treated with good and poor solvents; (**b**) Atomic force microscopy (AFM) images of densely patterned lines polystyrene (PS) brushes (1:1 duty ratio, 160 nm resolution) before and after good (**left**) and poor (**right**) solvent treatments, respectively; (**c**) Schematic of a polymeric sensor based on a surface-grafted polymer layer with AuNPs, adsorbed on a stimuli-responsive polymer line pattern grafted onto a silicon substrate. The AuNPs-surface distance depends on the conformation of the polymer chains and changes in different solvents. The change in height is therefore reported by a variation in the detected diffractive intensity. Reprinted from Reference [108,110,114] with permission.

Solvent-active composites (SACs) can be obtained on deformed polymers because solvent molecules cause swelling on polymeric materials and increase the flexibility of the macromolecule chains of the polymers. Switching of the conformation of surface-grafted polymer chains is one approach to designing surfaces with switchable properties. The single molecular conformation transition of brushes by solvent treatments involves the preparation of patterned molecular brushes. Along this line, Chen *et al.* [108,109] grafted PMMA brushes with line patterns to observe the line pattern deformation (Figure 8a). PMMA brushes are interesting because their line scales can be varied by good and poor solvent treatments. Due to the responsiveness of polymers toward different kinds of solvent, conformational transitions were observed as a function of solvent quality. To obtain more specific conformation transitions in the brushes, Chen *et al.* [110,111] synthesized PS brushes within nanoscale line patterns and studied solvent-induced variation of the molecular brushes. They found that the line scale of brushes varied with the solvent quality. Furthermore, the deformation of PS brush lines was evaluated by AFM with the friction force. They predicted switching between the morphologies of pinned micelles and a swollen brush layer by varying the solvent quality. In good solvent conditions, polymer chains are swollen and completely screen the substrate. In poor solvent conditions, however, polymer chains try to avoid unfavorable contact with the solvent and form compact micelles, leaving the substrate partially accessible to the environment. Significant changes in the friction forces of the surfaces possessing line patterns of PS brushes were observed (Figure 8b). Thereby, the grafted polymer chains can either completely or partially cover the substrate, which can be used in the design of switchable surfaces.

Furthermore, several research groups have explored the reversible swelling of surface grafted polymer chains in response to changes in environmental conditions as a means to control the vertical and lateral motion of micro- and nanoparticles. For example, homopolymer [112] and diblock-copolymer [113] brushes were used to move adsorbed metal or semiconductor nanoparticles in the vertical direction by switching the conformation of polymer chains. Here, the advantage of diblock-copolymer brushes is the possibility of freezing the vertical position of nanoparticles by drying. Depending on the interactions of the solvent with the polymer block to which nanoparticles are linked, the nanoparticles can be either positioned on the surface or hidden in the vicinity of a substrate. In many cases, the change of the conformation of polymer chains causes changes in the interactions between the attached particles, including a plasmonic effect, fluorescence quenching, or light interference, that can be used to design environmental [114] and biochemical [115] sensors. One possibility is the incorporation of gold nanoparticles (AuNPs) into polymer layers grafted onto reflecting surfaces (Figure 8c). Switching the conformation of polymer chains results in changes in the distance between the AuNPs and the substrates. As a result, the interference between the light directly emitted by AuNPs and the light reflected by the grating surface changes. Consequently, the intensity of the detected diffractive light depends on the conformation of grafted polymer chains. The main advantages of this approach are extreme instrumental simplicity, high spatial precision of the measurements, and fast signal response. Minko *et al.* [116] have used this principle to design composite surfaces with adaptive adhesion, prepared by grafting poly-(ethylene glycol) (PEG) chains between fluorinated particles; PEG and fluorinated particles are non-sticky in aqueous and dry environments, respectively. It was shown experimentally that PEG chains are collapsed in air and are hidden under fluorinated particles, which makes the composite surface non-adhesive in the dry state. On the other hand, in an aqueous environment, non-sticky PEG chains swell and screen sticky particles. It makes the surface non-adhesive in an aqueous environment.

Unlike the conformation transition of brushes in solution, solid state conformation changes driven by exposure to different kinds of solvent are particularly interesting due to the possibility of both *in situ* visualization by AFM and manipulation of the conformational properties of molecular brushes. Moeller and co-workers studied the conformational transitions of PBPEM-g-PBA brushes induced by cyclic exposure of the wafers with the adsorbed brushes to water and alcohol vapors [117]. Exposure to saturated alcohol vapor caused the adsorbed individual polymer chains to collapse, while exposure to saturated water vapor caused them to extend [118]. In the presence of an alcohol layer with a lower surface energy, these brushes tend to minimize the occupied surface area through partial desorption of the PBA side chains, leading to a transition from an extended to a globular conformation. Upon condensation of water vapor, PBA side chains readsorb to the substrate and cause the backbone to extend. Polymer chain statistics and scaling exponents that describe the correlation between the mean square end-to-end distances and the contour lengths of macromolecules have been determined by real-time AFM. Thus, manipulating and imaging single molecular brushes *in situ* and in real time on a silicon substrate opens up new possibilities for the controlled structure formation in ultrathin polymer films.

## 3. Stimuli-Responsive Hydrogels Patterning

During the last two decades, many techniques have been developed to selectively position organic molecules and then to obtain well-defined patterned substrates at the micrometer or submicrometer scale. They are divided into several categories, such as photolithography, direct writing techniques, printing techniques, and particle beam lithography. The development of pattern techniques has three directions: (1) complication; (2) smaller pattern; and (3) functionalization. The complication of patterns includes the fabrication of patterns with more complex structures, multi-scaled patterns, and multi-component patterns. The top-down methods, such as lithography, are mature techniques for producing micro- and even nano-patterns with ordered structures. The more complex the pattern generated by the top-down method is, the more novel and complex the optical pattern will be.

Reducing the pattern size down to the nanometer scale is one of the most important aspects of pattern applications, and it requires new techniques or new materials. Nowadays, techniques such as e-beam lithography and dip pen lithography are capable of generating nano-scaled chemical and topological patterns on the silicon wafer, which can then be used to guide the dewetting into ordered structures. However, these nano-patterns do not seem to be adequate for the generation of ordered nano-scaled structures. Patterns with dimensional sizes down to sub-100 nm have been achieved on a chemically patterned substrate using specific materials, such as block copolymers. However, an ideal copy of the substrate pattern can be achieved only under narrowly-constrained conditions.

### 3.1. Photolithography

Lithography can be achieved with several kinds of irradiation, including UV-Vis light and X-Ray, electron, and ion beams. These tools are able to generate patterns with a resolution varying from micrometers to 100 nm. One of the major advantages of lithographic techniques for generating patterns is that the resolution is determined by the size of the beam applied to the SAMs. However, the cost of the equipment and infrastructure required is relatively high. Many studies have been devoted to patterning via these lithographic techniques using several types of irradiation. Two important methods, described below, are photolithography and electron beam lithography.

**Figure 9.**
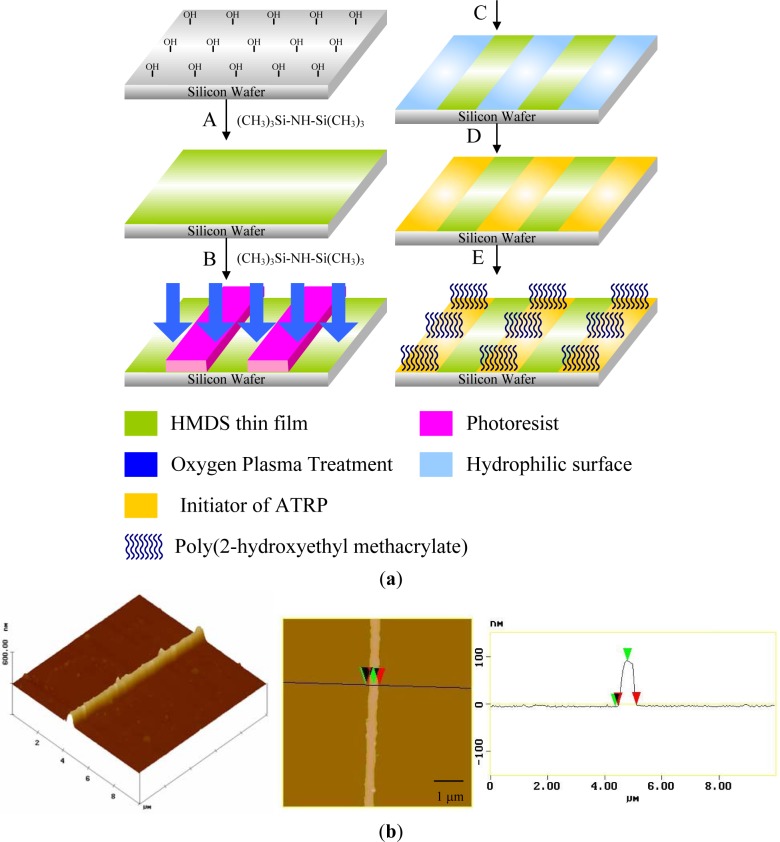
(a) Synthetic route toward poly(2-hydroxyethyl methacrylate) (PHEMA) brushes patterned through advanced lithography, and ATRP on Si wafers; (b) 3D, cross-section, and profile AFM image of line patterned PHEMA brushes with 350 nm of resolution. Reprinted from Reference [119,120] with permission.

Photolithography consists of transferring geometric shapes on a mask to a metallic surface such as silicon wafer. Photolithographic patterning can be achieved either on SAMs or on photoresistive materials, which can be defined as an organic polymer sensitive to ultraviolet light. Based on this strategy, Chen *et al.* [119] synthesized well defined poly(2-hydroxyethyl methacrylate) (PHEMA) brushes by SI-ATRP from a patterned ATRP initiator obtained from the UV illumination of a homogeneous monolayer through a photo mask (Figure 9a). The silicon wafer was previously treated with hexamethyldisilazane to form an inert monolayer. The oxygen plasma led to the decomposition of the inert monolayer in the exposed areas using positive photoresist as a hard mask. Subsequently, 11-(2-bromo-2-methyl)propionyloxyundecyltrichlorosilane was formed on the exposed areas to act as patterned initiators for ATRP. SI-ATRP of HEMA took place only on areas that possessed initiators to obtain a high aspect ratio (~.3) of PHEMA line patterns [120] (Figure 9b). Recently, Mathieu *et al.* [121] demonstrated the possibility of patterning a homogeneous octadecylsiloxane monolayer with a focused beam of an Ar laser at λ = 514 nm. The methyl end-groups of the stripes were aminated and subsequently coupled with α-bromoisobutyryl bromide in order to trigger the polymerization of NIPAAm. The lateral dimensions of polymer structures ranged from several micrometers down to the sub-100-nm range. Prucker *et al*. [122] immobilized an azo initiator on silicon substrate and placed a TEM grid with quadratic holes in contact with the monolayer. The photopolymerization of styrene took place in the illuminated areas. The sample was then carefully washed in order to remove any non-bonded polymer.

Another approach to patterning polymer brushes was described by Husemann *et al*. [123]. Initially, poly(tertbutyl acrylate) brushes were synthesized from a layer of initiating groups on silicon surface. Subsequently, a solution of polystyrene containing bis(tertbutylphenyl) iodonium triflate (8 wt% wrt PSt) was spin-coated onto the top of the brush layer to give a 1-μm-thick sacrificial photoresist layer. The surface was illuminated (λ = 248 nm) through a mask, resulting in the photo generation of acid in specific areas of the polystyrene over layer. At elevated temperature, photo generated acid diffused into the polymer brush layer and deprotected the tert-butyl ester groups to create poly(acrylic acid) chains. The sacrificial photoresist layer was then removed by simple washing with an appropriate solvent. In this case, the authors demonstrated a way to produce binary patterned brushes, which are developed extensively later in this document. On the other hand, Fan *et al.* [124] combined for the first time SIATRP and molecular assembly patterning by lift-off (MAPL) techniques in order to create micropatterning of grafted polymer. A photoresist was spin-cast and illuminated through a mask by UV. After the development step, ATRP initiator was immobilized between the circular domains of photoresist. After removal of the physisorbed layer, SIATRP was performed from chemically adsorbed initiator in order to polymerize methyl methacrylate macromonomers with oligo(ethylene glycol) (OEG) side chains. The authors demonstrated the feasibility of this strategy to produce a surface with cell-adhesive and cell-resistant regions. Zhou *et al.* [125] exploited another strategy, which involved covering the entire gold surface with poly(hydroxyethyl methyacrylate) brushes synthesized by a grafting-from technique. Subsequently, the polymer layer was passivated by a reaction with NaN_3_ and etched with UV irradiation through a TEM grid. After this treatment, the organic layer (initiator and polymer brush) on the exposed area was completely removed. In this case, the micropatterning of grafted polymer was realized directly on the polymer and not on the initiator layer.

### 3.2. Electron Beam Lithography

**Figure 10.**
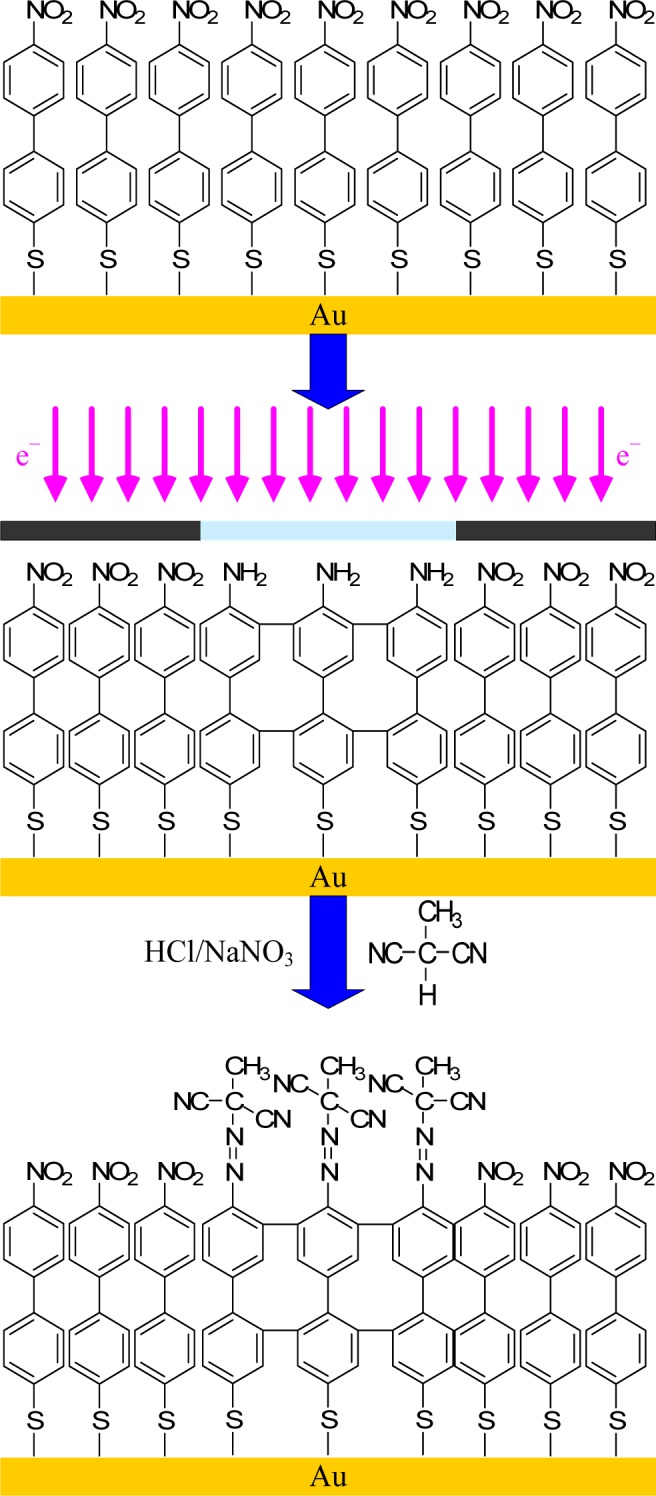
Irradiation through a mask, conversion of the terminal nitro group in amine group and diazotization and coupling with malonodinitrile gives a SAM that bears an asymmetric azo-initiator. Reprinted from Reference [128] with permission.

Electron beam lithography (EBL) was developed soon after the development of scanning electron microscopy in 1955 and arose as an attractive alternative to fabricating nanostructures by X-Ray and UV-lithography. Electron lithography offers higher patterning resolution because the electron beam can be readily focused to a diameter of approximately 1 nm. Electron beam lithography is intensively used in both resist-based and chemical approaches. As far as the chemical approach is concerned, electron irradiation has been applied to a variety of different SAMs. Some studies have shown the possibility of inducing the cleavage of C–S bonds, but also some side-reactions, such as desorption of H_2_, cleavage of the C–H bonds in methyl and methylene entities, cross-linking, and the formation of C=C bonds [126]. A patterned film of acrylic resin upon controlled EBL yielded positive and negative resists for the first time by the combination of an electron beam and controlled polymerization [127]. The acrylic resin derivative was bombarded by the electron beam, which decomposed and restructured the molecules according to the dosage. Schmelmer and co-workers have chemically modified a more exotic SAM to produce an azo-polymer initiator. Consequently, brush patterns with lateral resolution approaching 70 nm have been produced by the conversion of nitro-based SAMs into amine functionalities [128] (Figure 10). This was followed by diazotization and coupling with malonodinitrile. The monolayer of 4′-azomethylmalonodinitrile- 1,1′-biphenyl-4-thiol (cAMBT) was finally exposed to a styrene solution and synthesized through radical polymerization for 6 h in toluene at 80 °C. Moreover, a defined grafting-density gradient was prepared and controlled by electron-beam chemical lithography (EBCL). Structures with fine control of the shape, size, position, and thickness of PNIPAAm patterns were elaborated by He *et al.* [129]. The authors highlighted a way to control the surface topography by varying the electron dosage and polymerization conditions. Ballav *et al.* [130] performed EBCL not on aromatic but on aliphatic SAMs. A primary octadecanethiol layer was irradiated by an electron beam, causing structural defects. In that study, the patterning was achieved via an irradiation-promoted exchange reaction (IPER), in which irradiated molecules reacted with 11-aminoundecanethiol hydrochloride. Subsequently, the esterification of the amine end-group by bromoisobutyryl bromide was carried out, creating the ATRP initiator extremity for the polymerization of NIPAAm.

Furthermore, Vieu *et al*. [131] showed that a careful optimization of EBL processes could push the resolution limits of the technique well below 10 nm. Development of the “resist” has become an important factor in achieving such high resolution. PMMA is one of the most popular resist systems. For instance, Ahn *et al*. [132] demonstrated the possible combination of gold patterned and surface-initiated polymerization (SIP) to form micro- and nano-structures of PNIPAAm. A first layer electron-sensitive resist film of PMMA (~30 nm thick) was spin-coated onto a clean silicon surface and annealed at 160 °C for 20 min. Nanometric spots (around 3 nm in diameter) were formed under electronic beam irradiation, followed by successive depositions in organic solution. Jonas *et al*. [133] combined electron beam lithography and gas phase silanation in order to nanopattern the silicon wafer. Arrays of circular holes of varying diameter (from 35 nm to 5 μm) were created in 100 nm thick PMMA films spin-coated on silicon wafer using an electron beam. Before fixing monochlorosilane with an α-bromoisobutyrate endgroup, the bottoms of the holes were cleaned by a short exposure to oxygen plasma. Subsequently, the PMMA resist was removed with acetone Soxhlet. After the backfilling of bare silicon, the poly(2-(2-methoxyethoxy)-ethyl methacrylate) (PMEO2MA) was grown from the pattern of the ATRP initiator.

### 3.3. Scanning Probe Writing

Patterning by direct writing of the chemical reagents on specific regions of substrate is divided into several categories [134], including dip-pen nanolithography, nanoshaving, nanografting, anodization lithography, and nano-oxidation.

**Figure 11.**
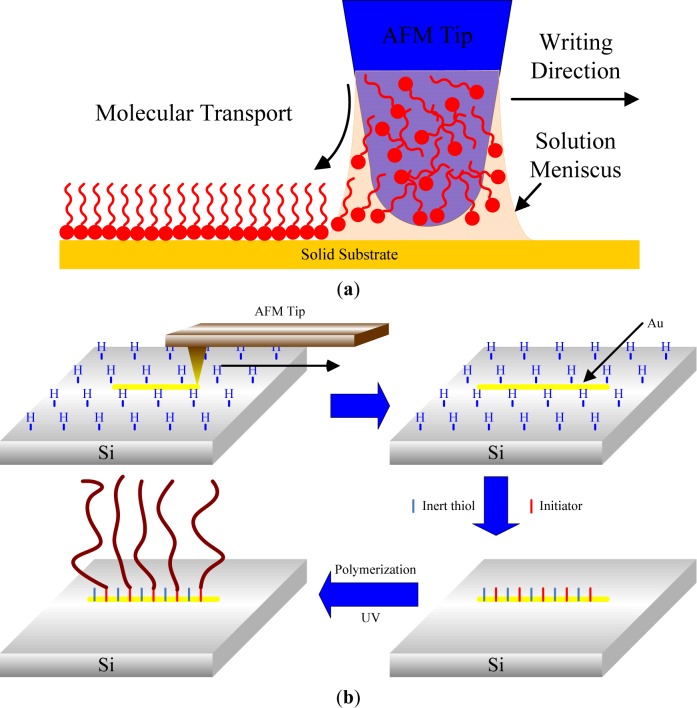
(**a**) Schematic representation of the dip-pen nanolithography (DPN) process. A water meniscus forms between the AFM tip which is coated with “ink” molecules and the solid substrate; (**b**) Preparation of polymer brushes grafted from immobilized precursors on gold nanowires. Reproduced with permission from reference [139]; Reprinted from Reference [135,139] with permission.

In 1999, Mirkin’s group introduced a new nanolithographic method called dip-pen nanolithography (DPN) [135]. DPN is a direct-write, scanning probe-based lithography process in which an AFM tip is used to deliver chemical reagents via capillary forces directly to nanoscopic regions of a targeted substrate. It was also shown that this technique offers the ability to pattern multiple chemical species (like μCP) with sub-100 nm alignment [136] (Figure 11a). The resolution of patterning depends on the volume of the meniscus, scan speed, surface chemistry, temperature, and ambient humidity [135]. Liu *et al.* [137] in 2003 reported the first combination of DPN and the surface-initiated polymerization technique in order to produce polymer brushes. 10-(exo-5-norbornen-2-oxy)decane-1-thiol molecules were deposited on a gold surface by bringing a tip coated with norbornenylthiol into contact with the substrate. Subsequently, the patterned surface was backfilled with an inactive thiol, namely 1-decanethiol. In order to amplify norbornenylfunctionalized monomers in this specific area using ring-opening metathesis polymerization, the substrate was previously activated by immersion in a Grubbs catalyst solution. Line- and dot-arrays of polymer brushes were thus built. In the same way, Ma *et al.* [138] described the first SI-ATRP from ATRP initiator deposited by DPN. This fabrication strategy is similar to the previous one. In addition to the patterning of ω-mercaptoundecyl bromoisobutyrate using AFM-tip, a gold surface was backfilled with non-functional molecules and oligo(ethylene glycol) methyl methacrylate was grown.

Zapotoczny *et al*. [139] highlighted a new nanofabrication strategy based on the tip-assisted deposition of gold nanowires on hydride-terminated silicon (Figure 11b). Disulfide initiation-transfer-termination (Iniferter) agents were selectively immobilized on the designated substrate. Linear poly(methacrylic acid) brushes, with a width from several hundred to 20–30 nm and a controllable height, were synthesized by photopolymerization of methacrylic acid from disulfide extremity. Other groups highlighted the procedure of nanoshaving SAM in order to generate polymer brushes. Kaholek *et al.* [140,141] first described the selective removal of thiol from a precoated gold surface with SAM of octadecanethiol (ODT) using an AFM tip. Large normal forces (50 nN) and high scan speeds (20 μm/s) were employed to remove the non-functional thiols and to create a pattern of straight “trenches” on the substrate. After that, the surface was exposed to ATRP initiator solution in order to selectively anchor the molecules on the bare area of the substrate. Finally, the line pattern of the thiol initiator was chemically amplified by ATRP at room temperature, creating nanopatterned PNIPAAm brushes.

Researchers have combined two patterning techniques, nanoshaving and DPN, to produce microstructures, nanodots, and nanolines of ATRP initiator. Liu *et al*. [142] focused on the patterning of ATRP initiator on a 16-mercaptohexadecanoic acid (MHA) passivated gold surface by DPN. First, the AFM tip was immersed in an ethanol solution of ω-mercaptoundecyl bromoisobutyrate. Subsequently, the initiator inked-tip was in contact with the MHA monolayer. Under high load (>10 nN), the molecules of the SAM were mechanically cleaved away by the AFM tip, and the initiator molecules were transferred onto the uncovered area of the surface. Then the SIP of NIPAAm, 2-(methacryloyloxy)ethyl trimethylammonium chloride and N,N,N,N,N-pentamethyldiethylenetriamine were successfully produced from these patterns.

Recently, Morsch *et al.* [143] performed ATRP grafting of PMMA brushes onto a pulse plasma-deposited poly(vinylbenzyl chloride)/poly(N-acryloylacrosine methyl ester) bilayer. Scanning probe lithography was employed to selectively remove the upper layer and then the underlying halide initiator of poly(vinylbenzyl chloride) initiator sites, which readily undergoes localized ATRP of methacrylate. The molecular scratch-card technique has successfully demonstrated the ability to create nanoscale polymer brush structures. Lee *et al.* [144,145] elaborated another way to pattern a SAM using AFM anodization lithography, a form of field-induced scanning probe lithography. Anodic oxide patterns were generated on an octadecylmethyldiethoxysilane (ODMS) SAM-coated silicon surface. Then specific molecules were covalently linked to the patterned area, allowing the Grubbs catalyst to be attached successfully.

Finally, the SI-ROMP of either cyclooctatetraene or 5-ethylidene-2-norbornene from patterns with a line width of about 200 nm or a dot diameter of about 75–100 nm was achieved. Based on a similar method, Benetti *et al.* [146] succeeded in forming silicon oxide nanopatterns by AFM-assisted scanning probe oxidation (SPO). By applying negative bias voltages to a gold-coated AFM-tip in contact with a monolayer of octadecyltrichlorosilane, silicon oxide nanopatterns of different sizes and shapes were obtained. Selective functionalization of patterns (dots and lines) with ATRP initiator and subsequent polymerization of HEMA were carried out. The lateral resolution of patterns confirmed the isolated grafting of a few tens of macromolecules. The electrochemical-oxidation process of a silane monolayer has been previously demonstrated by Becer *et al.* [147] using a copper TEM grid.

### 3.4. Printing Technique

**Figure 12.**
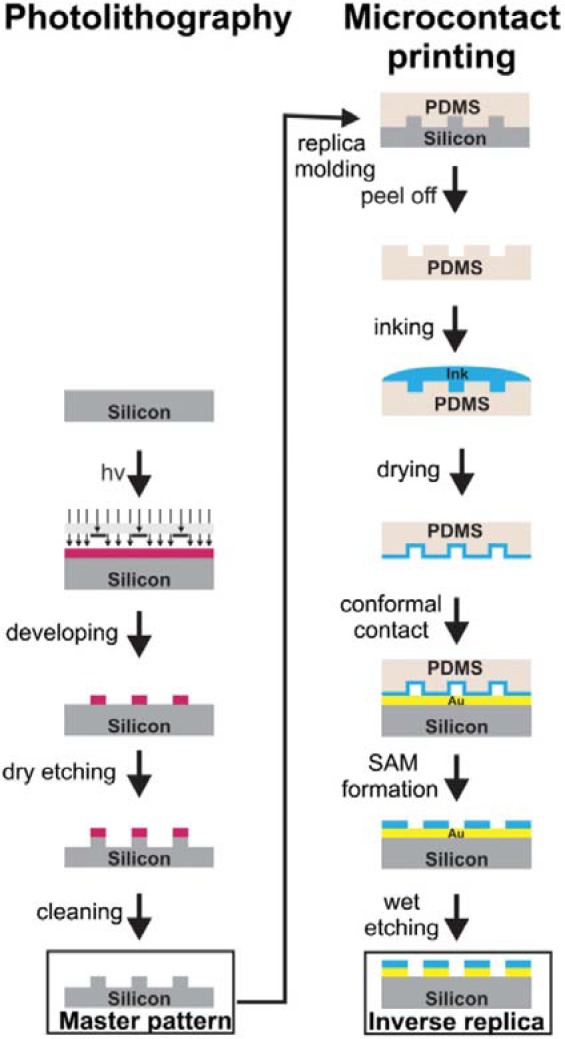
Schematic comparison of photolithography *vs*. microcontact printing (μCP). The crucial step in both techniques consists of the accurate transfer of the patterned etch-resist layer. Reprinted from Reference [150] with permission.

Among the printing techniques, we can distinguish different approaches such as micromolding in capillaries (MIMIC) [148], microtransfer molding (mTM) [149], and microcontact printing (μCP) (Figure 12) [150]. These stamping methods involve the direct patterning or the deposition of the ink molecule on the substrate using elastomeric material. Microcontact printing is probably the most versatile and cost-effective method for the generation of patterned SAMs with a lateral dimension of ≥100 nm [151].

#### 3.4.1. Microcontact Printing

Microcontact printing is an efficient technique for the patterning of large-area surfaces (planar and curved). The principle of the technique is simple and comparable to printing ink on paper with a rubber stamp. PDMS, which is considered to be the conventional stamp material in soft lithography, has shown great performance in micrometer-scale processes. In some particular studies, rigid silicon is used as the stamp support after treatment of low surface energy components to moderate the deformation and distortion of the material during the printing [152]. First, a poly(dimethylsiloxane) (PDMS) stamp is impregnated with a solution of “ink molecules”, after which it is placed in contact with the substrate. The μCP approach requires a stamp that can make direct molecular contact with the metal and an ink that can bind sufficiently strongly to the metal [153]. One of the main advantages of this technique is that a large variety of “ink molecules” can be deposited by the μCP method.

To produce topographically-patterned surfaces, μCP can be used to directly print the initiator or print a non-initiating derivative and then backfill with initiator precursors. These strategies are mainly performed in order to produce patterned polymer brushes. Kelby and Huck [152] recently elaborated a way to produce free-standing Au-polyelectrolyte brush bilayer objects. Poly(methacryloxyethyltrimethylammonium chloride) (PMETAC) brushes were grown from thiol end-groups previously deposited on a gold surface by μCP. After SI-ATRP, the metals (Au and Cr) around the brushes were removed by chemical etching. Authors created free-standing Au- PMETAC brush bilayer objects in order to quantify the mechanical stresses present in stimulus-responsive polyelectrolyte brushes. Olivier *et al.* [154] focused on the μCP of thiol initiator on a gold surface, followed by the polymerization by ATRP of N,N′-dimethyl aminoethyl methacrylate, yielding PDMAEMA brushes. The selective adsorption of carbon nanotubes (CNTs) on a pH-reversible PDMAEMA patterned gold surface was also investigated. In acidic conditions, CNTs were selectively adsorbed onto the polymer brushes due to ammonium-π interactions. The reversibility of the process was demonstrated by successive treatments in both alkaline and acidic solutions. In contrast to that on a thiol-gold surface, the patterning strategy of trichlorosilane molecules involved an additional step. A silicon surface requires activation by plasma oxidation in order to increase the proportion of hydroxyl groups and immobilize the initiator molecules through microcontact printing. Chen *et al*. [155] demonstrated another way to deposit initiator molecules. They demonstrated the successful attachment of ω-bromoundecyltrichlorosilane initiator to the carboxylic acid extremity of a molecule previously attached to a gold surface by μCP. The chemical activation of the surface was mediated by hydrogen bond attachment of the initiator. Subsequently, the amplification of NIPAAm into patterned polymer brushes was effectively achieved.

**Figure 13.**
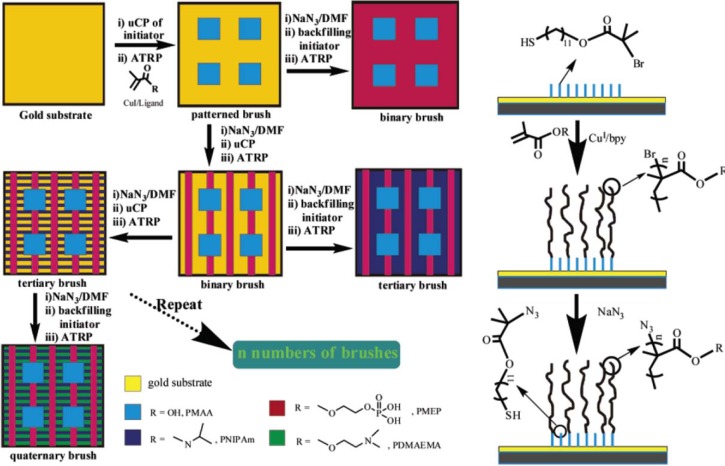
Outline procedure for grafting multiple patterned polymer brushes and ATRP passivation. Reprinted from Reference [156] with permission.

Concerning the formation of hierarchically-structured polymer brushes, Zhou *et al.* [156] presented a new strategy of multi-step microcontact printing (Figure 13). An initial concentration of thiols was used to print the first pattern, followed by a second deposition using another concentration and stamp. The concentration was adapted by dilution using nonfunctional thiol. Subsequently, poly(glycidyl methacrylate) brushes were grown. The authors demonstrated the importance of the printing order and the concentration of initiator solution. The procedure elaborated by Zhou *et al.* [156] consisted of the patterning of a gold surface with a thiol bearing a bromide end-group as initiator for SI-ATRP. Then the bromine group was deactivated by reaction with NaN_3_. The next initiator SAM was self-assembled onto the surface, and the second brush was selectively grown in this area. Tertiary and quaternary brushes were generated according to the same experimental protocol, varying the polymer thickness and the pattern design. Zhou *et al.* [156] synthesized different types of polymer brushes and generated binary, tertiary, and quaternary brushes.

#### 3.4.2. Ink-Jet Printing

Ink-jet printing, an attractive deposition and patterning technology for polymers and inorganic particles, has become an increasingly accepted tool for a lot of industrial and scientific applications including protein microarrays, DNA microarrays, color filters, gas sensors, hierarchical photocatalyst, and 3D hydrogel scaffolds for guided cell growth [157–163]. Ink-jet printing relies on the creation and release of droplets of fluids on solid surfaces on demand. Because 3000 to 12,000 ink droplets can be expelled from the nozzle of an ink-jet printer within 1 s, the jetting behavior is related to the rheology variation of a dilute solution under high shear. An *in situ* drop formation system has been used to study the high-shear-rate rheology (dynamic surface tension and dynamic viscosity) of a solution with a viscosity lower than 3 cps, which no commercial rheometer can measure. The effects of the surfactants and firing conditions on the jetting behavior of the ink-jet ink have been examined [164]. The rheology of jet inks can be adjusted by changing the surfactants and monomers to achieve good jetting directionality, uniform droplet size, and excellent wetting on the substrate [165]. UV-curable [166,167] and dual curable monomers and oligomers [168,169] can be used in the compositions of ink-jet fluids. Wang *et al.* [170] reported the ink-jet printing of colloidal photonic crystal (PC) microdots that demonstrate the fastest response (about 1.2 s) to water vapor. Such an improvement of the response rate can be attributed not only to the small size of the inkjet microdots but also to the reversible phase transition of PNIPAAm, which leads to the modulation of wetting/adhesion properties of adsorbed water on the polymer segments.

The final pattern is formed when the solvent evaporates. This technology can be adapted to deposit solutions of alkanethiols on metal surfaces to generate SAM patterns with features of around 100 μm in size [171]. Sankhe *et al.* [172] reported the use of ink-jet printing for precise placement of thiol-terminated ATRP initiator molecules on gold substrate for developing patterned and graduated soft surfaces. Subsequently, the SI-ATRP of methyl methacrylate and the characterization of resulted polymer brushes were carried out by FTIR and AFM. Recently, Emerling *et al.* [173] elaborated a new method to pattern polymer brushes on the micrometer scale. An inkjet printer was used to deposit droplets of acid onto a monolayer of ATRP initiator. Consequently, the ester bond of the initiator was cleaved by the acid, giving rise to an inactive molecule. Afterwards, the SI-ATRP of MMA was carried out and used as a good way to check the effectiveness of hydrolysis. Many parameters such as the nature of the acid, the concentration, and the reaction time were controlled in order to obtain the best pattern.

## 4. Application of Nanostructured Stimuli-Responsive Hydrogels (NSRH)

An emerging application for stimuli-responsive hydrogel that has, to date, received little attention but where stimulus-responsive polymer brushes promise to have a significant impact, is their use as tools in micro/nanomaterial fabrication. NSRHs offer unique properties, for they often undergo large changes in surface energy when switching their conformation upon application of an external stimulus. NSRHs, in combination with micro- or nanostructured surfaces, even allow property switching. To achieve a macroscopically observable change in surface properties, two fundamental design challenges need to be addressed simultaneously: (1) molecular switching entities have to be designed and synthesized; and (2) the ordered organization of molecular switching units is essential to ensure co-operative behavior of the switching units. If small molecules are employed as switching units, careful design must not only address the ability to undergo configurational or conformational transitions between different molecular states in response to an external stimulus but also ensure high degrees of alignment among molecular switching units. NSRHs have been utilized in various forms, including cross-linked (permanently) hydrogels, reversible hydrogels, micelles, modified interfaces, and conjugated solutions.

Hydrogels are formed with a three-dimensional (3D) network of polymer chains, where some parts are solvated by water molecules but the other parts are chemically or physically linked with each other. This structure has the interesting property that the chains swell but do not dissolve in an aqueous environment. Therefore, hydrogels can come from a cross-linked network of hydrophilic polymers in water, as the meaning of the prefix “hydro” is “aqueous”, and they maintain their 3D structure after absorbing large amounts water and swelling. Based on these cross-linked networks of hydrogels, the dimensions of stimuli-responsive hydrogels could be dramatically changed by an alternative change of hydrophobicity and hydrophilicity in the molecular structure of the swollen polymer chains [174]. This type of hydrogel has a crosslinked network structure in which the stimuli-responsive component is in the polymer chains, which causes dramatic swelling/deswelling according to the change in stimuli. Other forms of stimuli-responsive hydrogels could be reversibly transformed to solutions due to changes in environmental stimuli, showing solution-gelation (sol-gel) transition by altering the hydrophobic interactions of cross-linked areas in an aqueous system [175]. Therefore, this type of stimulus-responsive polymer has been developed for a phase change rather than a dimension change, to be used, for example, as injectable hydrogels [176]. Polymeric micelles could be another form of stimuli-responsive polymer system. Micelles form by aggregation of amphiphilically-combined block or terminally-modified polymers in aqueous medium, and originated from a hydrophobic effect. The modified interface can provide a dynamic on-off system by changing the hydrophobic/hydrophilic surface function and the pore size of porous membranes [15,177]. The solubility of stimulus-responsive polymers can be controlled by changing the stimuli.

### 4.1. Biotechnology

#### 4.1.1. Purification of Biomacromolecules

**Figure 14.**
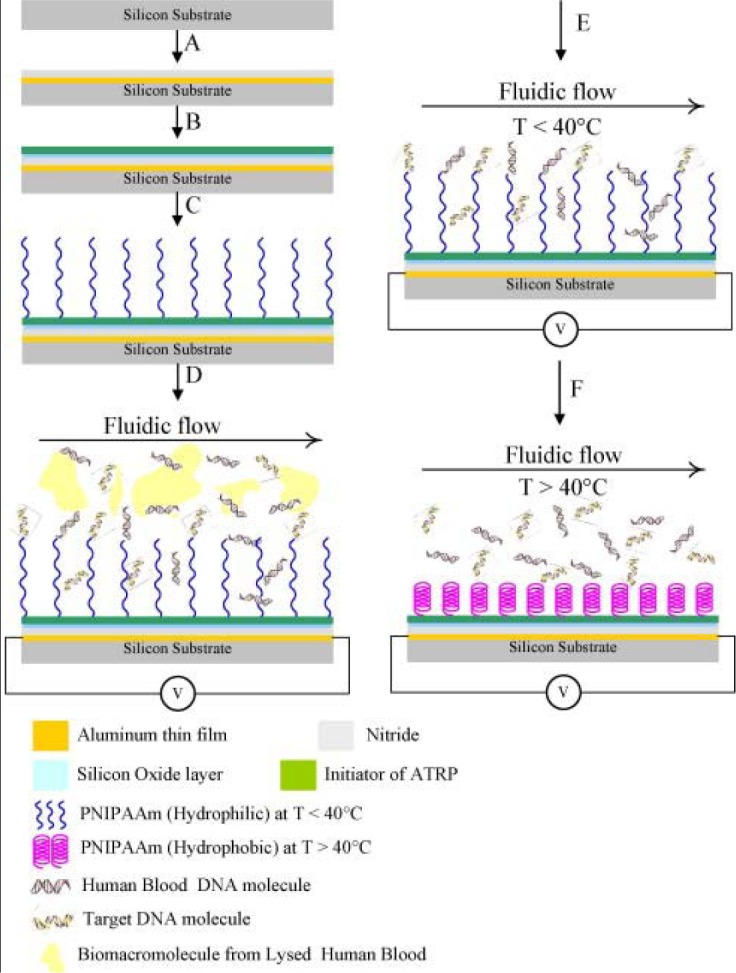
Schematic of the process for capture and release specific DNA by a tethered poly(N-isopropylacrylamide) (PNIPAAm) in the channel surface of a fluid device from a specimen with lysed human blood cells. Reprinted from Reference [181] with permission.

Targeted biomacromolecules, such as DNA, protein and antibody, can be separated from aqueous solution containing undesirable impurities by affinity binding of the targeted proteins onto a temperature responsive polymer with covalently coupled ligands specific to the target protein. Once the targeted proteins are bound onto the ligands, the proteins can be recovered from the polymer system by reversible hydrophobic/hydrophilic changes of this polymer system. One strategy of DNA purification is to immobilize the ion exchange of layered double hydroxides (LDHs) by PMMA brush on the surface [178,179]. In addition, DNAs could be captured from specimens by tethered PNIPAAm and then released above the LCST [180]. By incorporating PNIPAAm moiety on a silicon surface, the temperature control of this surface provides effective purification of specific DNA [181] (Figure 14).

The monolayer temperature can be controlled by a micro-hot plate device containing gold or platinum heater lines deposited on the thin layer. Another approach to separate biomacromolecules is to graft or modify ligand-conjugated polymer chains on column beads and separate specific proteins from flowing solutions. The separated proteins, which are bound by the immobilized ligands, can be reversibly recovered from the surfaces of columns by adjusting the temperature to under the LCST [182]. Purification of antibodies has been achieved by using temperature responsive PNIPAAm and dextran derivative conjugate as a model [183]. The main idea is the combination of the temperature sensitivity of PNIPAAm and the affinity of antibodies recognizing the polysaccharide antigen, carboxymethyl dextran benzylamide sulfonate/sulfate. This conjugate was obtained by grafting amino-terminated PNIPAAm onto this dextran derivative. It was confirmed that the purified antibodies and the polymer conjugates could be readily separated and recycled by a thermally-dependent recovery process, which gave a rapid and sensitive procedure to separate antibodies. Temperature responsive polymers were also reported to separate proteins by inducing the refolding process of proteins [184].

Another possibility for the design of switchable surfaces based on reversible motion of surface-grafted polymer chains is to incorporate active elements (nanoparticles or proteins) in the polymer layer. Switching the conformations of polymer chains results in switching of the accessibility of these active elements. Thereby, active elements are accessible or inaccessible when the polymer chains are collapsed or swollen, respectively. Minko *et al.* [116] have used this principle to design composite surfaces with adaptive adhesion prepared by grafting poly-(ethylene glycol) (PEG) chains between fluorinated particles. PEG and fluorinated particles are non-sticky in aqueous and dry environments, respectively. It was shown experimentally that PEG chains are collapsed in air and are hidden under fluorinated particles, which makes the composite surface non-adhesive in the dry state. On the other hand, in an aqueous environment, non-sticky PEG chains swell and screen sticky particles, which also makes them non-adhesive in an aqueous environment. Another possibility for using the conformational changes of polymer chains is to design systems with switchable catalytic and enzymatic activity. Typically, active species (enzymes or catalysts) are incorporated in the polymer layer. Switching of the conformation of polymer chains switches the accessibility of these active species and, as a result, leads to a change of their apparent activity. Following this concept, Ballauff *et al.* [185] incorporated silver nanoparticles in a thermoresponsive polymer brush grafted onto spherical microparticles. By changing the conformation of the polymer chains, they controlled the accessibility of the nanoparticles and in this way were able to reversibly switch their catalytic activity. Ionov *et al.* [186] used a similar approach to control biomolecular transport by temperature. The approach is based on the fabrication of a composite surface, where functional kinesin motor-molecules are adsorbed onto a substrate between surface-grafted polymer chains of thermoresponsive poly(N-isopropylacrylamide). It was demonstrated that motor-driven microtubules undergo reversible landing, gliding, and release in response to conformational changes of the polymer chains. Moreover, it has been demonstrated that such systems can be used for dynamic sorting of protein assemblies *in vitro* [187].

Grafting two or more types of polymer chains allows the design of an interesting class of responsive materials—mixed homopolymer or block-copolymer brushes [188]. Mixed polymer brushes consist of two or more sorts of polymers randomly grafted to a substrate. The mechanism of responsiveness of the mixed brushes is different from that of homopolymer brushes. Polymer chains of different sorts try to avoid unfavorable contacts and undergo lateral *versus* vertical nanoscale separation, the character of which depends on the incompatibility of the polymers and the interaction with surrounding media (solvent). Depending on the interaction of the polymer chains with the surrounding solvent, these brushes can be switched between states when polymer chains of one or the other kind are swollen and dominate at the topmost polymer layer [189]. By selecting an appropriate solvent, one can achieve a whole spectrum of intermediate states. Mixed and block copolymer brushes have been recently used in designing surfaces with switchable wettability [190], adhesion [191], and controlling protein adsorption [192], and were applied in the design of active elements in microfluidic devices [193]. Mixed polyelectrolyte (PEL) brushes represent a particularly interesting case of mixed polymer brushes. Mixed PEL brushes consist of two oppositely-charged polyelectrolytes [194]. Being responsive to pH and salt concentration, thin polymer films from mixed PEL brushes are of high interest for the regulation of protein adsorption [192], surface wetting [195], and the stability of pH-responsive colloids, [196] and for the development of smart coatings and microfluidic devices [193].

**Figure 15.**
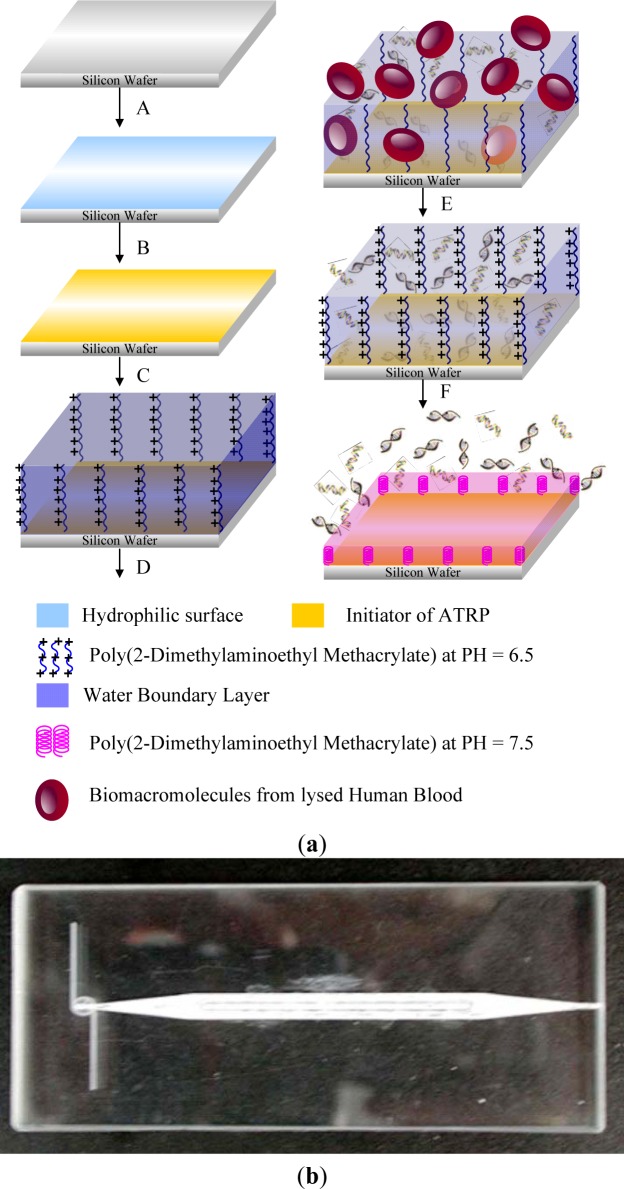
(**a**) Schematic representation of the capture and release of specific gDNA from a specimen of lysed human blood cells by tethered Poly[2-(dimethylamino)-ethyl methacrylate] (PDMAEMA) on an Si surface [200]; (**b**) Optical microscope image of the fluidic system with the straight channels. Reprinted from Reference [178] with permission.

**Figure 16.**
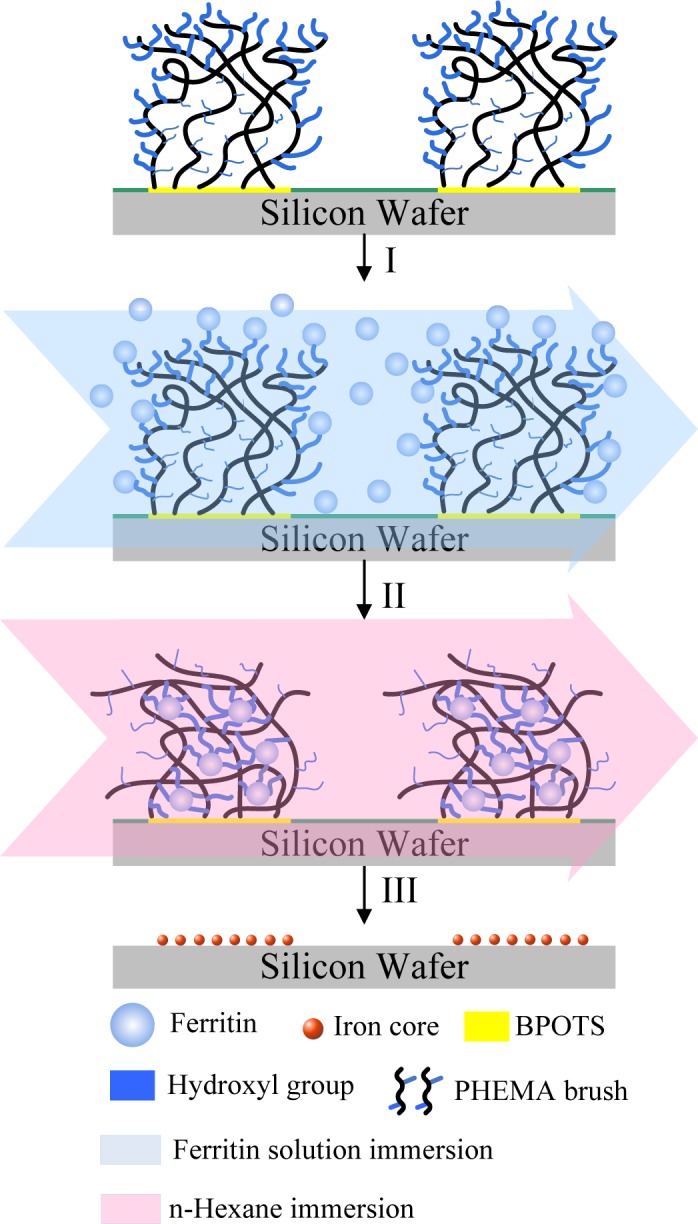
Schematic representation of the strategy for ferritin capture. (**I**) The sample surface presenting the patterned PHEMA brushes is immersed for 1 h into a mixture of water and MeOH containing dispersed ferritin; (**II**) The sample surface is immersed into n-hexane to transform it from a brush-like to a mushroom-like structure, with the OH groups of the PHEMA brushes becoming buried within the PHEMA thin film to form hydrophilic domains; (**III**) The ferritin species on the PHEMA thin film surface are removed through degradation of the protein sheath under O_2_ in an oven at *ca.* 500 °C to observe the ferrihydrite cores. Reprinted from Reference [204] with permission.

Stimuli responsive polymers could be also utilized to manufacture microfluidic systems that can self-control the microscale flow as well as separate, purify, analyze, and deliver biomolecules. A switchable DNA trap was obtained with a surface coated with an end-tethered monolayer of PDMAEMA (Figure 15) [197]. This device could adsorb and desorb the bound DNA due to the switchable hydrophilic/hydrophobic property of the thin layer by changing the pH. This surprisingly rapid response time was achieved by the tenuous scale of the polymer layer, which was *ca.* 300-nm thick. An array of these devices could be utilized to purify the targeted DNA on a large scale while preserving the rapid response time [198]. Also, this device could be applied for artificial organs, such as an artificial pancreas for controlling the release of insulin. However, such devices require an external power source, which can restrict their use in practical systems *in vivo*. Another report describing the use of stimuli-responsive hydrogels as active elements in microfluidic devices was reported by Beebe *et al.* [199]. They used pH sensitive hydrogels. Later on, the approach was extended to temperature-, enzyme-, light- and electric field-responsive polymers [200]. For lab-on-a-chip applications, the use of light and electric fields as stimuli hold promise, since they allow switching with both high spatial and high temporal resolution. For example, Richter and co-workers [201] explored possibilities to control liquid flow with stimuli-responsive hydrogels. In particular, they used hydrogels as pumps and gates which control the liquid flow in microfluidic devices. They fabricated electrically controlled arrays of heating elements to locally heat thermoresponsive hydrogels and in this way designed a display for the Blind. Recently, Minko *et al.* [202] have developed an approach for fabricating porous hydrogel membranes, which they used for biochemically-controlled gating of a liquid flow. Recently, Chen’s group introduced a novel approach to capture ferritin from a fluidic system [203]. Well-defined patterns of polymerized 2-hydroxyethyl methacrylate (HEMA) brushes were grafted from a silicon surface, and the switchable properties by change of environmental solvents were explored. The PHEMA brushes behaved as “tentacles” that captured ferritin complexes from aqueous solution through entanglement between the brushes and the ferritin proteins, whose ferritins were trapped due to the collapsing of the PHEMA (Figure 16) [119]. High-resolution scanning electron microscopy has been used to observe patterned ferritin iron cores on the Si surface after thermal removal of the patterned PHEMA brushes and ferritin protein sheaths.

#### 4.1.2. Biological Interfaces

A temperature responsive surface or interface is another route to biomedical applications such as temperature-modulated membranes [204,205], chromatography [206], and cell sheets [207]. PNIPAAm has been extensively investigated to develop temperature responsive intelligent surfaces and interfaces because of its specific advantages. Two categories can be considered for interfaces designed with immobilized temperature responsive polymers (broadly all stimuli responsive polymer); to modulate pores of a porous matrix as temperature responsive gates, and to control the wettability of nonporous matrix surfaces. For example, the modification of a membrane surface with temperature responsive polymer chains can modulate the diffusion profiles. It is well known that a PNIPAAm-grafted porous membrane shows positive control of solute diffusion, which means that the fast diffusion occurs through the opened gates at higher temperature [208], while a PNIPAAm-grafted nylon capsule exhibits negative control of solute diffusion by blocking the solutes from passing through the surface of the membrane above the LCST of the temperature responsive polymer [209]. Recently, it has been reported that the surface of microporous polypropylene membrane with PNIPAAm was modified by a plasma-graft-filling polymerization technique [210]. A novel mechanism for switching the permeation of hydrophobic and hydrophilic solutes by changing temperature, which could give the stepwise separation of solutes from the mixed solution, was demonstrated. Above the LCST, only hydrophilic solutes permeated the hydrophobic pore because the hydrophobic solutes adsorbed onto the hydrophobic pore surfaces. In contrast, below the LCST, the pore surface became hydrophilic, and hydrophobic solutes desorbed from the pore surface and condensed in the permeate side. Using a similar mechanism, a switchable molecular filter was fabricated by encapsulating PNIPAAm into a porous silica membrane by a sol-gel process [211]. Under the LCST, the pores became hydrophilic to prevent water and PEG permeation, and above the LCST, they became hydrophobic to permit the flow of aqueous solutions of PEG of low molecular weight (%5000 Da) while blocking PEGs of higher molecular weight. The reversible on/off permeation and molecular cut-off filtration behavior of these membranes has also been described above and below the transition temperature of PNIPAAm [212]. Elastin like polypeptide (ELP) was reported to replace PNIPAAm as a protein-based molecular switch [213]. Demonstrating the same mechanism as the PNIPAAm-silica hybrid membrane, the ELP-silica hybrid membranes also were impermeable to all of the PEG solutions below the LCST of ELP, while above that LCST, they were permeable only to PEG with molecular weights of less than 5000 Da. In the case of surface modification by grafting polymer chains onto a membrane, some drawbacks have been discussed. The application of smart coatings to filtration systems can possibly improve filter life, streamline cleaning cycles, or confer antimicrobial properties to the surface. This process changes the pore size and pore size distributions, decreasing permeability and also causing different graft densities between the external surfaces and internal pores [214]. Recently, a temperature-responsive microfiltration membrane was fabricated as an alternative to modifying a porous membrane surface with a temperature responsive polymer [215]. These membranes were proposed to overcome the disadvantages described above. In that study, the membranes were prepared from PNIPAAm-g-poly(vinylidene fluoride) (PVDF) copolymers by the phase inversion method, wherein the copolymer solution is cast onto a glass plate and immersed in a nonsolvent, such as water, after a brief evaporation of solvent in air. The PNIPAAm-g-PVDF was synthesized from ozone-preactivated PVDF. The casting temperature was an important factor for these membranes. The membrane cast under the LCST of NIPAAm showed extensive aggregation of the NIPAAm component onto the surface due to the hydrophilic interaction between NIPAAm chains and water. Therefore, the flux of water exhibited a strong and reversible dependence on the permeation temperature due to the surface property of PNIPAAm. Among others, Lokuge *et al.* [138] were able to show that grafting stimulus-responsive polymer brushes to membrane surfaces is an effective route to obtaining controllable, active filtration capabilities. Their nanocapillary arrays, made of Au-coated track-etched polycarbonate, consisted of 80–200 nm regularly-arranged pores. In their approach, physical changes in a surface-grafted PNIPAAM film can be triggered by heating the polymer above its LCST, which switches on the membrane permeability for dextran molecules because the effective membrane pore diameter increases with decreasing film swelling above the LCST. If the molecular weight and graft density is tuned so that brush height is on the order of the pore diameter, the hydraulic permeability can thus be controlled. Cultivated mammalian cells can be easily recovered from substrate surfaces by introducing temperature responsive polymers into the cell culture dish. In general, mammalian cells are cultured on a hydrophobic substrate and recovered by using protease such as trypsin, which might damage the cultured cells. Without any use of such a harmful enzyme, PNIPAAm-grafted substrate surfaces have been proposed for convenient and safe culture dishes; their hydrophobic surfaces above the LCST can become hydrophilic below the LCST [216]. At 37 °C, cells such as lung cells [217] showed good adhesion and proliferation on this culture dish, similar to those in commercial polystyrene dishes. However, below the LCST, when the surface was hydrophilic, cells detached from the surface. This switchable property of a cell culture surface was systematically studied by monitoring blood platelet movement depending on the temperature change from 37 to 20 °C [218]. In a more advanced study, temperature-regulated cell detachment was reported to require cell metabolic activity, requiring ATP consumption, signal transduction, and cytoskeleton reorganization [219]. The intact microglia cells harvested from this culture dish were used for a novel cell transplantation therapy for damaged central nervous system tissue [220].

Recent progress in cell cultures is based on more effective cell culture and detachment. One approach is to introduce bioconjugates of temperature-responsive polymers with cell adhesive motifs [221]. The temperature responsive hydrogel beads were conjugated with a cell adhesive motif only on their outer surface. These beads could more efficiently cultivate phenotypes expressing chondrocytes due to a large surface/volume ratio. The well-attached and proliferated chondrocytes were successfully detached from the culture carrier below the LCST. Carboxylic acid groups were also incorporated with a PNIPAAmbased culture dish to accelerate cell detachment below the LCST of the polymer surface [0]. However, acrylic acid introduced by copolymerization [poly (NIPAAm-AAc)] resulted in excessive surface hydration and hindered the spread of cells in culture at 37 °C. To obtain two benefits (e.g., good cell adhesion and accelerated cell detachment), the authors introduced 2-carboxyisopropylacrylamide (CIPAAm), having both a similar side chain structure to NIPAAm and a functional carboxylate group. The P(NIPAAmco-CIPAAm) showed nearly the same LCSTs and temperature sensitivity as the NIPAAm homopolymer. It also exhibited hydrophobicity similar to homopolymer PNIPAAm-grafted surfaces at 37 °C, as well as an accelerated cell detachment time, which might have come from its more rapid deswelling than that of PNIPAAm homopolymer due to the introduced carboxylic acid moiety. For 90% cell detachment, they observed that 120 and 60 min were required for PNIPAAm homopolymer and P(NIPAAm-co-CIPAAm) culture dishes, respectively.

### 4.2. Switchable Wettability

Switchable wettability of a surface is another strategy for manufacturing intelligent surfaces. Four models of graft chain conformations on the surface were suggested, and their temperature responsive wettability changes were studied on the PNIPAAm grafted surface [222]. The reported four models were: (1) free end and linear grafts onto the surface; (2) muli-point looped grafts onto the surface; (3) free end grafts onto a looped chain modified surface; and (4) a thin hydrogel layer modified surface. In the case of (2), the transition temperature of surface wettability fell below the LCST of PNIPAAm (32 °C). When the density of PNIPAAm chains increased, as in case (3), a large change of wettability was observed at the transition temperature. However, the thin hydrogel layer modified surface case (4) was reported to undergo less wettability change, which was explained by the restricted chain mobility caused by the cross-links.

**Figure 17.**
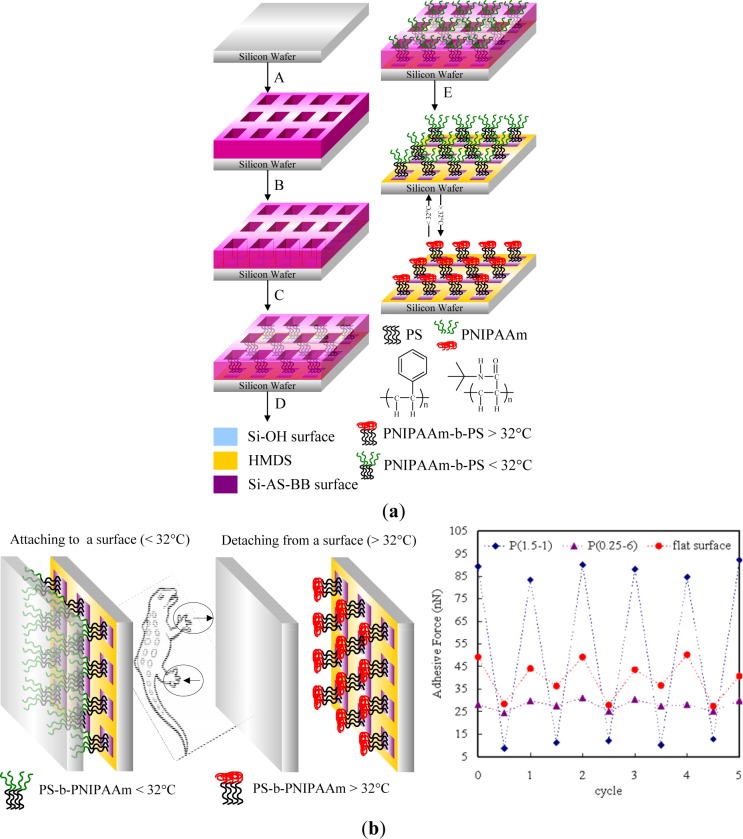
(**a**) Schematic representation of the process used to fabricate chemically nanopatterned surfaces of PS-b-PNIPAAm brushes; (**b**) Reversible Hydrophobic/Hydrophilic adhesive of PS-b-PNIPAAm copolymer brush nanopillar arrays for mimicking the climbing aptitude of geckos. Reprinted from Reference [223] with permission.

Since some patterns generated by dewetting can hardly be achieved by other techniques, they can be used as original molds to reproduce the pattern or to produce even more complex patterns. In combination with some functional materials, functional structures and even some devices can be conveniently generated. Switching of macroscopic geometrical parameters of hydrogels was recently explored for designing materials with switchable optical properties. Inspired by the adjustable adhesive ability of the gecko foot pad, which alternately attaches to and detaches from a climbing surface, Chen *et al.* [15] used a fabrication process to generate well-defined pillar patterns of polymerized styrene and successively grafted n-isopropylacrylamide (NIPAAm) as thermally responsive terminations of the pillars (Figure 17). By varying the geometry of the patterns, including the aspect ratio and duty ratio (solid fraction), the respective roles of the geometry the pattern features on the static water contact angle (WCA), researchers have systematically investigated the hysteresis, adhesive, and friction forces at 25 and 50 °C. The fabrication strategy exploits surface textures of polystyrene (PS) and thermally responsive termination of PNIPAAm as the artificial foot pad surface, which could generate alternately *ca.* 93.9 and 8.7 nN of adhesive force at 25 and 50 °C, respectively. The results indicate that the adjustable adhesive ability of the copolymer brushes could approach the climbing aptitude of a gecko much more closely. The advantage of the processing strategy described here is the potential to fabricate an artificial foot pad that mimics the climbing aptitude of geckos [223]. In a similar approach, gold nanowires composed of gold nanoparticles can be fabricated [224]. Furthermore, the dewetted pattern can be used as a powerful mask to transfer a pattern onto another material. Both positive and negative replicas of the pattern can be made from the pattern. When the pattern works as a resist for chemical etching, the underlying layer under the pattern preserves the formation of the positive replica of dewetting [225,226]. There are usually two kinds of negative replicas of patterns that can be transferred from a pre-existing pattern. First, the pre-existing pattern works as a shadow mask that some other materials deposit on the blank areas by some other technique, such as vapor deposition [227]. The dewetting pattern works as a hard mold, transferring the pattern onto the PDMS elastomer when the PDMS prepolymer is cast onto the patterned surface and cured. Peeled from the hard surface, the PDMS elastomer with relief structures as negative replicas of the dewetting structures is then achieved [225,228]. These PDMS elastomers obtained from the dewetting and pattern transfer can be used as the template in a subsequent soft lithography process. More dewetting properties may be incorporated onto the PNIPAAm-grafted surface. For example, hydrophobic and ionizable hydrophilic groups were introduced in PNIPAAm chains as a random copolymer form, and this copolymer was grafted onto silica beads [229]. In this study, poly(N-isopropylacrylamide-co-acrylic acid-co-N-tert-butylacrylamide) [poly(NIPAAm-co-AAc-co-tBAAm)] hydrogel was grafted onto silica beads and evaluated as a column matrix for cation-exchange thermoresponsive chromatography. In the other study, 2-carboxyisopropylacrylamide was copolymerized with PNIPAAm, and this copolymer was grafted onto a polystyrene petri dish. Cell detachment was intensified when only PNIPAAm chains were grafted on the dish under the LCST of PNIPAAm [0].

The dimensions of surface modification have been controlled down to micro or nano-scales. Temperature responsive nanoparticle surfaces were obtained by introducing mono-dispersed polystyrene seed particles as a shape template in the two-step swelling and polymerization method. Micropatterning with a temperature responsive polymer has been investigated; PNIPAAm was pattern-immobilized by coupling reactions with surface-bound aminosilanes or by copolymerization of NIPAAm onto surface-bound methacrylsilane [230]. The method could be extended to the fabrication of undercut structures composed of other insulating polymer materials; the only requirement seems to be that the materials used must be able to dissolve in a water immiscible, volatile organic solvent.

**Figure 18.**
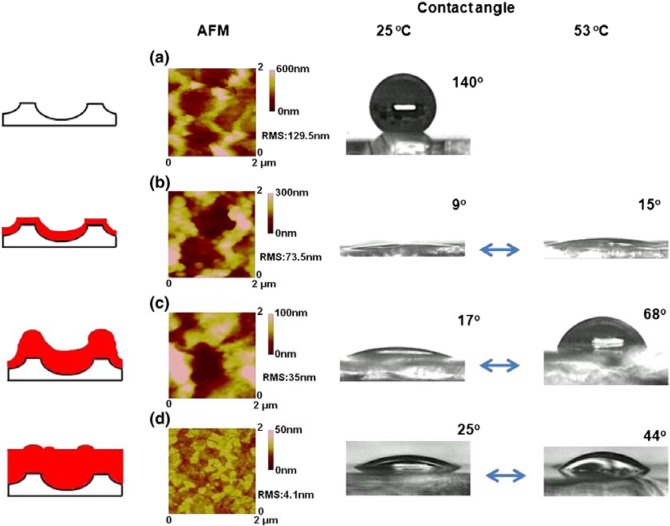
Schematic representation of the PNIPAm-grafted pore-array with different PNIPAm layer thickness, AFM images and thermally responsive wettability induced water droplet profile changes at two different temperatures of (**a**) ZnO pore-array and PNIPAm-grafted (**b**) S1; (**c**) S2; and (**d**) S3 substrates. Reprinted from Reference [231] with permission.

PNIPAAm can be grafted to an ordered surface such as a ZnO pore-array surface by surface-initiated polymerization [231]. The thermal responsive wettability switching behavior on such a surface is a compromise between the 3D capillary effect (Wenzel’s model) and the air trapping effect (Cassie’s model). The former dominates when the thin PNIPAAm layer is grafted onto the pore-array film. As the PNIPAAm layer becomes thicker, the air trapping effect dominates and the thermal responsive wettability switching is enhanced. The size of the air/water interface is controlled by the pore size, the amount of grafted PNIPAAm polymer, and the shape of the pore edge (Figure 18).

### 4.3. Sensors

It is well known that the function of a sensor is to provide information on physical, chemical, and biological environments. Legislation has fostered a huge demand for the sensors necessary in environmental monitoring, e.g., monitoring toxic gases and vapors in the workplace or contaminants in natural waters from industrial effluents and runoff from agriculture fields. Thus, a near revolution is apparent in sensor research, giving birth to a large number of sensor devices for medical and environmental technology. Since the chemical and physical properties of stimuli-responsive polymers may be tailored by the chemist to particular needs, these properties have gained importance in the construction of sensor devices.

#### 4.3.1. Optical Sensors

**Figure 19.**
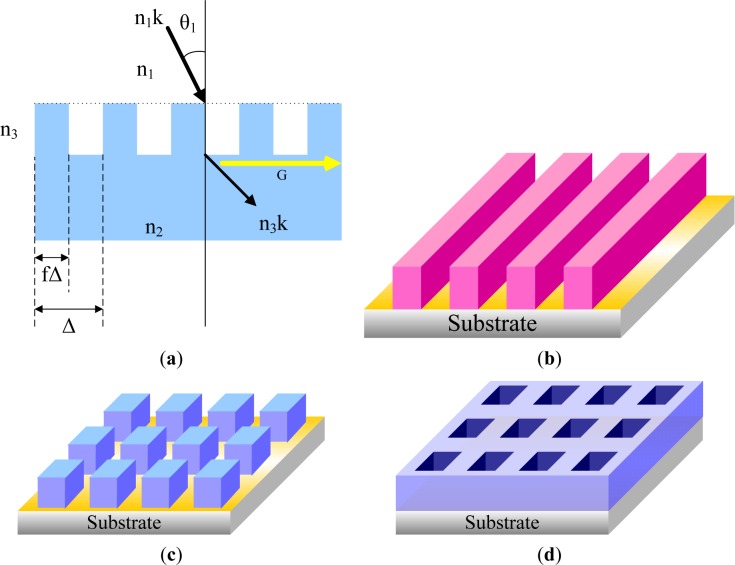
(**a**) Cross section profile of a one-dimensional periodic subwavelength grating; (**b**) One-dimensional periodic subwavelength gratings; (**c**) Symmetric two-dimensional subwavelength relief gratings; symmetric two-dimensional subwavelength concave gratings. Reprinted from Reference [232] with permission.

Subwavelength structured (SWS) surfaces are attractive for new optical elements, and many different elements with these surfaces have been developed. A mean refractive index can be controlled by the filling factors of the structure, so that a desired distribution of the refractive index can be realized. A non-symmetric structure causes optical anisotropy, called form birefringence. Moreover, when the grating period is on the order of the light wavelength, the light wave may resonate and be reflected in the structure such that resonant reflection occurs. These optical features produce new optical elements. The SWS does not generate real diffracted light waves other than the zero-order diffraction waves. Given a periodic relief grating with a period of Δ (Figure 19a), the grating vector G has a magnitude of 2π/Δ [232]. If exact values of reflectance, transmittance, and their phase retardation of SWS elements are needed, numerical calculations must be done based on electromagnetic theory. However, it is convenient that the SWS structure approximates an optically anisotropic thin film. This idea is called the effective medium theory (EMT). The effective refractive index is defined by the wave number of a propagation eigenmode in the structure. In the case of a one-dimensional binary relief grating, as shown in Figure 19b, the effective refractive index for TE polarization is *n*_TE_, and the index for TM polarization is *n*_TM_. For a two-dimensional periodic relief grating (2DPRG) and two-dimensional periodic concave grating (2DPCG), as shown in Figures 19c and 18d, the wave number of a zero order eigenmode is not given by a closed form equation. To obtain an exact value of the wave number, an eigenvalue problem must be solved for a matrix with a large size. This calculation is complex and the calculation process is exactly the same as part of the Fourier modal method of a grating analysis [233]. Moreover, even though the grating period is much shorter than the light wavelength, the wave number cannot be expressed by a closed form equation [234]. The top-down methods like lithography are mature techniques for producing micro- and even nano-patterns, which can be used to guide the grating into ordered structures. The combination of self-assembly of block copolymers and the lithograph system provides large opportunities to fabricate novel grating structures. Reducing the pattern size down to the nanometer scale is one of the most important aspects in pattern applications requiring new techniques or some new materials to utilize the grating structure. Nowadays, techniques like e-beam lithography, dip pen lithography, and so on are capable of generating nano-scaled chemical and topological patterns on silicon wafer, which can then be used to guide the grating into ordered structures.

**Figure 20.**
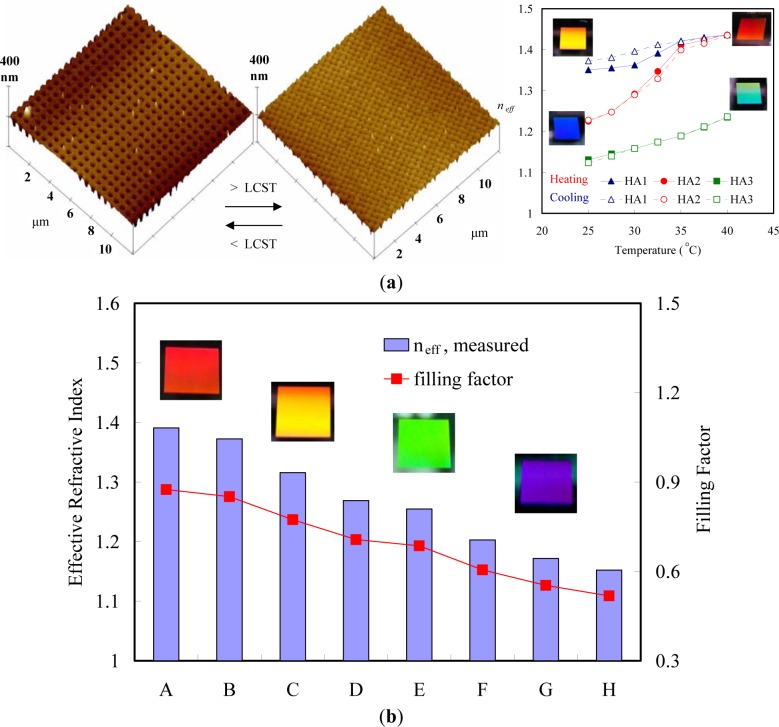
(**a**) Geometrical parameters and/or refractive index contrast of a 2DPCG typically change with the temperature resulting color change [238]; (**b**) neff response and calculated filling factor of 2DPRG of tethered PMMA layer as a function of solvent species for volatile organic compounds (VOC) exposing in the order of chloroform (A); 1,2-dichloroethane (DCE) (B); dioxane (C); toluene (D); tetrahydrofuran (THF) (E); cyclohexane (F); acetone (G); and in dry state (H). Photographic images demonstrate the grating effect of the 2DPRG under VOC exposing of chloroform, dioxane, THF, and acetone, from left to right. Reprinted from Reference [239] with permission.

Chen *et al*. [235] used a novel fabrication process involving very-large-scale integration and oxygen plasma treatment to generate well-defined patterns of polymerized (2-dimethylaminoethyl methacrylate) (DMAEMA) as a one dimensional periodic relief grating (1DPRG) on silicon surfaces. The resolutions of the line patterns of the PDMAEMA brushes approached 400 nm. The line patterns of PDMAEMA brushes could generate water contact angles (WCAs) and refractive indices for TM and TE polarization on the surface because of the one-dimensional binary periodic relief grating. The effective refractive index for TM and TE polarization could be tuned by changing the pH values. These reversibly-tunable properties are created from a reversible pH-induced swelling transition of tethered PDMAEMA. To enhance the color change of the PDMAEMA grating, gold nanoparticles were immobilized on the PDMAEMA chains or self-assembled layers [114,236]. However, a 1DPRG can display solely one pure color, visible to the naked eye, along the TM and TE directions. Nevertheless, it is very difficult for the naked eye to observe along the TM and TE directions precisely; therefore, 1DPRGs always present composed color images [236,237]. Unlike 1DPRGs, the colors of two-dimensional periodic gratings provide better stability and chromatic aberration regardless of the visual angle. Chen *et al.* [238] fabricated 2DPCG of tethered poly(N-isopropylacrylamide) (PNIPAAm) on the 200-nm-scale onto flat Si substrates. The whole array structures of tethered PNIPAAm could be created and erased reversibly at 25 and 40 °C, respectively, leading to significant changes in the effective refractive index (*n*_eff_), which in turn resulted in a color change from blue to red that could be observed by the naked eye at incident angles of 10°–20° (Figure 20a). Effective-medium theory is also exploited to calculate the filling factors of air inside the 2DPCGs to verify the structural changes of the tethered PNIPAAm. Such designed 2DPCGs of thermorespective hydrogel films have potential applications in temperature-responsive optical devices [e.g., as antireflection structured surfaces (ARSs)] that operate at both visible and near-infrared wavelengths. When a grating interacts specifically or nonspecifically with target molecules, its geometrical parameters and/or refractive index contrast will typically change. Grafted poly(methyl methacrylate) (PMMA) from a 200 nm-resolution hole array of photoresist on a silicon substrate as a pillar array of two-dimensional periodic relief grating (2DPRG) has been exploited to detect organic solvents in surrounding media, based upon the structural change of the 2DPRG as a consequence of the solvent induced reversible swelling/deswelling of PMMA chains, through an effective refractive index. Dramatic color changes, purple, green, yellow, and red, were observed with the naked eye when the surrounding media of PMMA-modified 2DPRG were acetone, tetrahydrofuran, dioxane, and chloroform, respectively (Figure 20b) [239,240].

**Figure 21.**
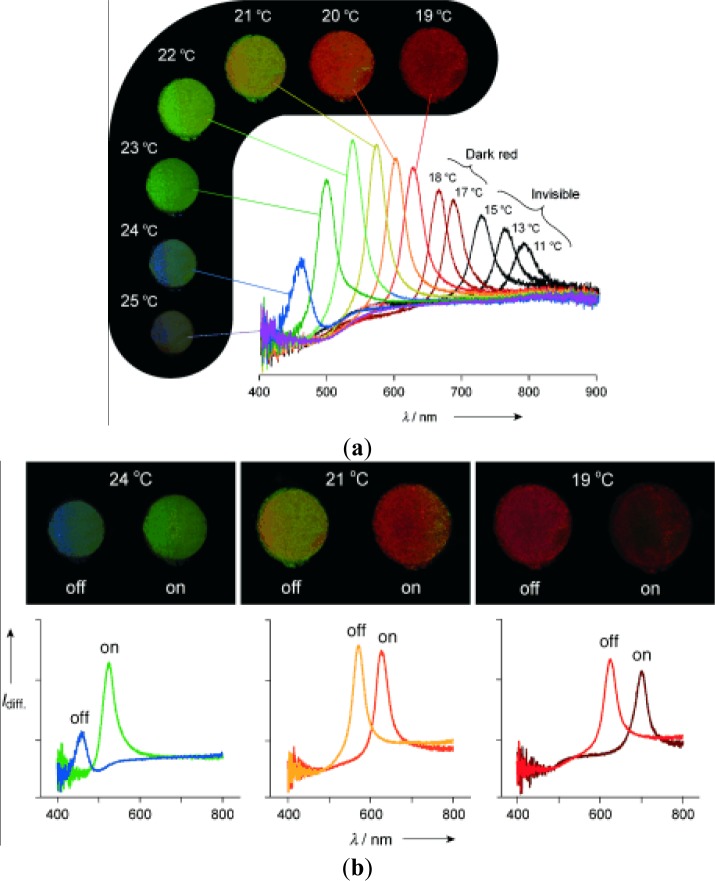
(**a**) Spectroscopic characterization of the porous gel. Photographs and reflection spectra of the porous poly(NIPA-co-AAB) gel in the dark at various temperatures; (**b**) Multicolor photochromic behavior of the porous gel. Photographs and reflection spectra of the porous poly(NIPA-co-AAB) gel in water at 19, 21, and 24 °C before UV irradiation and after the equilibrium degree of swelling had been reached in response to the UV irradiation (366 nm, 8.0 m·W·cm^−2^). Reprinted from Reference [241] with permission.

Other ordered structures combining self-assembly of block copolymers and some supermolecular systems have been exploited to fabricated visualized sensors. Photonic crystals with switchable optical properties, another type of smart optical device, can be designed using stimuli-responsive polymers. An assembly of switchable hydrogel particles with incorporated high-refractive index particles in opal-like periodic structures or a hydrogel matrix filled with closely-packed high refractive index particles are two possibilities for designing devices with switchable optical properties (Figure 21) [241,242]. In both approaches, reversible swelling/deswelling of the polymer results in changes in the periodicity of the lattice that in turn causes switching of the optical properties. Here, mechanical treatment [243], temperature [240], light, magnetic [244], and electric [245] fields are the stimuli that switch the optical properties. As in hydrogel particle composites, stimuli-induced swelling of block copolymers was used for designing opal-like photonic crystals with switchable optical properties [246]. Notably, this mechanism of switching the periodicity of high-refractive index protein assemblies is used by squids to tune their coloration [247]. Lyon *et al.* [248] suggested using switchable hydrogel particles as lenses with a tunable focal length. The hydrogel particles can be assembled in hexagonally packed arrays and are used for fabricating miniaturized elements of different size [249]. Jiang *et al.* [250] have used hydrogel actuators integrated into a microfluidic system as a container for a liquid droplet. The hydrogel reacts to stimuli by adjustment of the shape—and hence focal length—of the droplet. Another approach to designing switchable lenses was explored by Crosby *et al.* [251]. This work presented a simple, robust, biomimetic responsive surface based on an array of microlens shells that snap from one curvature (e.g., concave) to another curvature (e.g., convex) when a critical stress develops in the shell structure. Recently, Akashi *et al.* [252] have explored another bio-inspired approach to designing materials with tunable coloration. The authors designed a bio-inspired light-modulation material that imitates the behavior of the pigment cells of squids and octopuses based on hydrogels with included pigments. The mechanism of light modulation is due to the reversible volume change of dense colored gel particles; *i.e.*, light modulation is caused by a synergetic effect between the change of the area of light absorption and the absorption of colorant in gels.

#### 4.3.2. Biosensors

The term “biosensor” is used to cover sensor devices for determining the concentration of substances and other analytes of biological interest, in some cases even where a biological system is not directly utilized. The last two decades have witnessed the emergence of polymers as an intriguing class of macromolecules having electrical and optical properties that can be applied as sensors. Stimuli-responsive hydrogels are used in biosensors in three main ways: for signal detection, in transmission of a signal to a measuring electrode, or as a response element that controls the feedback response to the signal. Polymer swelling can lead to physical work, such as shutting off a valve or making contact between the sensor and a secondary component. When cross-linked hydrogel components of composite membranes are prepared with an amine containing dimethylaminoethyl methacrylate monomer, the result is a polymeric device that swells in response to pH changes (neutral to acidic medium). The enhanced biosensing capabilities of these membranes have been demonstrated in the fabrication of glucose-, cholesterol- and galactose-amperometric biosensors. The conjugation of these polymers to different recognition proteins, including antibodies, protein A, streptavidin, and enzymes, can be done in a random or site-specific manner. Different stimuli-responsive hydrogels, including temperature-, pH- and light-sensitive polymers, have been conjugated to these proteins. Once the analyte is bound to these recognition proteins, the environmental change triggers the release of these molecules. This triggered release could also be used to remove inhibitors, toxins, or fouling agents from the recognition sites of immobilized or free enzymes and affinity molecules, such as those used in biosensors [253].

There has been considerable interest in the development of DNA sensors for the analysis of unknown or mutant genes, the diagnosis of infectious agents in various environments, and the detection of substances such as drugs or pollutants that interact with the structure of the double-stranded DNA. Single strand DNA probes are immobilized by techniques such as adsorption, direct covalent binding, and entrapment in a polymer matrix. New biocomposite materials, based on the incorporation of nucleic acid dopants within an electronically conducting polypyrrole network, have been described by Wang and Jiang [254]. The growth patterns and ionexchange properties of these electropolymerized polypyrrole-oligonucleotide (PPy/ODN) films have been characterized using an *in situ* electrochemical quartz crystal microbalance (EQCM). Various parameters, such as the ODN length or concentration and the potential range, have a marked effect on the properties of the new conducting biomaterials. Very favorable growth patterns are observed for biocomposites containing 20–30-m long ODNs, while films based on shorter ODNs or chromosomal DNA display inferior properties. The composite films can be prepared using low (approximately 1 × 10^−5^ M) concentrations of the nucleic acid dopant in the absence of additional electrolyte. Such biomaterials open up new opportunities, including genoelectronic devices, composite materials, bioactive interfaces, genetic analysis, or probing of DNA charge transfer. A conjugated polymer is regarded as a three dimensional network of intrinsically conducting macromolecular wires that is able to transport electrical signals. Further functionalization of such molecules with prosthetic groups shows recognition properties, and such polymer architectures mimic the nervous system in living systems. With this idea, Garn *et al.* [255] developed new electrochemical sensors based on electroactive polypyrrole functionalized with ODN. They analyzed the experimental conditions for building such a modified electrode, showing a high electroactivity in aqueous medium. The functionalization of polypyrrole involved a precursor polypyrrole bearing an easily-leaving ester group, on which an amino-labeled ODN could be directly substituted. The electrochemical response of this polypyrrole electrode functionalized with an ODN probe was analyzed in various aqueous media containing either complementary or noncomplementary ODN targets. The results showed that the functionalized polypyrroles acted as macromolecular wires that could transduce biological information into molecular signals. Livache *et al.* [256] described an ODN array constructed on a silicon device bearing a matrix of addressable 50-μm microelectrodes. Each electrode was covered by a conducting polymer (polypyrrole) grafted with ODN. The DNA chip was prepared by successive electrochemically addressed copolymerizations of 5’ pyrrole-labeled ODN and pyrrole. This technology found successful application in the genotyping of hepatitis C virus in blood samples, with results that showed good sensitivity and a high dimensional resolution. The amphiphilic co-polymer consisting of oligodeoxyribonucleotide as the hydrophilic part and thermoresponsive PNIPAAm as the hydrophobic part has been investigated. The co-polymers formed DNA-linked colloidal nanoparticles above the phase transition temperature of PNIPAAm as the DNA was hybridized to the complimentary oligodeoxyribonucleotide. The nanoparticles aggregated rapidly when the complementary strand was added into the dispersion. In contrast, they remain dispersed in the absence of the complementary strand and in the presence of the point-mutated oligonucleotide. These distinct phenomena may be applied in an oligonucleotide analysis system in gene diagnosis [257].

**Figure 22.**
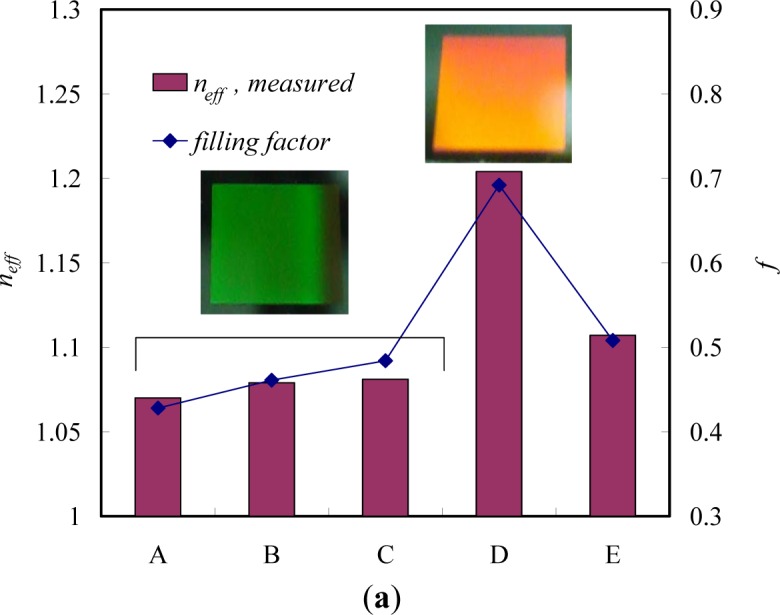

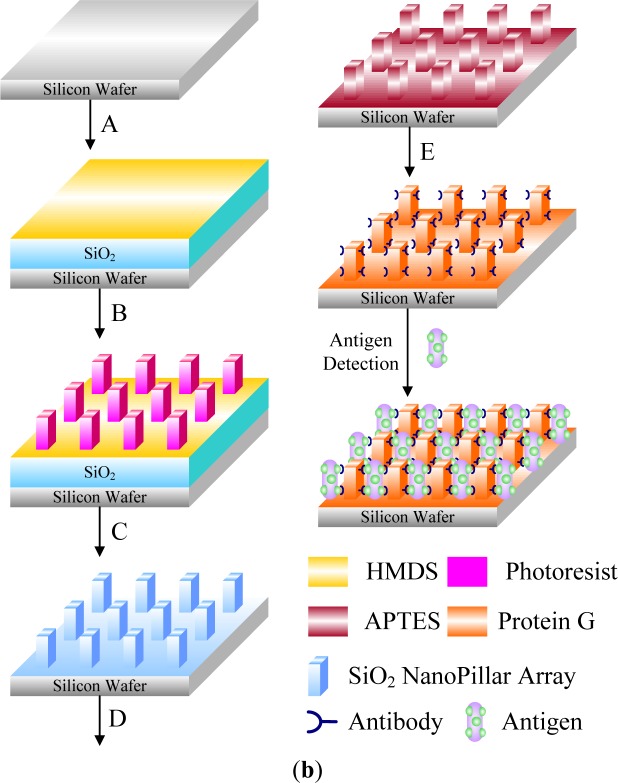
(**a**) Schematic representation of the process used to fabricate the MAHA-modified NPLA. (A) Silicon oxide film was deposited through plasma-enhanced chemical vapordeposition; the surface was then treated with HMDS in a thermal evaporator; (B) Negative photoresist was spun onto the HMDS-treated surface to pattern the photoresistas a 200-nm-scale NPLA; (C) Only the exposed regions of silicon dioxide were dry-etched by supplying a mixture gas of CHF3and CF4; the remaining photoresist hard maskwas then removed from the surface through the action of solvents; (D) The NPLA of silicon oxide was treated with APTES to assemble an array of amino groups; (E) The NPLApresenting amino groups was treated with a solution of EDC and NHS to immobilize proG on the pillar surfaces; MAHA units were oriented on the proG-modified pillarsurface; the presence of MHHA could be detected through ellipsometry [261]; (**b**) Values of *n*_eff_, measured using ellipsometry, of (A) bare 2DPRG; (B) proG-2DPRG; (C) MAHA-modified 2DPRG; (D) MAHA-modified 2DPRG after coupling with MHHA; and (E) MAHA-modified 2DPRG after binding BSA (control). Photographic images for (A)–(C) and (D) demonstrate the grating effect of the 2DPRG. Reprinted from Reference [262] with permission.

Immunological sensors (or immunosensors) are based on the recognition involved in the coupling of an antigen with an antibody, with immunoagents immobilized in a polymer matrix such as PVC or polyacrylamide gel. Either an immobilized antigen detects an antibody, or an immobilized antibody detects an antigen. Due to the interaction between an antibody and an antigen, a variation in electric charge, mass or optical properties [258] is detected directly with a variety of transducers. Holt *et al.* [259] reported the fabrication of a capillary immunosensor in poly(methylmethacrylate) for environmental monitoring and remediation, which can provide on-site, real-time, semiquantitative or quantitative measurement of contaminant levels. The military sector is in need of such devices for monitoring environmental levels of 2,4,6-trinitrotoluene (TNT) and hexahydro-1,3,5-trinitro-1,3,5-triazine (RDX) introduced into soils and water supplies and absorbed by plants, which are toxic to animal and human life. Since conventional continuous flow displacement immunosensors (CFI) constructed with fused-silica microcapillaries are fragile, rugged, tough and low density polymers, such as PMMA, PS or PE, are used after surface activation through alkoxysilane sol-gel technology for attaching the chemical or biochemical sensing elements. The key feature of the technique is the use of sol-gel technology to deposit a glass-like (Si· · ·OH) film on surfaces of the plastic capillary channels to facilitate antibody immobilization. These sensors exhibit sensitivity to low (mg/L) RDX concentrations and peak-to-peak signal variations that are generally less than 10% for multiple injections at a single RDX concentration. The useful lifetime of the coupons in experiments was greater than 10 h, even after multiple exposures to high (1000 mg/L) RDX levels. Temperature-regulated detachment of mammalian cells requires cell metabolic activity requiring ATP consumption, signal transduction, and cytoskeleton reorganization. Precoating PNIPAAm grafted surfaces with fibronectin improves the spreading of less adhesive cultured hepatocytes and reduces the temperature at which cultured cells are released from fibronectin-adsorbed grafted surfaces. Immunostaining with anti-fibronectin antibodies revealed that only fibronectin located beneath the cultured cells was removed from culture surfaces after the temperature was reduced. Fibronectin adsorbed to surface areas lacking direct cell attachment remained surface-bound after temperature reduction [219]. Principal component analysis using time-off light secondary ion mass spectrometry indicates that molecular ion fragments of amino acids are present on the surface after low-temperature liftoff from PNIPAAm brushes. Seeding new cells on surfaces from which an initial layer of cells has been removed indicates that liftoff dissociation leaves behind surfaces that better promote cell adhesion as compared to cell detachment by enzymatic detachment. It was concluded that the removal of bovine aortic endothelial cells via low-temperature liftoff from PNIPAAm brushes is less damaging to the extracellular matrix proteins remaining at the surface as compared to the enzymatic methods [260]. Combining 2DPRG pattern techniques, Chen *et al.* [261] reported the fabrication of a visualized immunosensor. Nanopillar arrays (NPLAs) of silicon oxide are fabricated for use as two-dimensional periodic relief gratings on silicon surfaces. (Figure 22a) Antibodies were successively oriented on the protein G-modified pillar surface, allowing the system to be used as an optical detector specific for the targeted antigen. The surfaces of the antibody-modified NPLAs underwent insignificant structural changes, but upon immunocapture of antigens, the NPLAs underwent dramatic changes in terms of their pillar scale. Binding of the antibodies to the NPLA occurred in a way that allowed them to retain their function and selectively bind the antigen. The binding of the antigen species to the NPLA resulted in a color change from pure green to pink, observable by the naked eye at an angle of 10°–20° (Figure 22b) [262]. Accordingly, these new films have potential applications as real-time optical biosensors.

#### 4.3.3. Chemical Sensors

Current chemical sensing technology has progressed to the point of encroaching upon the nanoscale, and this reduction in size has enabled the first generation of nanomechanical and chemical sensors that allow for detection of minute changes in the environmental conditions in real-time. Many of these sensors rely on measuring conformational changes in the polymer brush and to enhance the practicability of such sensors, so reliable ways have to be found to measure brush conformational changes. The construction and electrochemical response characteristics of poly(vinyl chloride) (PVC) membrane sensors were described by El-Ragehy *et al.* [263] for the determination of fluphenazine hydrochloride and nortriptyline hydrochloride. The method is based on the formation of ion-pair complexes between the two drug cations and sodium tetraphenylborate (NaTPB) or tetrakis (4-chlorophenyl) borate (KtpClPB). A novel plastic poly(vinyl chloride) membrane electrode based on pethidine-phosphotung state ion association as the electroactive material was developed by Liu *et al.* [264] for the determination of a pethidine hydrochloride drug in injections and tablets. Recent work by Tokareva *et al.* [38] explored an interesting transduction concept relying on measuring the localized surface plasmon resonance (SPR) emanating from gold nanoparticles chemically bonded to the surface of stimulus-responsive P2VP brushes. The resonance of transmission SPR (T-SPR) is highly sensitive to not only the environmental conditions but also the size, distribution, and shape of the nanoparticles. They illustrated the approach by observing a reversible 50 nm shift of the T-SPR absorption peak that correlated with the swelling response of P2VP brushes, induced by cycling the external pH between 5.0 and 2.0. The change in film thickness arose from the increase or decrease of electrostatic shielding between the polymer chains and the balance of an opposing osmotic pressure. The gold nano-islands present on the surface of the P2VP film allowed the detection of an absorption peak shift from 624 to 574 nm that corresponded to film shrinkage from ~4 to ~ nm. These results thus suggest that T-SPR may provide a useful tool for detecting changes in the solvent environment, including changes in pH, electrolyte concentration, or temperature.

The stimulus-response capability of polymer brushes could be used as a triggered “catch and release” surface for nanomaterial fabrication. For example, Comrie and Huck harnessed this capability for the fabrication of polymer-metal microobjects [265]. They incorporated a hydrophobic functional group into poly(glycidyl methacrylate) (PGMA) brushes as a means of creating a robust, etch-resistant film on a chromium substrate. The combination of etch resistance and ease of subsequent lift off allowed the fabrication of polymer-metal hybrid planar objects. Using this approach, it should thus be possible to trigger changes in surface properties and conformation, which would offer further control over the dimension and functionality of quasi-2D, micrometer sized objects. Comrie and Huck noted that the next steps would include an attempt to control the folding of planar microobjects into stable 3D objects that could find applications in drug delivery, biomimetic systems, and microelectromechanical systems. While stimuli-responsive hydrogel coated surfaces provide interesting substrates for novel device fabrication, the preferential chain orientation of brush thin films can also be used to template micro/nanoscale materials. For example, Tugulu *et al.* [266] report a novel templating approach for forming microstructured, crystalline calcite thin films. This approach makes use of photo-lithographically patterned poly-(methacrylic acid) (PMAA) brushes grown by SI-ATRP and capitalizes on the film’s ionic structure. When supersaturated calcium carbonate solution is passed over the PMMA brush, the brush templates the calcium carbonate through ionic interactions and, upon annealing, produces a crystalline calcite film that is an exact 3D reproduction of the patterned PMMA brush. The use of SRHs in this approach may bestow additional control over the template morphology by inducing compressive or lateral stresses that enable the control of nanofilm porosity or fine-tuning of fractal geometries. The use of SRHs for structural patterning and selective deposition techniques should prove an interesting development in the future.

**Figure 23.**
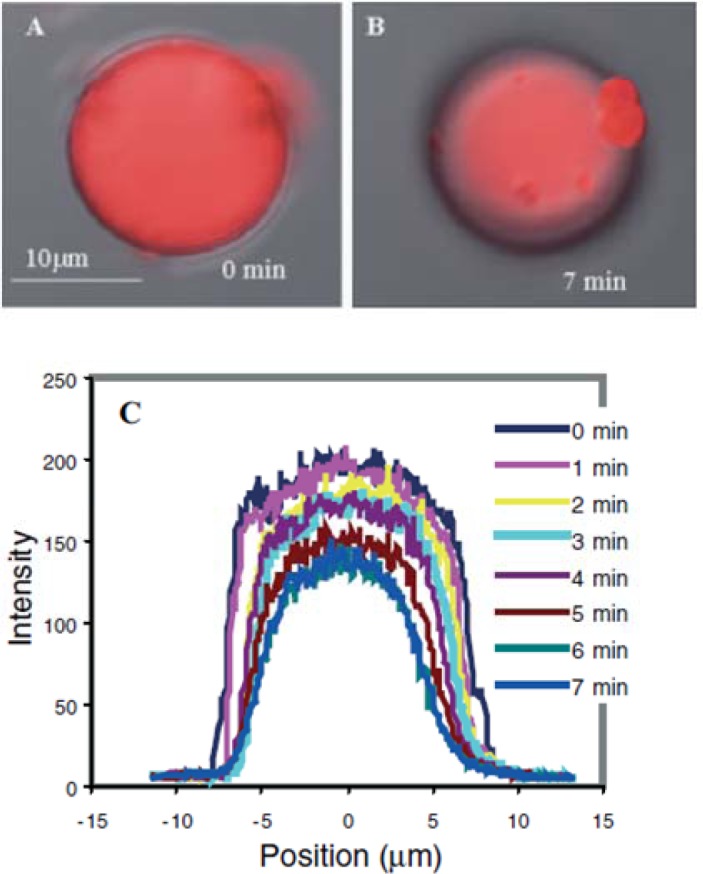
Confocal images demonstrating the release of rhodamine 6G from the polymer-grafted particles at 50 °C: (**A**) at *t* = 0 min; (**B**) at *t* = 7 min; (**C**) Fluorescence intensity line profiles taken from the horizontal midline of the image of the particle as a function of time. Reprinted from Reference [267] with permission.

The environmentally controlled change in macromolecule size from a compact hydrophobic globule to an expanded hydrophilic coil is exploited when SRHs are used in the systems with environmentally-controlled porosity, so called “chemical valves”. When a SRH is grafted to the surface of the pores in a porous membrane or chromatographic matrix, the transition in the macromolecule affects the total free volume of the pores available for the solvent and hence presents a means to regulate the porosity of the system (Figure 23) [267]. For example, grafting PNIPAAm on polypropylene microfiltration membranes using plasma- induced polymerization [268] or on polyethylene terephthalate and polypropylene membranes using radiation-induced polymerization [269] allows for the variation of water flux depending on the temperature. PNIPAAm has been grafted on both flat and hollow-fiber membranes [270]. Thermosensitive membranes have also been prepared by the phase inversion method from PNIPAAm-g-poly(vinylidene fluoride) [PNIPAAm-g-poly(VDF)] co-polymers [215] or by adding PNIPAAm [212] or thermosensitive elastin-like peptides [271] to tetraethyl orthosilicate solution prior to the sol-gel process. At temperatures below the LCST for the corresponding soluble polymers, the silica gel membranes are impermeable to all of the PEG markers, regardless of their molecular weight, whereas above the LCST, the membranes are permeable to PEG markers with molecular weights below a certain limit. Membranes with pH-responsive permeability are produced by grafting PMAA within the pores of porous polyethylene membranes [272], by grafting acrylic acid (AA) on the porous polypropylene membrane in supercritical carbon dioxide [273], as organic-inorganic composite prepared from tetra ethyl orthosilicate and chitosan [274], as self-organizing blends of (poly(VDF)) and an amphiphilic comb polymer having a poly(VDF) backbone and PMAA [275]. Polyelectrolyte multilayers with pH-responsive permeability have been assembled from poly(acrylic acid) (PAA), poly(allylamine hydrochloride) (PAH), and poly(sodium 4-styrenesulfonate) (SPS) in appropriate combinations [276], and from poly(allylamine hydrochloride) and sodium poly(styrene sulfonate) [277]. The membranes with dual, pH- and thermo-sensitivity are produced by co-grafting of NIPAAm and methacrylic acid (MAA) [278]; by UV-induced cross-linking co-polymerization of NIPAAm and AA deposited on the surface of cross-linked poly(2-hydroxyethyl methacrylate) matrix [279]; or by blending PNiPAAm with poly(VDF)-graftpoly(4-vinylpyridine) co-polymer [280]. The permeability and flux of aqueous solutions through the membranes prepared by grafting 4-vinylbenzyl chloride on poly(VDF) followed by modification with viologen can be reversibly regulated by redox reactions [281].

### 4.4. Actuators

**Figure 24.**
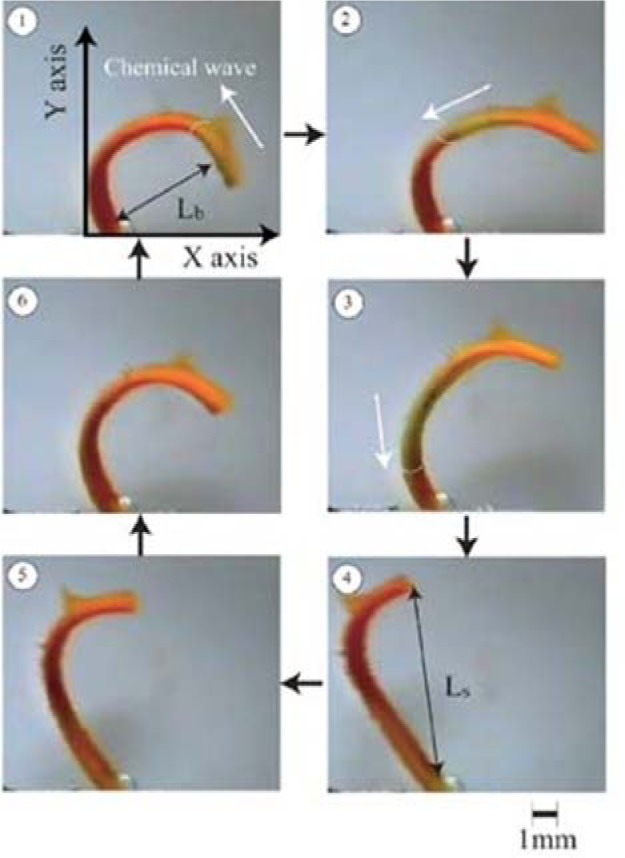
Images of the repeated bending and stretching motion of the poly[NIPAAm-co-Ru(bpy)3-co-AMPS] gel strip (R10-A3) in the mixture solution of the BZ substrates. First, the whole gel strip was homogeneous reduced state. Second, the gel strip is in a locally oxidized state when the chemical wave propagates in the gel from one edge to the other edge (1 → 4). Finally, the whole gel strip change reduced state, and was bending (5 → 6). Reprinted from Reference [284] with permission.

Applying an external signal changes the properties of stimuli-responsive hydrogels, while removal of the stimuli returns hydrogels to the initial state. It is thus necessary to change the stimuli repeatedly to achieve cyclical changes in hydrogel size. To achieve this goal, one has to apply the stimuli in a cyclical manner. Researchers have developed novel polymeric biomimetic gels with a self-oscillating function that generate periodic mechanical energy from the chemical energy of the Belousov–Zhabotinsky reaction. The self-oscillating gel may be useful in a number of applications such as pulse generators or chemical pacemakers, self-walking (auto-mobile) actuators, or micropumps [282]. The BZ reaction, which is well known as a non-equilibrium dissipative reaction, generates autonomous oscillations in the redox potential. For example, Yoshida fabricated gels exhibiting autonomous peristaltic motion without external stimuli. These gels were prepared by copolymerizing temperature-responsive N-isopropylacrylamide, with ruthenium tris(2,20-bipyridine) [Ru(bpy)3] as the catalyst for the BZ reaction, and used for directed particle transport [283]. BZ reaction results in local swelling of the hydrogel that changes over time. As result, a moving wave of swollen hydrogel is formed. The wave provides peristaltic motion of the adsorbed microparticles. In another approach, Maeda fabricated a self-oscillating gel actuator without external stimuli by producing a gradient structure; a pendulum motion is generated by fixing one edge of the gel (Figure 24) [284]. Aizenber and Sidorenko recently described a very fascinating approach for designing hydrogel-based actuators. They fabricated a thin hydrogel film with incorporated high-aspect ratio rods. Collapse or swelling of the thin hydrogel layer changed the orientation of the rods [285,286]. These hydrogel films were, in particular, used to design surfaces with “conventional” and “reverse” switching behavior. These surfaces reveal conventional switching—hydrophilic after exposure to water and hydrophobic after drying—if the rods are fixed on the surfaces. They can achieve reverse switching—hydrophobic after exposure to water and hydrophilic after drying—if the rods are not fixed. Swelling or collapse of the polymer layer changes the orientation of the nanorods and thus changes the wetting behavior. Due to the contraction of the polymer film upon drying, nanocolumns redirect the tensile forces from the gel into a lateral actuation that tilts the partially-exposed nanostructures. The tilt angle is controlled by the volume change of the gel and therefore can be regulated by the appropriate choice of the polymer. Re-hydration of the sample leads to swelling and relaxation of the hydrogel. This results in normal orientation of the bristles [287]. In fact, reversible swelling/deswelling of hydrogels is reminiscent to the contraction/relaxation of muscles. Lee *et al.* [288] have used this property of hydrogels and fabricated pH-sensitive hydrogel actuators mimicking the shape and motion of octopus and sperm. Such aquabots are able to produce directional motion in response to changes in electrochemical potential and could potentially be used for biomedical applications to sense and destroy certain microorganisms. These soft materials undergoing shape transformations in response to changes have great potential in applications such as tissue engineering, robotics and biosensing [289–291].

A micro-scale actuator that can regulate flow control in response to environmental stimuli was fabricated with stimuli responsive hydrogel valves inside microchannels [289]. For this actuator, micro-scale channels, fabricated by lithography, were filled with a photopolymerizable liquid consisting of acrylic acid and 2-hydroxyethyl methacrylate. The hydrogels in the microchannels performed sensing and actuation functions with pH changes of the local flow and with rapid response times of less than 10 s. Requiring no external power for operation, this device does not require complex assembly, overcoming significant disadvantages of conventional microfluidic actuators. This autonomously operating device, which responds to environmental change, could be advanced by providing the hydrogels in the microchannels with other types of functionality, such as temperature or glucose responsive properties.

**Figure 25.**
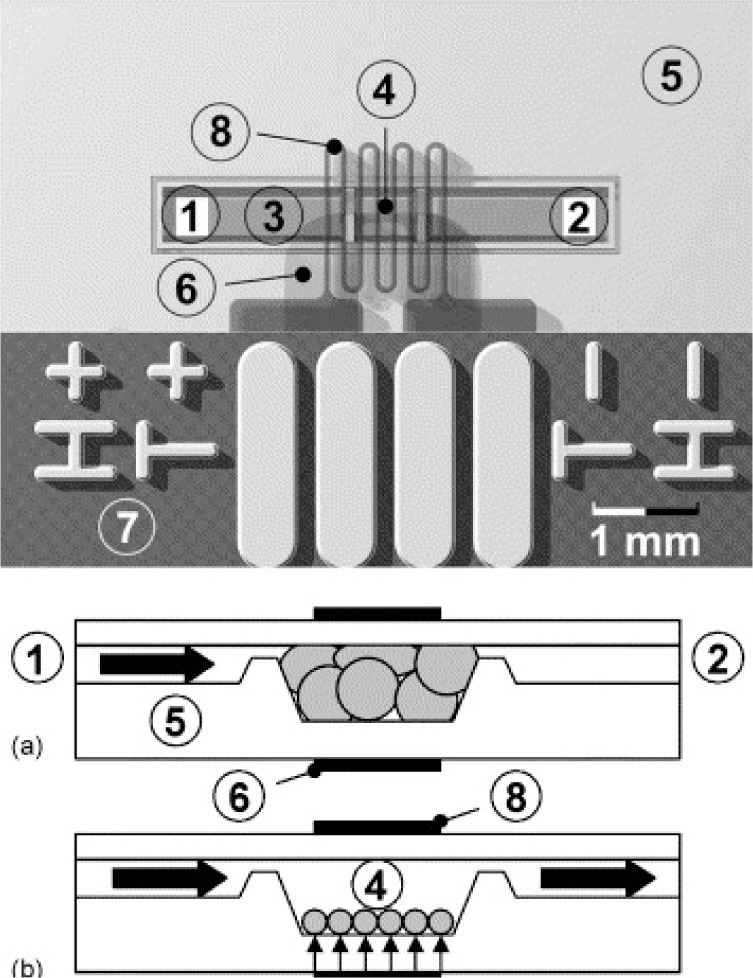
Schematic design of a hydrogel-based microvalve: 1, inlet; 2, outlet; 3, flow channel; 4, actuator chamber filled with hydrogel particles; 5, structure layer; 6, heating meander (located at backside); 7, circuit card; 8, temperature sensor (located at topside); (**a**) closed state at room temperature; (**b**) open state (gel actuator is heated above TC). Reprinted from Reference [199] with permission.

Conventional microactuators (using, for example, electromagnetic, electrostatic, or thermopneumatic effects) require external power for operation and are relatively complex assemblies, which limits their use in practical systems. Stimuli-responsive hydrogels have a significant advantage over conventional microfluidic actuators owing to their ability to undergo abrupt volume changes in response to the surrounding environment without the requirement of an external power source. Existing studies on responsive hydrogels in bulk suggest that these materials should be well suited to applications in microfluidics and actuator systems [290]. Thus, the use of responsive hydrogel materials to regulate flow eliminates the need for external power, external control, and complex fabrication schemes. These valves in the form of responsive hydrogels are incorporated or fabricated within the microfluidics channels and can shrink or swell in response to external stimuli, which in turn cause the channels to open or close, respectively. During the past decade, different fabrication systems of temperature-responsive hydrogel valves have been reported (Figure 25) [199,291]. Responsive hydrogels have also been incorporated in microfluidic devices as part of switchable supports. Separation schemes with dynamic temperature control are most likely to increase efficiency of high-throughput DNA analysis [292]. Responsive hydrogels have been evaluated as building materials for microfluidic systems using several criteria: (a) the ease of fabrication of the actuators; (b) the kinetics of the volume phase transition as a function of gel size and composition; (c) the ability of the actuators to block and displace the flows of different fluids; and (d) an isotropic swelling of the hydrogel and the responses to different stimuli [293]. The responses of a hydrogel actuator should occur within seconds for a microfluidics system, and this seems possible for actuators smaller than 100 mm. The rate of response can also be controlled by forming semi-interpenetrating networks (IPNs) with NiPAAm as the crosslinked component.

Apart from the temperature-responsive polymers, pH-responsive polymers have also been shown to have potential as actuators in microfluidics. Studies have been conducted on the mechanical properties of pH-sensitive hydrogels to produce optimal hydrogel valves and sensors [294]. pH-responsivehydrogels can be patterned in a microchannel by photopolymerization. The device uses a poly(hydroxyethyl methacrylate–acrylic acid) [poly(HEMA–AA)], pH-responsive hydrogel as the actuator in a PDMS microfluidic device [295]. A biomimetic check valve fabricated by *in situ* photopolymerization of poly (HEMA–AA) hydrogel inside a glass microchannel has been applied for directional control of fluid flow [296]. Combination of the different types of pH responsive hydrogels has been incorporated into flow sorters [297], and each behaves differently at the same pH. This property has been well utilized in a variety of applications.

## 5. Summary and Outlook

This review presents recent trends in the development of stimuli-responsive polymers (SRPs) by drawing on selected examples of recent research and describing how SRP and coatings are paving the way to advanced applications. It is clear that the design, synthesis and characterization of SRPs are rapidly evolving fields. In most approaches, polymers sensitive to environmental signal (pH, temperature and light) influenced were used. On the other hand, there are a growing number of publications reporting the design of systems sensitive to biochemical stimuli (mostly enzymes). Both environmentally- and biochemically-sensitive systems have been developed for designing actuators, sensors, and active elements in microfluidic devices, smart optical elements, and biomaterials. Owing to the rich variety of polymerization techniques, this contribution has covered the main features of controlled chain growth used in polymer brushes and further accomplishments regarding the control of the density, chain length, and nature of molecular brushes. Finally, the ability to create well-defined architectures with specific functional coatings opens new perspectives in materials science and provides new challenges for chemists’ imaginations.

Patterning polymer thin films into ordered structures is important for both scientific investigation and industrial needs. This issue encompasses the design of micro- and nano-patterned structures through varied lithographic techniques as well as the formation of polymers and block copolymers. Many approaches have been developed to pattern the polymer thin films, among which some need expensive equipment and/or toilsome efforts. Patterning by dewetting has proved to be an efficient and economic way to fabricate large-area, ordered structures. Using the dewetting process itself, like the movement of the three-phase line, simple patterns such as strips and dots (ordered or disordered) can be fabricated. Many ordered complex geometries have been achieved using controlled dewetting processes involving one or more of the techniques with chemically and physically modified substrates, physical confinement, or topographic structures on film surfaces. Novel, smaller, and more complex patterns can be achieved by combining dewetting with other techniques and new materials.

A central goal of engineering on the nanoscale is to produce assembled materials that are ordered over a range of length scales and in which molecular characteristics control larger-scale structural and physico-chemical properties. An important, new application for SRPs lies in the nascent field of hierarchical nano- and microfabrication, where SRPBs are establishing themselves as important manipulation tools. To date, applications of SRPs have mostly been restricted to controlling surface wettability and to prototypical sensor applications, often related to the biomedical arena. SRPs also provide unique opportunities to transduce stimulus-induced, structural changes into mechanical stresses that can be harnessed for mechanical actuation and sensing. For all these applications, the design and synthesis of multifunctional, and hybrid biomolecular SRPs promise exciting advances. Furthermore, the use of SRPs in combination with local sensing techniques, such as surface plasmon resonance, charts new territory for highly sensitive detection devices that harness the large conformational changes of SRPs for signal transduction. If the field of SRP is to continue to grow and to make inroads towards commercial applications, scientists must now shift their focus from prototypical demonstrations in the laboratory to true application development. While this in no way implies a need to cease exploratory research, there is a growing need to explore robust and reproducible routes to synthesize surface-grafted, stimulus-responsive polymer brushes on a variety of commercially important substrates and to develop an understanding of the long-term stability and the failure conditions for these SRP films. Gauging from the creative and active research in the rapidly growing field of SRPs, many of the current challenges that impede the full exploitation and limit the potential of complex stimulus-responsive polymer brushes will soon be overcome.

## References

[1] Deshmukh P.K., Ramani K.P., Singh S.S., Tekade A.R., Chatap V.K., Patil G.B., Bari S.B. (2013). Stimuli-sensitive layer-by-layer (LbL) self-assembly systems: Targeting and biosensory applications. J. Control. Release.

[2] Kikuchi A., Okano T. (2002). Intelligent thermoresponsive polymeric stationary phases for aqueous chromatography of biological compounds. Prog. Polym. Sci.

[3] Ware T., Simon D., Rennaker R.L., Voit W. (2013). Smart polymers for neural interfaces. Polym. Rev.

[4] Hamner K.L., Alexander C.M., Coopersmith K., Reishofer D., Provenza C., Maye M.M. (2013). Using temperature-sensitive smart polymers to regulate DNA-mediated nanoassembly and encoded nanocarrier drug release. ACS Nano.

[5] Qiu Y., Park K. (2001). Environment-sensitive hydrogels for drug delivery. Adv. Drug Deliv. Rev.

[6] Reinhardt M., Dzubiella J., Trapp M., Gutfreund P., Kreuzer M., Groschel A.H., Muller A.H.E., Ballauff M., Steitz R. (2013). Fine-tuning the structure of stimuli-responsive polymer films by hydrostatic pressure and temperature. Macromolecules.

[7] Wang C., Kang Y.T., Liu K., Li Z.B., Wang Z.Q., Zhang X. (2012). pH and enzymatic double-stimuli responsive multi-compartment micelles from supra-amphiphilic polymers. Polym. Chem.

[8] Kaneider N.C., Dunzendorfer S., Wiedermann C.J. (2004). Heparan sulfate proteoglycans are involved in opiate receptor-mediated cell migration. Biochemistry.

[9] Meng H., Mohamadian H., Stubblefield M., Jerro D., Ibekwe S., Pang S.S., Li G.Q. (2013). Various shape memory effects of stimuli-responsive shape memory polymers. Smart Mater. Struct.

[10] Zhang M., Estournes C., Bietsch W., Mueller A.H.E. (2004). Superparamagnetic hybrid nanocylinders. Adv. Funct. Mater.

[11] Fujiwara N., Asaka K., Nishimura Y., Oguro K., Torikai E. (2000). Preparation of gold-solid polymer electrolyte composites as electric stimuli-responsive materials. Chem. Mater.

[12] Cheng G., Boeker A., Zhang M., Krausch G., Mueller A.H.E. (2001). Amphiphilic cylindrical core-shell brushes via a “grafting from” process using ATRP. Macromolecules.

[13] Xia Y., Yin X., Burke N.A.D., Stoever H.D.H. (2005). Thermal response of narrowdisperse poly(N-isopropylacrylamide) prepared by atom transfer radical polymerization. Macromolecules.

[14] Rapoport N. (2007). Physical stimuli-responsive polymeric micelles for anti-cancer drug delivery. Prog. Polym. Sci.

[15] Chen J.K., Wang J.H., Fan S.K., Chang J.Y. (2012). Reversible hydrophobic/hydrophilic adhesive of PS-b-PNIPAAm copolymer brush nanopillar arrays for mimicking the climbing aptitude of geckos. J. Phys. Chem. C.

[16] Oh J.K., Drumright R., Siegwart D.J., Matyjaszewski K. (2008). The development of microgels/nanogels for drug delivery applications. Prog. Polym. Sci.

[17] Beija M., Marty J.D., Destarac M. (2011). RAFT/MADIX polymers for the preparation of polymer/inorganic nanohybrids. Prog. Polym. Sci.

[18] Delcea M., Möhwald H., Skirtach A.G. (2011). Stimuli-responsive LbL capsules and nanoshells for drug delivery. Adv. Drug Deliv. Rev.

[19] Uhlig K., Boysen B., Lankenau A., Jaeger M., Wischerhoff E., Lutz J.-F., Laschewsky A., Duschl C. (2012). On the influence of the architecture of poly(ethylene glycol)-based thermoresponsive polymers on cell adhesion. Biomicrofluidics.

[20] Skirtach A.G., Yashchenok A.M., Möhwalda H. (2011). Encapsulation, release and applications of LbL polyelectrolyte multilayer capsules. Chem. Commun.

[21] Bedard M., Skirtach A.G., Sukhorukov G.B. (2007). Optically induced encapsulation. Macromol. Rapid Commun.

[22] Honda M., Kataoka K., Seki T., Takeoka Y. (2009). Confined stimuli-responsive polymer gel in inverse opal polymer membrane for colorimetric glucose sensor. Langmuir.

[23] Fleischmann E.K., Zentel R. (2013). Liquid-crystalline ordering as a concept in materials science: From semiconductors to stimuli-responsive devices. Angew. Chem. Int. Ed.

[24] Fukumori K., Akiyama Y., Kumashiro Y., Kobayashi J., Yamato M., Sakai K., Okano T. (2010). Characterization of ultra-thin temperature-responsive polymer layer and its polymer thickness dependency on cell attachment/detachment properties. Macromol. Biosci.

[25] Bellin I., Kelch S., Langer R., Lendlein A. (2006). Polymeric triple shape materials. Proc. Natl. Acad. Sci. USA.

[26] Schafer C.G., Gallei M., Zahn J.T., Engelhardt J., Hellmann G.P., Rehahn M. (2013). Reversible light-, thermo-, and mechano-responsive elastomeric polymer opal films. Chem. Mater.

[27] Seuring J., Agarwal S. (2012). Polymers with upper critical solution temperature in aqueous solution. Macromol. Rapid Commun.

[28] Chang K., Rubright N.C., Lowery P.D., Taite L.J. (2013). Structural optimization of highly branched thermally responsive polymers as a means of controlling transition temperature. J. Polym. Sci. A Polym. Chem.

[29] Heskins M., Guillet J.E. (1968). Solution properties of poly (N-isopropylacrylamide). J. Macromol. Sci. Chem.

[30] Suwa K., Morishita K., Kishida A., Akashi M. (1997). Synthesis and functionalities of poly(N-vinylalkylamide). V. Control of a lower critical solution temperature of poly (N-vinylalkylamide). J. Polym. Sci. A Polym. Chem.

[31] Schild H.G. (1992). Poly(N-isopropylacrylamide): Experiment theory and application. Prog. Polym. Sci.

[32] Okubo M., Ahmad H., Suzuki T. (1998). Synthesis of temperaturesensitive micron-sized monodispersed composite polymer particles and its application as a carrier for biomolecules. Colloid. Polym. Sci.

[33] Aoyagi T., Ebara M., Sakai K., Sakurai Y., Okano T. (2000). Novel bifunctional polymer with reactivity and temperature sensitivity. J. Biomater. Sci. Polym. Ed.

[34] Ebara M., Aoyagi T., Sakai K., Okano T. (2000). Introducing reactive carboxyl side chains retains phase transition temperature Sensitivity in N-isopropylacrylamide copolymer gels. Macromolecules.

[35] Cheng N., Brown A.A., Azzaroni O., Huck W.T.S. (2008). Thickness-dependent properties of polyzwitterionic brushes. Macromolecules.

[36] Azzaroni O., Brown A.A., Huck W.T.S. (2006). UCST wetting transitions of polyzwitterionic brushes driven by self-association. Angew. Chem. Int. Ed.

[37] Tu H., Heitzman C.E., Braun P.V. (2004). Patterned poly(Nisopropylacrylamide) brushes on silica surfaces by microcontact printing followed by surface-initiated polymerization. Langmuir.

[38] Tokareva I., Minko S., Fendler J.H., Hutter E. (2004). Nanosensors based on responsive polymer brushes and gold nanoparticle enhanced transmission surface plasmon resonance spectroscopy. J. Am. Chem. Soc.

[39] Aoki T., Muramatsu M., Torii T., Sanui K., Ogata N. (2001). Thermosensitive phase transition of an optically active polymer in aqueous milieu. Macromolecules.

[40] Gan L.H., Roshan Deen G., Loh X.J., Gan Y.Y. (2001). New stimuliresponsive copolymers of N-acryloyl-N0-alkyl piperazine and methyl methacrylate and their hydrogels. Polymer.

[41] Suwa K., Yamamoto K., Akashi M., Takano K., Tanaka N., Kunugi S. (1998). Effects of salt on the temperature and pressure responsive properties of poly(N-vinylisobutyramide) aqueous solutions. Colloid. Polym. Sci.

[42] Maeda Y. (2001). IR spectroscopic study on the hydration and the phase transition of poly(vinyl methyl ether) in water. Langmuir.

[43] Inoue T. (1997). Temperature sensitivity of a hydrogel network containing different LCST oligomers grafted to the hydrogel backbone. Polym. Gels Netw.

[44] Sun T.L., Wang G.J., Feng L., Liu B.Q., Ma Y.M., Jiang L. (2004). Reversible switching between superhydrophilicity and superhydrophobicity. Angew. Chem. Int. Ed.

[45] Fu Q., Rao G.V.R., Basame S.B., Keller D.J., Artyushkova K., Fulghum J.E. (2004). Reversible control of free energy and topography of nanostructured surfaces. J. Am. Chem. Soc.

[46] Luzinov I., Minko S., Tsukruk V.V. (2008). Responsive brush layers: From tailored gradients to reversibly assembled nanoparticles. Soft Matter.

[47] Shibayama M., Tanaka T. (1993). Volume phase transition and related phenomena of polymer gels. Adv. Polym. Sci.

[48] Chen G., Hoffman A.S. (1995). Graft copolymers that exhibit temperature-induced phase transition over a wide range of pH. Nature.

[49] Hoffman A.S. (2000). Really smart bioconjugates of smart polymers and receptor proteins. J. Biomed. Mater. Res.

[50] Costa E., Coelho M., Ilharco L.M., Aguiar-Ricardo A., Hammond P.T. (2011). Tannic acid mediated suppression of PNIPAAm microgels thermoresponsive behavior. Macromolecules.

[51] Yang H.W., Chena J.K., Cheng C.C., Kuo S.W. (2013). Association of poly(N-isopropylacrylamide) containing nucleobase multiple hydrogen bonding of adenine for DNA recognition. Appl. Surf. Sci.

[52] Stubenrauch K., Voets I., Fritz-Popovski G., Trimmel G. (2009). PH and ionic strength responsive polyelectrolyte block copolymer micelles prepared by ring opening metathesis polymerization. J. Polym. Sci. A Polym. Chem.

[53] Dupin D., Rosselgong J., Armes S.P., Routh A.F. (2007). Swelling kinetics for a pH-induced latex-to-microgel transition. Langmuir.

[54] Philippova O.E., Hourdet D., Audebert R., Khokhlov A.R. (1997). pH-responsive gels of hydrophobically modified poly(acrylic acid). Macromolecules.

[55] Torres-Lugo M., Peppas N.A. (1999). Molecular design and *in vitro* studies of novel pH-sensitive hydrogels for the oral delivery of calcitonin. Macromolecules.

[56] Tonge S.R., Tighe B.J. (2001). Responsive hydrophobically associating polymers: A review of structure and properties. Adv. Drug Deliv. Rev.

[57] Murthy N., Robichaud J.R., Tirrell D.A., Stayton P.S., Hoffman A.S. (1999). The design and synthesis of polymers for eukaryotic membrane disruption. J. Control Release.

[58] Lee A.S., Butun V., Vamvakaki M., Armes S.P., Pople J.A., Gast A.P. (2002). Structure of pH-dependent block copolymer micelles: charge and ionic strength dependence. Macromolecules.

[59] Tantavichet N., Pritzker M.D., Burns C.M. (2001). Proton uptake by poly(2-vinylpyridine) coatings. J. Appl. Polym. Sci.

[60] Ferruti P., Barbucci R. (1984). Linear amino polymers: Synthesis protonation and complex formation. Adv. Polym. Sci.

[61] Franck-Lacaze L., Sistat P., Huguet P. (2009). Determination of the pK(a) of poly (4-vinylpyridine)-based weak anion exchange membranes for the investigation of the side proton leakage. J. Membr. Sci.

[62] Gohy J., Lohmeijer B.G.G., Varshney S.K., Decamps B., Leroy E., Boileau S., Schubert U.S. (2002). Stimuli-responsive aqueous micelles from an ABC metallo-supramolecular triblock copolymer. Macromolecules.

[63] Sutton R.C., Thai L., Hewitt J.M., Voycheckand C.L., Tan J.S. (1988). Microdomain characterization of styrene–imidazole copolymers. Macromolecules.

[64] Sideratou Z., Tsiourvas D., Paleos C.M. (2000). Quaternized poly(propylene imine) dendrimers as novel pH-sensitive vontrolled-release systems. Langmuir.

[65] Lee E., Na K., Bae Y. (2005). Super pH-sensitive multifunctional polymeric micelle. Nano Lett.

[66] Xu Y., Bolisetty S., Drechsler M., Fang B., Yuan J. (2008). pH and salt responsive poly(N,N-dimethylaminoethyl methacrylate) cylindrical brushes and their quaternized derivatives. Polymer.

[67] Azzaroni O., Brown A.A., Huck W.T.S. (2007). Tunable wettability by clicking into polyelectrolyte brushes. Adv. Mater.

[68] Lim H.S., Kwak D., Lee D.Y., Lee S.G., Cho K. (2007). UV-Driven reversible switching of a roselike vanadium oxidefilmbetween superhydrophobicity and superhydrophilicity. J. Am. Chem. Soc.

[69] Finkelmann H., Nishikawa E. (2001). A new opto-mechanical effect in solids. Phys. Rev. Lett.

[70] Andreopoulos F.M., Beckman E.J., Russell A.J. (1998). Lightinduced tailoring of PEG-hydrogel properties. Biomaterials.

[71] Scott T.F., Draughon R.B., Bowman C.N. (2006). Actuation in crosslinked polymers via photoinduced stress relaxation. Adv. Mater.

[72] White T.J., Tabiryan N., Tondiglia V.P., Serak S., Hrozhyk V., Vaia R.A., Bunning T.J. (2008). High frequency photodriven polymer oscillator. Soft Matter.

[73] Yamada M., Kondo M., Miyasato R., Naka Y., Mamiya J.I., Kinoshita M., Shishido A., Yu Y., Barrett C.J., Iked T. (2009). Photomobile polymer materials—Various three-dimensional movements. J. Mater. Chem.

[74] Katsonis N., Lubomska M., Pollard M.M., Feringa B.L., Rudolf P. (2007). Synthetic light-activated molecular switches and motors on surfaces. Prog. Surf. Sci.

[75] Wan P.B., Jiang Y.G., Wang Y.P., Wang Z.Q., Zhang X. (2008). Tuning surface wettability through photocontrolled reversible molecular shuttle. Chem. Commun.

[76] Behrendt R., Renner C., Schenk M., Wang F.Q., Wachtveitl J., Oesterhelt D. (1999). Photomodulation of the conformation of cyclic peptides with azobenzene moieties in the peptide backbone. Angew. Chem. Int. Ed.

[77] Bunker B.C., Kim B.I., Houston J.E., Rosario R., Garcia A.A., Hayes M. (2003). Direct observation of photo switching in tethered spiropyrans using the interfacial force microscope. Nano Lett.

[78] Fries K., Samanta S., Orski S., Locklin J. (2008). Reversible colorimetric ion sensors based on surface initiated polymerization of photochromic polymers. Chem. Commun.

[79] Xia F., Zhu Y., Feng L., Jiang L. (2009). Smart responsive surfaces switching reversibly between super-hydrophobicity and superhydrophilicity. Soft Matter.

[80] Rosario R., Gust D., Hayes M., Jahnke F., Springer J., Garcia A.A. (2002). Photon-modulated wettability changes on spiropyran-coated surfaces. Langmuir.

[81] Athanassiou A., Lygeraki M.I., Pisignano D., Lakiotaki K., Varda M., Mele E. (2006). Photocontrolled variations in the wetting capability of photochromic polymers enhanced by surface nanostructuring. Langmuir.

[82] Yu Y., Ikeda T. (2005). photodeformable polymers: A new kind of promising smart material for micro- and nano-applications. Macromol. Chem. Phys.

[83] Pei H., Li W., Liu Y., Wang D., Wang J., Shi J., Cao S. (2012). Ring-opening metathesis polymerization of norbornene derivatives for multifunctionalized all-optical photorefractive polymers with a non-conjugated main chain. Polymer.

[84] Chang C.J., Whang W.T., Hsu C.C., Ding Z.Y., Hsu K.Y., Lin S.H. (1999). Synthesis and relationships between the nonlinear optical and holographic properties of dual functional azocarbazole chromophores based on photorefractive polymers. Macromolecules.

[85] Chang C.J., Whang W.T., Hsu K.Y., Hsieh M.L. (1999). Effect of the sensitizer on the properties of fully functionalized photorefractive epoxy polymers and their performance in the hologram image storage. J. Polym. Sci. Polym. Phys.

[86] Chang C.J., Whang W.T., Hsu K.Y. (1999). Chain flexibility effect on the refractive index grating of fully functionalized carbazole/DO3/epoxy polymer. J. Appl. Polym. Sci.

[87] Chang C.J., Wang H.C., Liao G.Y., Whang W.T., Liu J.M., Hsu K.Y. (1997). Effect of laser wavelength on the photorefractive characteristics of PMDA-DR19 based photorefractive polymeric materials. Polymer.

[88] Chang C.J., Whang W.T. (1997). Trap characteristics study of photorefractive polymer materials by thermal stimulated current spectroscopy. J. Polym. Res.

[89] Yu X., Zhou S., Zheng X., Guo T., Xiao Y., Song B. (2009). A biodegradable shape-memory nanocomposite with excellent magnetism sensitivity. Nanotechnology.

[90] Cuevas J.M., Alonso J., German L., Iturrondobeitia M., Laza J.M., Vilas J.L. (2009). Magneto-active shape memory composites by incorporating ferromagnetic microparticles in a thermo-responsive polyalkenamer. Smart Mater. Struct.

[91] Golbang A., Kokabi M. (2011). Temporary shape development in shape memory nanocomposites using magnetic force. Eur. Polym. J.

[92] Zeng M., Or S.W., Chan H.L.W. (2010). Giant resonance frequency tunable magnetoelectric effect in a device of PZT drum transducer, NdFeB and Fe-core solenoid. J. Appl. Phys.

[93] Zhang D.W., Liu Y.J., Leng J.S. (2010). Study on the activation of styrene-based shape memory polymer by medium-infrared laser light. Appl. Phys. Lett.

[94] Varga Z., Filipcsei G., Zrinyi M. (2006). Magnetic field sensitive functional elastomers with tuneable elastic modulus. Polymer.

[95] Nikitin L.V., Stepanov G.V., Mironova L.S., Gorbunov A.I. (2004). Magnetodeformational effect and effect of shape memory in magnetoelastics. J. Magn. Magn. Mater.

[96] Benkoski J.J., Bowles S.E., Jones R.L., Douglas J.F., Pyun J., Karim A. (2008). Self-assembly of polymer-coated ferromagnetic nanoparticles into mesoscopic polymer chains. J. Polym. Sci. Polym. Phys.

[97] Alves K.G.B., Andrade C.A.S., Campello S.L., de Souza R.E., de Melo C.P. (2013). Magnetite/polypyrrole hybrid nanocomposites as a promising magnetic resonance imaging contrast material. J. Appl. Polym. Sci.

[98] Zhang J.L., Srivastava R.S., Misra R.D.K. (2007). Core-shell magnetite nanoparticles surface encapsulated with smart stimuli-responsive polymer: Synthesis, characterization, and LCST of viable drug-targeting delivery system. Langmuir.

[99] Bar-Cohen Y. (2007). Editorial: Focus issue on biomimetics using electroactive polymers as artificial muscles. Bioinspir. Biomim.

[100] Le H.H., Kolesov I., Ali Z., Uthardt M., Osazuwa O., Ilisch S. (2010). Effect of filler dispersion degree on the Joule heating stimulated recovery behaviour of nanocomposites. J. Mater. Sci.

[101] Leng J., Lv H., Liu Y., Du S. (2007). Electroactivate shape-memory polymer filled with nanocarbon particles and short carbon fibers. Appl. Phys. Lett.

[102] Cho J.W., Kim J.W., Jung Y.C., Goo N.S. (2005). Electroactive shape-memory polyurethane composites incorporating carbon nanotubes. Macromol. Rapid Commun.

[103] Fei G., Li G., Wu L., Xi H. (2012). A spatially and temporally controlled shape memory process for electrically conductive polymer-carbon nanotube composites. Soft Matter.

[104] Jung Y.C., Yoo H.J., Kim Y.A., Cho J.W., Endo M. (2010). Electroactive shape memory performance of polyurethane composite having homogeneously dispersed and covalently crosslinked carbon nanotubes. Carbon.

[105] Zhang Q.M., Li H., Poh M., Xu H., Cheng Z.Y., Xia F., Huang C. (2002). An all-organic composite actuator material with a high dielectric constant. Nature.

[106] Huang C., Zhang Q. (2004). Enhanced dielectric and electromechanical responses in high dielectric constant all-polymer percolative composites. Adv. Funct. Mater.

[107] Bobnar V., Levstik A., Huang C., Zhang Q.M. (2007). Enhanced dielectric response in all-organic polyaniline-poly(vinylidene fluoride-trifluoroethylene-chlorotrifluoroethylene) composite. J. Noncryst. Solids.

[108] Chen J.K., Hsieh C.Y., Huang C.F., Li P.M., Kuo S.W., Chang F.C. (2008). Using solvent immersion to fabricate variably patterned poly(methyl methacrylate) brushes on silicon surfaces. Macromolecules.

[109] Chen J.K., Hsieh C.Y., Huang C.F., Li P.M. (2009). Characterization of patterned poly(methyl methacrylate) brushes under various structures upon solvent immersion. J. Colloid Interface Sci.

[110] Chen J.K., Zhuang A.L. (2010). Fabrication of a highly dense line patterned polystyrene brush on silicon surfaces using very large scale integration processing. J. Phys. Chem. C.

[111] Chen J.K., Zhuang A.L. (2011). Patterning nanocluster polystyrene brushes grafted from initiator cores on silicon surfaces by lithography processing. Colloid Polym. Sci.

[112] Tagit O., Tomczak N., Benetti E.M., Cesa Y., Blum C., Subramaniam V., Herek J.L., Vancso G.J. (2009). Temperature-modulated quenching of quantum dots covalently coupled to chain ends of poly(N-isopropyl acrylamide) brushes on gold. Nanotechnology.

[113] Yu K., Wang H.F., Han Y.C. (2007). Motion of integrated CdS nanoparticles by phase separation of block copolymer brushes. Langmuir.

[114] Chen J.K., Pai P.C., Chang J.Y., Fan S.K. (2012). PH-responsive one-dimensional periodic relief grating of polymer brush-gold nanoassemblies on silicon surface. ACS Appl. Mater. Interface.

[115] Tokareva I., Tokarev I., Minko S., Hutter E., Fendler J.H. (2006). Ultrathin molecularly imprinted polymer sensors employing enhanced transmission surface plasmon resonance spectroscopy. Chem. Commun.

[116] Sheparovych R., Motornov M., Minko S. (2009). Low adhesive surfaces that adapt to changing environments. Adv. Mater.

[117] Gallyamov M.O., Tartsch B., Khokhlov A.R., Sheiko S.S., Boerner H.G., Matyjaszewski K. (2004). Real-time scanning force microscopy of macromolecular conformational transitions. Macromol. Rapid Commun.

[118] Gallyamov M.O., Tartsch B., Mela P., Börner H., Matyjaszewski K., Sheiko S.S. (2007). A scanning force microscopy study on the motion of single brush-like macromolecules on a silicon substrate induced by coadsorption of small molecules. Phys. Chem. Chem. Phys.

[119] Chen T.Y., Chen J.K. (2011). Ferritin immobilization on patterned poly(2-hydroxyethyl methacrylate) brushes on silicon surfaces from colloid system. Colloid. Polym. Sci.

[120] Chen J.K., Chen T.Y. (2011). Fabrication of high-aspect-ratio poly(2-hydroxyethyl methacrylate) brushes patterned on silica surfaces by very-large-scale integration process. J. Colloid Interface Sci.

[121] Mathieu M., Friebe A., Franzka S., Ulbricht M., Hartmann N. (2009). Surface-initiated polymerization on laser-patterned templates: morphological scaling of nanoconfined polymer brushes. Langmuir.

[122] Prucker O., Schimmel M., Tovar G., Knoll W., Rühe J. (1998). Microstructuring of molecularly thin polymer layers by photolithography. Adv. Mater.

[123] Husemann M., Morrison M., Benoit D., Frommer J., Mate C.M., Hinsberg W.D., Hedrick J.L., Hawker C.J. (2000). Manipulation of surface properties by patterning of covalently bound polymer brushes. J. Am. Chem. Soc.

[124] Fan X., Lin L., Dalsin J.L., Messersmith P.B. (2005). Biomimetic anchor for surface-initiated polymerization from metal substrates. J. Am. Chem. Soc.

[125] Zhou F., Jiang L., Liu W., Xue Q. (2004). Fabrication of chemically tethered binary polymer-brush pattern through two-step surface-initiated atomic-transfer radical polymerization. Macromol. Rapid Commun.

[126] Zharnikov M., Grunze M. (2002). Modification of thiol-derived selfassembling monolayers by electron and X-Ray irradiation: Scientific and lithographic aspects. J. Vac. Sci. Technol. B.

[127] Chen J.K., Ko F.H., Chang F.C. (2005). Structral transformation of acrylic resin upon controlled electron-beam exposure yield positive and negative resists. Adv. Funct. Mater.

[128] Schmelmer U., Jordan R., Geyer W., Eck W., Gölzhäuser A., Grunze M., Ulman A. (2003). Surface-initiated polymerization on self-assembled monolayers: Amplification of patterns on the micrometer and nanometer scale. Angew. Chem. Int. Ed.

[129] He Q., Kueller A., Schilp S., Leisten F., Kolb H.A., Grunze M., Li J. (2007). Fabrication of controlled thermosensitive polymer nanopatterns with one-pot polymerization through chemical lithography. Small.

[130] Ballav N., Schilp S., Zharnikov M. (2008). Electron-beam chemical lithography with aliphatic self-assembled monolayers. Angew. Chem. Int. Ed.

[131] Vieu C., Carcenac F., Pepin A., Chen Y., Mejias M., Lebib A., Manin-Ferlazzo L., Couraud L., Launois H. (2000). Electron beam lithography: Resolution limits and applications. Appl. Surf. Sci.

[132] Ahn S.J., Kaholek M., Lee W.K., Lamattina B., LaBean T.H., Zauscher S. (2004). Surface-initiated polymerization on nanopatterns fabricated by electron-beam lithography. Adv. Mater.

[133] Jonas A.M., Hu Z., Glinel K., Huck W.T.S. (2008). Effect of nanoconfinement on the collapse transition of responsive polymer brushes. Nano Lett.

[134] Nie Z., Kumacheva E. (2008). Patterning surface with functional polymers. Nature.

[135] Piner R.D., Zhu J., Xu F., Hong S., Mirkin C.A. (1999). Dip-pen nanolithography. Science.

[136] Ginger D.S., Zhang H., Mirkin C.A. (2004). The evolution of dip-pen nanolithography. Angew. Chem. Int. Ed.

[137] Liu X., Guo S., Mirkin C.A. (2003). Surface and site-specific ring-opening metathesis polymerization initiated by dip-pen nanolithography. Angew. Chem. Int. Ed.

[138] Ma H., Hyun J., Stiller P., Chilikoti A. (2004). Non-fouling oligo(ethylene glycol) functionnalized polymer brushes synthesized by surface initiated atom transfer polymerization. Adv. Mater.

[139] Zapotoczny S., Benetti E.M., Vancso G.J. (2007). Preparation and characterization of macromolecular “hedge” brushes grafted from Au nanowires. J. Mater. Chem.

[140] Kaholek M., Lee W.K., LaMattina B., Caster K.C., Zauscher S. (2004). Fabrication of stimulus-responsive nanopatterned polymer brushes by scanning-probe lithography. Nano Lett.

[141] Kaholek M., Lee W.K., Ahn S.J., Ma H., Caster K.C., LaMattina B., Zauscher S. (2004). Stimulus-responsive poly(N-isopropylacrylamide) brushes and nanopatterns prepared by surface-initiated polymerization. Chem. Mater.

[142] Liu X., Li Y., Zheng Z. (2010). Programming nanostructures of polymer brushes by dip-pen nanodisplacement lithography (DNL). Nanoscale.

[143] Morsch S., Schofield W.C.E., Badyal J.P.S. (2010). Surface actuation of smart nanoshutters. Langmuir.

[144] Chen C.F., Tzeng S.D., Lin M.H., Gwo S. (2006). Electrostatic assembly of gold colloidal nanoparticles on organosilane monolayers patterned by microcontact electrochemical conversion. Langmuir.

[145] Lee W.K., Caster K.C., Kim J., Zauscher S. (2006). Nanopatterned polymer brushes by combining AFM anodization lithography with ringopening metathesis polymerization in the liquid and vapor phase. Small.

[146] Benetti E.M., Chung H.J., Vancso G.J. (2009). pH responsive polymeric brush nanostructures: Preparation and characterization by scanning probe oxidation and surface initiated polymerization. Macromol. Rapid Commun.

[147] Becer C.R., Haensch C., Hoeppener S., Schubert U.S. (2007). Patterned polymer brushes grafted from bromine-functionalized, chemically active surface templates. Small.

[148] Kim E., Xia Y., Whitesides G.M. (1995). Making polymeric microstuctures: Capillary micromolding. Nature.

[149] Zhao X., Xia Y., Whitesides G.M. (1996). Fabrication of threedimensional micro-structures: Microtransfer molding. Adv. Mater.

[150] Perl A., Reinhoudt D.N., Huskens J. (2009). Microcontact printing: Limitations and achievements. Adv. Mater.

[151] Huck W.T.S. (2007). Self-assembly meets nanofabrication: Recent developments in microcontact printing and dip-pen nanolithography. Angew. Chem. Int. Ed.

[152] Kelby T.S., Huck W.T.S. (2010). Controlled bending of microscale aupolyelectrolyte brush bilayers. Macromolecules.

[153] Huang H.L., Chen J.K., Houng M.P. (2012). Using soft lithography to fabricate gold nanoparticle patterns for bottom-gate field effect transistors. Thin Solid Films.

[154] Olivier A., Meyer F., Desbief S., Raquez J.M., Verge P., Lazzaroni R., Damman P. (2011). Dubois, pH. Reversible positioning at submicrometre scale of carbon nanotubes mediated by pH-sensitive poly(aminomethacrylate) patterns. Chem. Commun.

[155] Chen T., Chang D.P., Zauscher S. (2010). Fabrication of patterned polymer brushes on chemically active surfaces by *in situ* hydrogen-bondmediated attachment of an initiator. Small.

[156] Zhou F., Zheng Z., Yu B., Liu W., Huck W.T.S. (2006). Multi-component polymer brushes. J. Am. Chem. Soc.

[157] Toshinori F., Andrea D., Letizia V., Barbara M., Virgilio M. (2012). Inkjet printing of protein microarrays on freestanding polymeric nanofilms for spatio-selective cell culture environment. Biomed. Microdevices.

[158] Anke P., Frits D., Adrie R., Ton W., Henk S. (2008). Quality control of inkjet technology for DNA microarray fabrication. Biotechnol. J.

[159] Chang C.J., Wu F.M., Chang S.J., Hsu M.W. (2004). Influence of UV-curable compositions and rib properties on ink-jet-type color filter performance. Jpn. J. Appl. Phys.

[160] Chang C.J., Hung S.T., Lin C.K., Chen C.Y., Kuo E.H. (2010). Selective growth of ZnO nanorods for gas sensors using ink-jet printing and hydrothermal processes. Thin Solid Films.

[161] Chang C.J., Hsu M.H., Weng Y.C., Tsay C.Y., Lin C.K. (2013). Hierarchical ZnO nanorod-array films with enhanced photocatalytic performance. Thin Solid Films.

[162] Chang C.J., Chang S.J., Shih K.C., Pan F.L. (2005). Improving mechanical properties and chemical resistance of ink-jet printer color filter by using diblock polymeric dispersants. J. Polym. Sci. Polym. Phys.

[163] Barry R.A., Shepherd R.F., Hanson J.N., Nuzzo R.G., Wiltzius P., Lewis J.A. (2009). Direct-write assembly of 3D hydrogel scaffolds for guided cell growth. Adv. Mater.

[164] Chang C.J., Chang S.J., Tsou S., Chen S.I., Wu F.M., Hsu M.W. (2003). Effects of polymeric dispersants and surfactants on the dispersing stability and high-speed-jetting properties of aqueous-pigment-based ink-jet inks. J. Polym. Sci. B Polym. Phys.

[165] Chang C.J., Chang S.J., Wu F.M., Hsu M.W., Chiu W.W.W., Chen K. (2004). Effect of compositions and surface treatment on the jetting stability and color uniformity of ink-jet printed color filter. Jpn. J. Appl. Phys.

[166] Chang C.J., Lin Y.H., Tsai H.Y. (2011). Synthesis and properties of UV-curable hyperbranched polymers for ink-jet printing of color micropatterns on glass. Thin Solid Films.

[167] Chang C.J., Tsai M.H., Kao P.C., Tzeng H.Y. (2008). Optical and mechanical properties of jet printed and UV cured blue pixels with phosphated epoxy acrylate as the curing agent. Thin Solid Films.

[168] Chang C.J., Tzeng H.Y. (2006). Preparation and properties of waterborne dual curable monomers and cured hybrid polymers for ink-jet applications. Polymer.

[169] Chang C.J., Tsai H.Y., Hsieh C.C., Chiu W.Y. (2013). Improved chemical resistance of ink-jet printed micropatterns on glass by using dual-functional compositions. J. Appl. Polym. Sci.

[170] Wang L., Wang J., Huang Y., Liu M., Kuang M., Li Y., Jiang L., Song Y. (2012). Inkjet printedcolloidal photonic crystal microdot with fast response induced by hydrophobic transition of poly(N-isopropyl acrylamide). J. Mater. Chem.

[171] Bietsch A., Hegner M., Lang H.P., Gerber C. (2004). Inkjet deposition of alkanethiolate monolayers and DNA oligonucleotides on gold: evaluation of spot uniformity by wet etching. Langmuir.

[172] Sankhe A.Y., Booth B.D., Wiker N.J., Kilbey S.M. (2005). Inkjet-printed monolayers as platforms for tethered polymers. Langmuir.

[173] Emmerling S.G.J., Langer L.B.N., Pihan S.A., Lellig P., Gutmann J.S. (2010). Patterning of a surface immobilized atrp initiator with an inkjet printer. Macromolecules.

[174] Yoshida R., Uchida K., Kaneko Y., Sakai K., Kikuchi A., Sakurai Y., Okano T. (1995). Comb-type grafted hydrogels with rapid de-swelling response to temperature changes. Nature.

[175] Chen J.K., Li J.Y. (2011). Synthesis of tethered poly(N-isopropylacrylamide) for detection of breast cancer recurrence DNA. J. Colloid Interface Sci.

[176] Grover G.N., Braden R.L., Christman K.L. (2013). Oxime cross-linked injectable hydrogels for catheter delivery. Adv. Mater.

[177] Qtaishat M., Khayet M., Matsuura T. (2009). Novel porous composite hydrophobic/hydrophilic polysulfone membranes for desalination by direct contact membrane distillation. J. Membr. Sci.

[178] Chen J.K., Chan C.H., Chang F.C. (2008). Immobilization of layered double hydroxides in the fluidic system for nanoextraction of specific DNA molecules. Appl. Phys. Lett.

[179] Chan C.H., Chen J.K., Chang F.C. (2008). Specific DNA extraction through fluid channels with immobilization of layered double hydroxides on polycarbonate surface. Sens. Actuat. B.

[180] Chen J.K., Li J.Y. (2010). Detection of specific DNA using a microfluidic device featuring tethered poly(N-isopropylacrylamide) on a silicon substrate. Appl. Phys. Lett.

[181] Chen J.K., Li J.Y. (2010). Fabrication of DNA extraction device with tethered poly(N-isopropylacrylamide) brushes on silicon surface for a specific DNA detection. Sens. Actuat. B.

[182] Hosoya K., Kubo T., Tanaka N., Haginaka J. (2003). A possible purification method of DNAs’ fragments from humic matters in soil extracts using novel stimulus responsive polymer adsorbent. J. Pharm. Biomed. Anal.

[183] Anastase-Ravion S., Ding Z., Pelle A., Hoffman A.S., Letourneur D. (2001). New antibody purification procedure using a thermally responsive poly(N-isopropylacrylamide)-dextran derivative conjugate. J. Chromatogr. B.

[184] Kuboi R., Morita S., Ota H., Umakoshi H. (2000). Protein refolding using stimuli-responsive polymer-modified aqueous twophase systems. J. Chromatogr. B.

[185] Lu Y., Mei Y., Drechsler M., Ballauff M. (2006). Thermosensitive core–shell particles as carriers for ag nanoparticles: modulating the catalytic activity by a phase transition in networks. Angew. Chem. Int. Ed.

[186] Ionov L., Stamm M., Diez S. (2006). Reversible switching of microtubule motility using thermoresponsive polymer surfaces. Nano Lett.

[187] Ionov L., Stamm M., Diez S. (2005). Size sorting of protein assemblies using polymeric gradient surfaces. Nano Lett.

[188] Minko S. (2006). Responsive polymer brushes. Polym. Rev.

[189] Li X., Yu X.H., Han Y.C. (2012). Intelligent reversible nanoporous antireflection film by solvent-stimuli-responsive phase transformation of amphiphilic block copolymer. Langmuir.

[190] Wan P.B., Eric H.H., Zhang X. (2012). Interfacial supramolecular chemistry for stimuli-responsive functional surfaces. Prog. Chem.

[191] Retsos H., Gorodyska G., Kiriy A., Stamm M., Creton C. (2005). Adhesion between chemically heterogeneous switchable polymeric brushes and an elastomeric adhesive. Langmuir.

[192] Ionov L., Houbenov N., Sidorenko A., Stamm M., Minko S. (2009). Stimuli-responsive command polymer surface for generation of protein gradients. Biointerphases.

[193] Ionov L., Houbenov N., Sidorenko A., Stamm M., Minko S. (2006). Smart microfluidic channels. Adv. Funct. Mater.

[194] Hinrichs K., Aulich D., Ionov L., Esser N., Eichhorn K.J., Motornov M., Stamm M., Minko S. (2009). Chemical and structural changes in a pH-responsive mixed polyelectrolyte brush studied by infrared ellipsometry. Langmuir.

[195] Houbenov N., Minko S., Stamm M. (2003). Mixed Polyelectrolyte Brush from Oppositely Charged Polymers for Switching of Surface Charge and Composition in Aqueous Environment. Macromolecules.

[196] Berger S., Synytska A., Ionov L., Eichhorn K.L., Stamm M. (2008). Stimuli-Responsive Bicomponent Polymer Janus Particles by “Grafting from”/“Grafting to” Approaches. Macromolecules.

[197] Chen J.K., Bai B.J., Chang F.C. (2011). Diagnosis of breast cancer recurrence using a microfluidic device featuring tethered cationic polymers. Appl. Phys. Lett.

[198] Chen J.K., Bai B.J. (2011). Diagnosis of breast cancer recurrence after surgery by using poly(2-dimethylaminoethyl methacrylate) brushes as a medium on silicon surface. Sens. Actuat. B Chem.

[199] Beebe D.J., Moore J.S., Bauer J.M., Yu Q., Liu R.H., Devadoss C., Jo B.H. (2000). Functional hydrogel structures for autonomous flow control inside microfluidic channels. Nature.

[200] Zeng X.F., Jiang H.R. (2008). Tunable liquid microlens actuated by infrared light-responsive hydrogel. Appl. Phys. Lett.

[201] Richter A., Klatt S., Paschew G., Klenke C. (2009). Micropumps operated by swelling and shrinking of temperature-sensitive hydrogels. Lab. Chip.

[202] Gopishetty V., Roiter Y., Tokarev I., Minko S. (2008). Multiresponsive biopolyelectrolyte membrane. Adv. Mater.

[203] Chen J.K., Chen J.Y., Lin H.C., Hong P.D., Chang F.C. (2009). Patterned poly(2-hydroxyethyl methacrylate) brushes on silicon surfaces behave as “tentacles” to capture ferritin from aqueous solution. ACS Appl. Mater. Interfaces.

[204] Chen Y., Wang R., Zhou J.A., Fan H.J., Shi B. (2011). On-demand drug delivery from temperature-responsive polyurethane membrane. React. Funct. Polym.

[205] Alem H., Duwez A.S., Lussis P., Lipnik P., Jonas A.M., Demoustier-Champagne S. (2008). Microstructure and thermo-responsive behavior of poly (N-isopropylacrylamide) brushes grafted in nanopores of track-etched membranes. J. Membr. Sci.

[206] Liu P., Xiang L.B., Tan Q., Tang H.Y., Zhang H.L. (2013). Steric hindrance effect on thermoresponsive behaviors of pyrrolidone-based polymers. Polym. Chem.

[207] Takahashi H., Nakayama M., Yamato M., Okano T. (2010). Controlled chain length and graft density of thermoresponsive polymer brushes for optimizing cell sheet harvest. Biomacromolecules.

[208] Vertommen M.A.M.E., Cornelissen H.J.L., Dietz C.H.J.T., Hoogenboom R., Kemmere M.F., Keurentjes J.T.F. (2008). Pore-covered thermoresponsive membranes for repeated on-demand drug release. J. Membr. Sci.

[209] Okahata Y., Noguchi H., Seki T. (1986). Thermoselective permeation from a polymer-grafted capsule membrane. Macromolecules.

[210] Cui Y., Tao C., Zheng S.P., He Q., Ai S.F., Li J.B. (2005). Synthesis of thermosensitive PNIPAM-co-MBAA nanotubes by atom transfer radical polymerization within a porous membrane. Macromol. Rapid Commun.

[211] Rama Rao G.V., Lopez G.P. (2000). Encapsulation of poly(NIsopropyl acrylamide) in silica: A stimuli-responsive porous hybrid material that incorporates molecular nano-valves. Adv. Mater.

[212] Rama Rao G.V., Krug M.E., Balamurugan S., Xu H., Xu Q., López G.P. (2002). Synthesis and characterization of silica-poly(N-isopropylacrylamide) hybrid membranes: Switchable molecular filters. Chem. Mater.

[213] Wu A.S., Hoffman A.S., Yager P.J. (1992). Synthesis and characterization of thermally reversible macroporous poly(N-isopropylacrylamide) hydrogels. J. Polym. Sci. Polym. Chem.

[214] Hester J.H., Banerjee P., Mayes A.M. (1999). Preparation of protein-resistant surfaces on poly(vinylidene fluoride) membranes via surface segregation. Macromolecules.

[215] Ying L., Kang E.T., Neoh K.G. (2002). Synthesis and characterization of poly(N-isopropylacrylamide)-graft-poly(vinylidene fluoride) copolymers and temperature-sensitive membranes. Langmuir.

[216] Okano T., Kikuchi A., Sakurai Y., Takei Y., Ogata N. (1995). Temperature-responsive poly (N-isopropylacrylamide) as a modulator for alteration of hydrophilic/hydrophobic surface properties to control activation/inactivation of platelets. J. Control. Release.

[217] Nandkumar M.A., Yamato M., Kushida A., Konno C., Hirose M., Kikuchi A., Okano T. (2002). Two-dimensional cell sheet manipulation of heterotypically co-cultured lung cells utilizing temperature-responsive culture dishes results in long-term maintenance of differentiated epithelial cell functions. Biomaterials.

[218] Uchida K., Sakai K., Ito E., Kwon O.H., Kikuchi A., Yamato M., Okano T. (2000). Temperature-dependent modulation of blood platelet movement and morphology on poly(N-isopropylacrylamide)-grafted surfaces. Biomaterials.

[219] Yamato M., Konno C., Kushida A., Hirose M., Utsumi M., Kikuchi A., Okano T. (2000). Release of adsorbed fibronectin from temperature-responsive culture surfaces requires cellular activity. Biomaterials.

[220] Nakajima K., Honda S., Nakamura Y., Redondo F.L.H., Kohsaka S., Yamato M., Kikuchi A., Okano T. (2001). Intact microglia are cultured and non-invasively harvested without pathological activation using a novel cultured cell recovery method. Biomaterials.

[221] Kim M.R., Jeong J.H., Park T.G. (2002). Swelling Induced detachment of chondrocytes using RGD-modified poly(N-isopropylacrylamide) hydrogel beads. Biotechnol. Prog.

[222] Ebara M., Yamato M., Hirose M., Aoyagi T., Kikuchi A., Sakai K., Okano T. (2003). Copolymerization of 2-carboxyisopropylacrylamide with N-isopropylacrylamide accelerates cell detachment from grafted surfaces by reducing temperature. Biomacromolecules.

[223] Uakushiji T., Sakai K., Kikuchi A., Aoyagi T., Sakurai Y., Okano T. (1998). Graft architectural effects on thermo-responsive wettability changes of poly(N-isopropylacrylamide)-modified surfaces. Langmuir.

[224] Chen J.K., Wang J.H., Chang J.Y., Fan S.K. (2012). Thermally switchable adhesions of polystyrene-block-poly (*n*-isopropylacrylamide) copolymer pillar array mimicking climb attitude of geckos. Appl. Phys. Lett.

[225] Chen J.K., Qui J.Q. (2012). Patterned 3D Assembly of Au nanoparticle on silicon substrate by colloid lithography. J. Nanopart. Res.

[226] Bruinink C.M., Peter M., Maury P.A., de Boer M., Kuipers L., Hukens J., Reinhoudt D.N. (2006). Capillary force lithography: Fabrication of functional polymer templates as versatile tools for nanolithography. Adv. Funct. Mater.

[227] Suh K.Y., Lee H.H. (2002). Capillary force lithography: large-area pattering, self-organization, and anisotropic dewetting. Adv. Funct. Mater.

[228] Radha B., Kulkarni G.U. (2009). Dewetting assisted patterning of polystyrene by soft lithography to create nanotrenches for nanomaterial deposition. ACS Appl. Mater. Interfaces.

[229] Yu X., Wang Z., Xing R., Luan S., Han Y. (2005). Fabrication of structures with tunable morphologies and sizes by soft molding. Appl. Surf. Sci.

[230] Kobayashi J., Kikuchi A., Sakai K., Okano T. (2002). Aqueous chromatography utilizing hydrophobicity-modified anionic temperature-responsive hydrogel for stationary phases. J. Chromatogr. A.

[231] Vancea I., Thiele U., Pauliac-Vaujour E., Stannard A., Martin C.P., Blunt M.O., Moriarty P.J. (2008). Front instabilities in evaporatively dewetting nanofluids. Phys. Rev. E.

[232] Chang C.J., Kuo E.H. (2010). Roughness-enhanced thermal-responsive surfaces by surface-initiated polymerization of polymer on ordered ZnO pore-array films. Thin Solid Films.

[233] Huanga H.L., Chenb J.K., Houng M.P. (2013). Fabrication of two-dimensional periodic relief grating of tethered polystyrene on silicon surface as solvent sensors. Sens. Actuat. B.

[234] Kikuta H., Ohira Y., Kubo H., Iwata K. (1998). Effective medium theory of two-dimensional subwavelength gratings in the non-quasi-static limit. J. Opt. Soc. Am. A.

[235] Lalanne P., Lalanne D.L. (1996). ‘On the effective medium theory of subwavelength periodic structures Design and fabrication of blazed binary diffractive elements with sampling periods smaller than the structural cutoff. J. Mod. Opt.

[236] Chen J.K., Bai B.J. (2011). pH-switchable optical properties of the one-dimensional periodic grating of tethered poly(2-dimethylaminoethyl methacrylate) brushes on a silicon surface. J. Phys. Chem. C.

[237] Chen J.K., Qui J.Q. (2011). Nanowires of 3-D cross-linked gold nanoparticle assemblies behave as thermosensors on silicon substrates. Colloid Polym. Sci.

[238] Ye G., Wang X. (2010). Amperometric glucose biosensor based on a truangular silver nanoprisms/chitosan composite film as immobilization matrix. Biosens. Bioelectron.

[239] Chen J.K., Wang J.H., Cheng C.C., Chang J.Y. (2013). Reversibly thermoswitchable two-dimensional periodic gratings prepared from tethered poly(N-isopropylacrylamide) on silicon surfaces. ACS Appl. Mater. Interfaces.

[240] Chen J.K., Wang J.H., Cheng C.C., Chang J.W., Chang F.C. (2013). Polarity-indicative two-dimensional periodic relief gratings of tethered poly(methyl methacrylate) on silicon surfaces for visualization in volatile organic compound sensing. Appl. Phys. Lett.

[241] Chen J.K., Wang J.H., Chang C.J., Huang C.F. (2013). Polarity-indicative two-dimensional periodic concave gratings of tethered polystyrene on silicon surfaces for visualization in VOC sensing. Sens. Actuat. B.

[242] Matsubara K., Watanabe M., Takeoka Y. (2007). Thermally adjustable multicolor photochromic hydrogel. Angew. Chem. Int. Ed.

[243] Jeong K.U., Jang J.H., Koh C.Y., Graham M.J., Jin K.Y., Park S.J., Nah M.C., Lee H., Cheng Z.D., Thomas E.L. (2009). Colour-tunable spiral photonic actuators. J. Mater. Chem.

[244] Lawrence J.R., Shim G.H., Jiang P., Han M.G., Ying Y.R., Foulger S.H. (2005). Direct correlation of organic semiconductor film structure to field-effect mobility. Adv. Mater.

[245] Ge J.P., Hu Y.X., Yin Y.D. (2007). Highly tunable superparamagnetic colloidal photonic crystals. Angew. Chem. Int. Ed.

[246] Ueno K., Sakamoto J., Takeoka Y., Watanabe M. (2009). Electrochromism based on structural colour changes in a polyelectrolyte gel. J. Mater. Chem.

[247] Walish J.J., Kang Y. (2009). Bioinspired electrochemically tunable block copolymer full color pixels. Adv. Mater.

[248] Crookes W.J., Ding L.L., Huang Q.L., Kimbell J.R., Horwitz J., McFall-Ngai M.J. (2004). Reflectins: The unusual proteins of squid reflective tissues. Science.

[249] Kim J., Serpe M.J., Lyon L.A. (2004). Hydrogel microparticles as dynamically tunable microlenses. J. Am. Chem. Soc.

[250] Wu H., Odom T.W., Whitesides G.M. (2002). Connectivity of features in microlens array reduction photolithography: Generation of various patterns with a single photomask. J. Am. Chem. Soc.

[251] Dong L., Agarwal A.K., Beebe D.J., Jiang H. (2006). Adaptive liquid microlenses activated by stimuli-responsive hydrogels. Nature.

[252] Holmes D.P., Crosby A.J. (2007). Snapping surfaces. Adv. Mater.

[253] Akashi R., Tsutsui H., Komura A. (2002). Polymer gel light-modulation materials imitating pigment cells. Adv. Mater.

[254] Hoffman A.S. (2000). Bioconjugation of intelligent polymers and recognition proteins for use in diagnostics and affinity separations. Clin. Chem.

[255] Wang J., Jiang M. (2000). Toward genolelectronics: Nucleic acid doped conducting polymers. Langmuir.

[256] Garnier F., Korri-Youssoufi H., Srivastava P., Mandrand B., Delair T. (1999). Toward intelligent polymers: DNA sensors based on oligonucleotide-functionalized polypyrroles. Synth. Met.

[257] Livache T., Fouque B., Roget A., Marchand J., Bidan G., Teoule R., Mathis G. (1998). Polypyrrole DNA chip on a silicon device: example of hepatitis C virus genotyping. Anal. Biochem.

[258] Tang Z., Mori T., Takarada T., Maeda M. (2001). Single nucleotide polymorphisms (SNPs) assay using reversible association and dispersion of DNA-linked colloidal nanoparticles. Nucleic Acids Res. Suppl.

[259] Chen J.K., Zhou G.Y., Huang C.F., Chang J.Y. (2013). Two-dimensional periodic relief grating as a versatile platform for selective immunosorbent assay and visualizing of antigens. ACS Appl. Mater. Interfaces.

[260] Holt D.B., Gauger P.R., Kusterbeck A.W., Ligler F.S. (2002). Fabrication of a capillary immunosensor in polymethyl methacrylate. Biosens. Bioelectron.

[261] Canavan H.E., Cheng X., Graham D.J., Ratner B.D., Castner D.G. (2005). Cell sheet detachment affects the extracellular matrix: A surface science study comparing thermal liftoff, enzymatic, and mechanical methods. J. Biomed. Mater. Res. A.

[262] Chen J.K., Zhou G.Y., Chang C.J. (2013). Real-time multicolor antigen detection with chemo responsive diffraction gratings of silicon oxide nanopillar arrays. Sens. Actuat. B.

[263] Chen J.K., Zhou G.Y., Huang C.F., Ko F.H. (2013). Using nanopillars of silicon oxide as a versatile platform for visualizing a selective immunosorbent. Appl. Phys. Lett.

[264] El-Ragehy N.A., El-Kosasy A.M., Abbas S.S., El-Khateeb S.Z. (2000). Polymeric membrane electrodes for selective determination of the central nervous system acting drugs fluphenazine hydrochloride and nortriptyline hydrochloride. Anal. Chim. Acta.

[265] Liu Z.H., Wen M.L., Yao Y., Xiong J. (2001). Plastic pethidine hydrochloride membrane sensor and its pharmaceutical applications. Sens. Actuat. B.

[266] Comrie J.E., Huck W.T.S. (2007). Formation of hybrid 2D polymer–metal microobjects. Langmuir.

[267] Tugulu S., Harms M., Fricke M., Volkmer D., Klok H.A. (2006). Polymer brushes as ionotropic matrices for the directed fabrication of microstructured calcite thin films. Angew. Chem. Int. Ed.

[268] Fu Q., Rao G.V.R., Ista L.K., Wu Y., Andrzejewski B.P., Sklar L.A. (2003). Control of molecular transport through stimuliresponsive ordered mesoporous materials. Adv. Mater.

[269] Kim S.Y., Kanamori T., Shinbo T. (2002). Preparation of thermalresponsive poly(propylene) membranes grafted with Nisopropy lacrylamide by plasma-induced polymerization and their water permeation. J. Appl. Polym. Sci.

[270] Shtanko N.I., Kabanov V.Y., Apela P.Y., Yoshida M., Vilenskii A.I. (2000). Preparation of permeability-controlled track membranes on the basis of ‘smart’ polymers. J. Membr. Sci.

[271] Wang W.Y., Chen L., Yu X. (2006). Preparation of temperature sensitive poly(vinylidene fluoride) hollow fiber membranes grafted with N-isopropylacrylamide by a novel approach. J. Appl. Polym. Sci.

[272] Rao G.V.R., Balamurugan S., Meyer D.E., Chilkoti A., Lopez G.P. (2002). Hybrid bioinorganic smart membranes that incorporate protein-based molecular switches. Langmuir.

[273] Peng T., Cheng Y.L. (2000). pH-responsive permeability of PE-g-PMAA membranes. J. Appl. Polym. Sci.

[274] Wang Y., Liu Z., Han B., Dong Z., Wang J., Sun D. (2004). pH sensitive polypropylene porous membrane prepared by grafting acrylic acid in supercritical carbon dioxide. Polymer.

[275] Park S.B., You J.O., Park H.Y., Haam S.J., Kim W.S. (2001). A novel pH-sensitive membrane from chitosan-TEOS IPN; preparation and its drug permeation characteristics. Biomaterials.

[276] Hester J.F., Olugebefola S.C., Mayes A.M. (2002). Preparation of pH responsive polymer membranes by self-organization. J. Membr. Sci.

[277] Zhai L., Nolte A.J., Cohen R.E., Rubner M.F. (2004). pH-gated porosity transitions of polyelectrolyte multilayers in confined geometries and their application as tunable Bragg reflectors. Macromolecules.

[278] Dejugnat C., Sukhorukov G.B. (2004). pH-responsive properties of hollow polyelectrolyte microcapsules templated on various cores. Langmuir.

[279] Savina I.N., Galaev I.Y., Mattiasson B. (2005). Graft polymerization of acrylic acid onto macroporous polyacrylamide gel (cryogel) initiated by potassium diperiodatocuprate. Polymer.

[280] Nakayama H., Kaetsu I., Uchida K., Sakata S., Tougou K., Hara T. (2002). Radiation curing of intelligent coating for controlled release and permeation. Radiat. Phys. Chem.

[281] Zhai G. (2006). pH- and temperature-sensitive microfiltration membranes from blends of poly(vinylidenefluoride)-graftpoly(4-vinylpyridine) and poly(N-isopropyl -acrylamide). J. Appl. Polym. Sci.

[282] Liu X., Neoh K.G., Kang E.T. (2003). Redox-sensitive microporous membranes prepared from poly(vinylidene fluoride) grafted with viologen-containing polymer side chains. Macromolecules.

[283] Zaikin A.N., Zhabotinsky A.M. (1970). Concentration wave propagation in two-dimensional liquid-phase self-oscillating system. Nature.

[284] Murase Y., Maeda S., Hashimoto S., Yoshida R. (2009). Design of a mass transport surface utilizing peristaltic motion of a self-oscillating gel. Langmuir.

[285] Maeda S., Hara Y., Yoshida R., Hashimoto S. (2008). Control of the dynamic motion of a gel actuator driven by the belousov-zhabotinsky reaction. Macromol. Rapid Commun.

[286] Pokroy B., Epstein A.K., Persson-Gulda M.C.M., Aizenberg J. (2009). Fabrication of Bioinspired Actuated Nanostructures with Arbitrary Geometry and Stiffness. Adv. Mater.

[287] Sidorenko A., Krupenkin T., Taylor A., Fratzl P., Aizenberg J. (2007). Reversible Switching of Hydrogel-Actuated Nanostructures into Complex Micropatterns. Science.

[288] Sidorenko A., Krupenkin T., Aizenberg J. (2008). Controlled switching of the wetting behavior of biomimetic surfaces with hydrogel-supported nanostructures. J. Mater. Chem.

[289] Kwon G.H., Park J.Y., Kim J.Y., Frisk M.L., Beebe D.J., Lee S.H. (2008). Biomimetic Soft Multifunctional Miniature Aquabots. Small.

[290] Thérien-Aubin H., Wu Z.L., Nie Z., Kumacheva E. (2013). Multiple shape transformations of composite hydrogel sheets. J. Am. Chem. Soc.

[291] Ionov L. (2012). Biomimetic 3D self-assembling biomicroconstructs by spontaneous deformation of thin polymer films. J. Mater. Chem.

[292] Kim J., Hanna J.A., Hayward R.C., Santangelo C.D. (2012). Thermally responsive rolling of thin gel strips with discrete variations in swelling. Soft Matter.

[293] Eddington D.T., Beebe D.J. (2004). Flow control with hydrogels. Adv. Drug Deliv. Rev.

[294] Pelton R. (2000). Temperature-sensitive aqueous microgels. Adv. Colloid Interface Sci.

[295] Richter A., Howitz S., Kuckling D., Arndt K.F. (2004). Influence of volume phase transition phenomena on the behavior of hydrogel-based valves. Sens. Actuat. B.

[296] Buchholz B.A., Doherty A.S., Albarghouthi M.N., Bogdan F.M., Zahn J.M., Barron A.E. (2001). Microchannel DNA sequencing matrices with a thermally controlled “viscosity switch”. Anal. Chem.

[297] Harmon M.E., Tang M., Frank C.W. (2003). A microfluidic actuator based on thermoresponsive hydrogel. Polymer.

